# Clinical effectiveness of vaginal pessary self-management vs clinic-based care for pelvic organ prolapse (TOPSY): a randomised controlled superiority trial

**DOI:** 10.1016/j.eclinm.2023.102326

**Published:** 2023-11-23

**Authors:** Suzanne Hagen, Rohna Kearney, Kirsteen Goodman, Catherine Best, Andrew Elders, Lynn Melone, Lucy Dwyer, Melanie Dembinsky, Margaret Graham, Wael Agur, Suzanne Breeman, Jane Culverhouse, Angela Forrest, Mark Forrest, Karen Guerrero, Christine Hemming, Aethele Khunda, Sarkis Manoukian, Helen Mason, Doreen McClurg, John Norrie, Ranee Thakar, Carol Bugge

**Affiliations:** a Nursing, Midwifery & Allied Health Professions Research Unit, Glasgow Caledonian University, Glasgow, UK; b The Warrell Unit, Saint Mary’s Hospital, Manchester, UK; c Division of Developmental Biology & Medicine, School of Medical Sciences, University of Manchester, UK; d Faculty of Health Sciences & Sport, University of Stirling, Stirling, UK; e Manchester Academic Health Science Centre, Manchester, UK; f Department of Nursing Health, Glasgow Caledonian University, Glasgow, UK; g Patient and Public Involvement Partner, Dunlop, UK; h Department of Gynaecology, University Crosshouse Hospital, UK; i Centre for Healthcare Randomised Trials, University of Aberdeen, Aberdeen, UK; j Patient and Public Involvement Partner, Manchester, UK; k Patient and Public Involvement Partner, Milton Keynes, UK; l Department of Urogynaecology, Queen Elizabeth University Hospital, UK; m Department of Gynaecology, Aberdeen Royal Infirmary, Aberdeen, UK; n Department of Urogynaecology, The James Cook University Hospital, Middlesbrough, UK; o Yunus Centre for Social Business & Health, Glasgow Caledonian University, Glasgow, UK; p Edinburgh Clinical Trials Unit, University of Edinburgh, UK; q Department of Urogynaecology, Croydon University Hospital, Croydon, UK

**Keywords:** Pelvic organ prolapse, Pessary, Self-management, Randomised controlled trial

## Abstract

**Background:**

Prolapse affects 30–40% of women. Those using a pessary for prolapse usually receive care as an outpatient. This trial determined effectiveness and cost-effectiveness of pessary self-management (SM) vs clinic-based care (CBC) in relation to condition-specific quality of life (QoL).

**Methods:**

Parallel-group, superiority randomised controlled trial, recruiting from 16 May 2018 to 7 February 2020, with follow-up to 17 September 2021. Women attending pessary clinics, ≥18 years, using a pessary (except Shelf, Gellhorn or Cube), with pessary retained ≥2 weeks were eligible. Limited manual dexterity; cognitive deficit; pregnancy; or requirement for non-English teaching were exclusions. SM group received a 30-min teaching session; information leaflet; 2-week follow-up call; and telephone support. CBC group received usual routine appointments. The primary clinical outcome was pelvic floor-specific QoL (PFIQ-7), and incremental net monetary benefit for cost-effectiveness, 18 months post-randomisation. Group allocation was by remote web-based application, minimised on age, user type (new/existing) and centre. Participants, intervention deliverers, researchers and the statistician were not blinded. The primary analysis was intention-to-treat based. Trial registration: https://doi.org/10.1186/ISRCTN62510577.

**Findings:**

The requisite 340 women were randomised (169 SM, 171 CBC) across 21 centres. There was not a statistically significant difference between groups in PFIQ-7 at 18 months (mean SM 32.3 vs CBC 32.5, adjusted mean difference SM-CBC −0.03, 95% CI −9.32 to 9.25). SM was less costly than CBC. The incremental net benefit of SM was £564 (SE £581, 95% CI −£576 to £1704). A lower percentage of pessary complications was reported in the SM group (mean SM 16.7% vs CBC 22.0%, adjusted mean difference −3.83%, 95% CI –6.86% to −0.81%). There was no meaningful difference in general self-efficacy. Self-managing women were more confident in self-management activities. There were no reported suspected unexpected serious adverse reactions, and 31 unrelated serious adverse events (17 SM, 14 CBC).

**Interpretation:**

Pessary self-management is cost-effective, does not improve or worsen QoL compared to CBC, and has a lower complication rate.

**Funding:**

10.13039/501100000272National Institute for Health and Care Research, Health Technology Assessment Programme (16/82/01).

**Research in context**

**Evidence before this study**

There have been no previous randomised trials evaluating pessary self-management, as confirmed in the 2023 International Consultation on Incontinence evidence review on conservative management of prolapse undertaken by the lead author (search dates: 9th September 2015 to 31st December 2020). Non-randomised studies have indicated self-management is associated with: higher levels of convenience, ability to access help, support and comfort; lower adverse event rates; and greater likelihood of continuing pessary use. These studies suggested that self-management was a viable treatment option, but effectiveness had not been evaluated.

**Added value of this study**

This is the only trial to date comparing pessary self-management with clinic-based care for management of prolapse. As such, it provides the first randomised evidence on effectiveness and cost-effectiveness to guide practice and service provision.

**Implications of all the available evidence**

This trial demonstrated comparable quality of life for women who self-managed and those who received clinic-based pessary care, with fewer complications and less health care resource use and cost. These findings support routinely offering women who can self-manage the option to do so. This is currently not widespread practice and if adopted would potentially decrease health care resource use and reduce unpleasant pessary complications for women.

## Introduction

Pelvic organ prolapse is the descent of one or more of the anterior vaginal wall, posterior vaginal wall, uterus or vaginal vault, with associated symptoms.^1^ Prolapse is a common problem, with studies suggesting that 30–40% of women have prolapse on examination, 5–10% report symptoms, and there is a 9.5% lifetime risk of prolapse surgery.^2^^,^^3^ Prolapse prevalence increases with age and presents a growing health problem.^4^ Women with prolapse report bothersome symptoms that negatively affect their quality of life and body image.^5^ Symptoms include a feeling of ‘something coming down’ or ‘bulge’ in the vagina, urinary, bowel and sexual symptoms and discomfort.

Prolapse can be treated conservatively or surgically. Up to 18.5% of women who have prolapse surgery may need a further prolapse repair procedure^2^ and the recent controversy around the use of surgical mesh has resulted in a focus on conservative treatment options. One conservative treatment option is vaginal pessary. The pessary is an inexpensive mechanical device that is inserted into the vagina to support the pelvic organs. Pessaries are used worldwide with two thirds of women initially choosing a vaginal pessary to treat prolapse symptoms.^6^^,^^7^

For women who use a pessary as treatment for prolapse it is usually fitted at a gynaecological clinic, or occasionally at a GP surgery, and the woman returns approximately six-monthly for follow-up. However, having to return to clinic every six months may be inconvenient for women, and uninterrupted placement of a pessary may increase the risk of complications (e.g., discharge, erosion, bleeding).^8^ It may interfere with sexual intercourse, and the woman may require a review in clinic more frequently if the pessary is expelled or pessary complications are experienced (e.g., bleeding, vaginal discomfort). An alternative to clinic-based care is pessary self-management where a woman removes and reinserts the pessary herself, thus offering her more control to maintain and improve her own health. Research in other clinical domains suggests that self-management is beneficial because people improve their self-efficacy (confidence) to look after their own health.^9^ There is no evidence on the effectiveness of pessary self-management for women with prolapse. The current trial aimed to evaluate the effectiveness and cost-effectiveness of self-management of a vaginal pessary on the pelvic floor-specific quality of life of women with pelvic organ prolapse when compared to clinic-based care.

## Methods

### Study design

We undertook a parallel group multicentre randomised controlled trial, with individual randomisation (allocation ratio 1:1), which assessed the superiority of pessary self-management compared to clinic-based care for women who used a pessary for prolapse. Full details of the trial protocol have been published previously.^10^ An economic evaluation and mixed methods process evaluation (assessing intervention acceptability, pathways to effectiveness, adherence to treatment and fidelity),^11^ were undertaken concurrently and will be reported in full elsewhere.

### Participants

Recruitment took place in healthcare settings in the UK National Health Service. Participants were recruited from 21 centres where pessary care was routinely provided. Women who were new pessary users (had used a pessary for 3 months or less) and existing users (had used a pessary for longer than 3 months, currently or in the recent past) were identified by centre staff via patient notes, clinic lists, caseloads and referral letters. Potentially eligible women were approached by their centre, either by letter or in clinic, and their details entered on a screening log. Women were eligible for inclusion if they were 18 years or older, were using a pessary of any material or type (except Shelf, Gellhorn or cube pessary) and they had successfully retained the pessary for at least two weeks. Women were excluded if they had limited manual dexterity that would impede their ability to remove and replace their own pessary; were judged by their healthcare team to have a cognitive deficit such that it was not possible for them to give informed consent or to self-manage; were pregnant; or had insufficient understanding of English language (the self-management teaching was only available in English).

### Randomisation and masking

Allocation was carried out remotely via a web-based computerised system developed and maintained by the Centre for Healthcare Randomised Trials, University of Aberdeen, with minimisation on age (<65/≥65 years), pessary user type (new/existing user) and centre. Centre staff accessed the system to allocate eligible, consenting women to their group. Participants, intervention deliverers, researchers and the statistician were not masked to group allocation.

### Procedures

Trial centres received a training visit during which the research team explained the principles of self-management and trained the intervention delivery staff (specialist nurse, physiotherapist or doctor) in the components of the intervention.^12^ Each centre also received a training manual which provided guidance on the intervention. Participants randomised to self-management received:

• a 30-min self-management teaching appointment with a trained healthcare professional where they were taught and given the opportunity to remove, clean and reinsert their own pessary;
• an information leaflet which provided written and diagrammatic information on pessary self-management;
• a 2-week follow-up telephone call to establish whether they had been able to remove, clean and reinsert the pessary since the teaching appointment, and any difficulties they experienced;
• a telephone support number for their local clinical centre.

Women in the clinic-based care group received usual care comprising appointments where their pessary was removed, cleaned and re-inserted or renewed by a healthcare professional. The frequency of appointments was determined by the usual practice of the centre, with most centres seeing women every 6 months. Median pessary change interval in the clinic-based care group was 186 days (IQR 165–201) (excluding Covid-19 lockdown periods).

Recruitment was complete prior to the start of the Covid-19 pandemic, however the associated lockdown necessitated some adjustments to intervention delivery and data collection methods that are detailed in the Statistical Analysis Plan and the forthcoming final report.

### Adherence

Levels of adherence to study protocol in each group were assessed. Women in the self-management group were defined as being ‘on treatment’ if at 18 months they were using a pessary, had received trial-specific self-management teaching and reported inserting their pessary at any follow-up time-point. In the clinic-based care group ‘on treatment’ was defined as using a pessary at 18 months and not having reported inserting their pessary at any follow-up time-point.

### Outcomes

The primary clinical outcome measure for effectiveness was pelvic floor-specific quality of life at 18 months, measured using the Pelvic Floor Impact Questionnaire-7 (PFIQ-7), a validated, participant-completed measure with three subscales (Urinary Impact Questionnaire (UIQ-7), Colorectal-Anal Impact Questionnaire (CRAIQ-7), Pelvic Organ Prolapse Impact Questionnaire (POPIQ-7)), each ranging from 0 to 100, total score ranging from 0 to 300 (higher scores reflect worse pelvic floor-specific quality of life).^13^ The primary outcome for cost-effectiveness was the incremental net monetary benefit at 18 months. Resource use data (primary and secondary care use e.g., GP and hospital appointments, clinic appointments and prescribed drugs) were collected using a specifically developed Health Resource Use Questionnaire.

Secondary outcome measures were: the EuroQol EQ-5D-5L questionnaire (range −0.594 to 1) and EQ-5D visual analogue scale (range 0–100), higher scores reflect better generic health-related quality of life^14^; the Pelvic Floor Distress Inventory-20 (PFDI-20) (range 0–300, 3 subscales (Urinary Distress Inventory (UDI-6), Colorectal-Anal Distress Inventory (CRADI-8), Pelvic Organ Prolapse Distress Inventory (POPDI-6)) each ranging 0–100), higher scores indicating more severe bladder, bowel and prolapse symptoms^13^; the Prolapse Incontinence Sexual Questionnaire-IUGA Revised version (PISQ-IR), sub-score means range 1–5, higher scores indicating better sexual function^15^; the Patient Global Impression of Improvement (PGI-I), a single item measure adapted to assess perception of pessary care within the trial (7 responses from “very much better” to “very much worse”)^16^; the General Self Efficacy Scale, range from 10 to 40, higher scores reflect greater self-efficacy^17^; study-specific questionnaires for pessary complications (15 items, percentage of relevant items reported calculated for each participant), pessary use (9 items) and pessary confidence (6 items); uptake of additional treatment for prolapse; uptake of telephone support related to pessary use; and health of vaginal tissues (inflammation of vaginal tissues, ulceration, granulation and any other clinical concerns).

The secondary outcomes detailed in the trial registry were updated in the protocol to include newer versions of the EQ-5D and the PISQ, and to add telephone support uptake and vaginal tissue health outcomes.^10^ Outcome data were collected by participant-completed questionnaires at baseline, 6, 12 and 18 months (a choice of paper or electronic questionnaire was offered at follow-up time-points), and vaginal examination by a healthcare professional at baseline and 18 months post-randomisation to identify problems associated with pessary use.

Participants in the clinic-based care group were asked about the occurrence of adverse and serious adverse events at every pessary follow-up appointment, whilst those in the self-management group were asked during the teaching appointment, and advised in the self-management information leaflet to call the telephone support number if they experienced symptoms indicative of an Adverse Event (AE) or Serious Adverse Event (SAE). All participants were asked about pessary complications in questionnaires at each time-point. Six, 12 and 18-month questionnaires asked participants about admissions to hospital, any accidents and new or changed medication regimens. The following events arising from pessary treatment are common and were not considered adverse events: granulation of vaginal tissue, involuntary expulsion of pessary, vaginal smell, vaginal discharge and bleeding during pessary change.

### Statistics

A sample size of 330 women (165 per group) was required to provide 90% power to detect a difference of 20 points in the PFIQ−7 score at 18 months after randomisation, assuming a standard deviation of 50, two-sided alpha of 0.05, and 20% loss to follow-up. Although there was no published minimal clinically important difference for the PFIQ-7 in this population, wide consultation with PPI representatives and clinical collaborators led to consensus on a 20-point difference being meaningful. Analysis was carried out in accordance with the Statistical Analysis Plan (SAP).

The main analysis was an intention-to-treat (ITT) analysis, i.e., all randomised participants were included and analysed by treatment as allocated. Missing PFIQ-7 responses were assumed to be Missing At Random. Difference between groups was tested by longitudinal analysis of covariance^18^ adjusted for age group, pessary user type and baseline scores. The models fitted random effects for centre and participant with a compound symmetry structure. Estimates of treatment effect size were expressed as the linear combination of the fixed effect solutions for the coefficient, for the interaction between randomisation group and time-point in the mixed models. Secondary outcomes were analysed in the same manner except for the PGI-I, an ordinal outcome, which was analysed using ordinal regression. For all estimates, 95% confidence intervals were calculated and reported. Model checking consisted of visual inspection of residual plots and tests of proportionality for the ordinal model.

Two further pre-specified analyses were conducted for the primary clinical outcome, the first reflecting crossovers to the other trial group using a Complier Average Causal Effect model (instrumental variable two stage least squares method), and the second including only participants defined as ‘on treatment’ at the 18-month follow-up. In addition, the impact of missing primary clinical outcome data was assessed in two analyses: a complete case analysis, using only cases where 18-month PFIQ-7 was available (Missing Completely At Random); a pattern mixture model, increasing and decreasing the imputed PFIQ-7 values by the minimal clinically important difference of 20 points (Missing Not At Random), for participants in both groups and then in each group separately.^19^

Further planned sensitivity analyses of the primary clinical outcome measure were conducted: the inclusion of previous hysterectomy as a covariate; applying a repeated measures model with the baseline value in the outcome vector; and an alternative model to fit the distribution of the primary outcome data (zero inflated Poisson regression, added to the SAP on advice from the Data Monitoring and Ethics Committee based on observation of baseline PFIQ-7 responses). An analysis excluding participants whose care was disrupted by the Covid-19 pandemic was added post hoc.

Pre-specified subgroup analyses of the primary clinical outcome were carried out for age (<65/65+ years), pessary user type (new/existing) and previous hysterectomy (yes/no), as studies have indicated these factors may influence pessary management.^20^^,^^21^ A further post hoc subgroup analysis was conducted for use of local oestrogen at baseline. A stricter threshold for significance of p < 0.01 was set for these analyses.

A within-trial cost utility analysis was conducted according to a pre-specified Health Economics Analysis Plan. A health sector perspective was taken, with costs attached to resource use for the delivery of the self-management and clinic-based care interventions as well as all health care-related resource use for each participant during the follow-up period. Unit costs from the Personal Social Services Research Unit were attached to each item of resource. Outcomes were measured using the EQ-5D-5L and quality adjusted life years (QALYs) calculated from these data. The EQ-5D-5L utility score values were calculated following the approach recommended by the National Institute for Health and Care Excellence (NICE) using the cross walk from the UK EQ-5D-3L tariff.^22^ Incremental cost and QALYs were calculated for self-management compared to clinic-based care. The primary economic outcome was presented as an incremental net benefit (INB), calculated by multiplying incremental effectiveness by the UK policymaker cost-effectiveness threshold (£20,000 willingness-to-pay per QALY gained), and then subtracting the incremental cost of the treatment. A positive INB implies that self-management is a cost-effective option.

Analyses were conducted in Stata v16 (StataCorp LLC, College Station, TX). An independent Data Monitoring and Ethics Committee reviewed accumulating data and ethical issues. No interim analyses were planned or conducted. An independent Trial Steering Committee had oversight of the trial conduct. The trial was registered with ISRCTN (ISRCTN62510577) on 6 October 2017, prior to any participant recruitment.

### Ethics

The trial received ethical approval from the West of Scotland Research Ethics Service, West of Scotland REC 3 (17/WS/0267) on 17th February 2018. Participants were provided with a Participant Information Leaflet and gave written informed consent to take part.

### Role of the funding source

The funder of the study had no role in study design, data collection, data analysis, data interpretation, or writing of the manuscript.

## Results

**Fig. 1:**
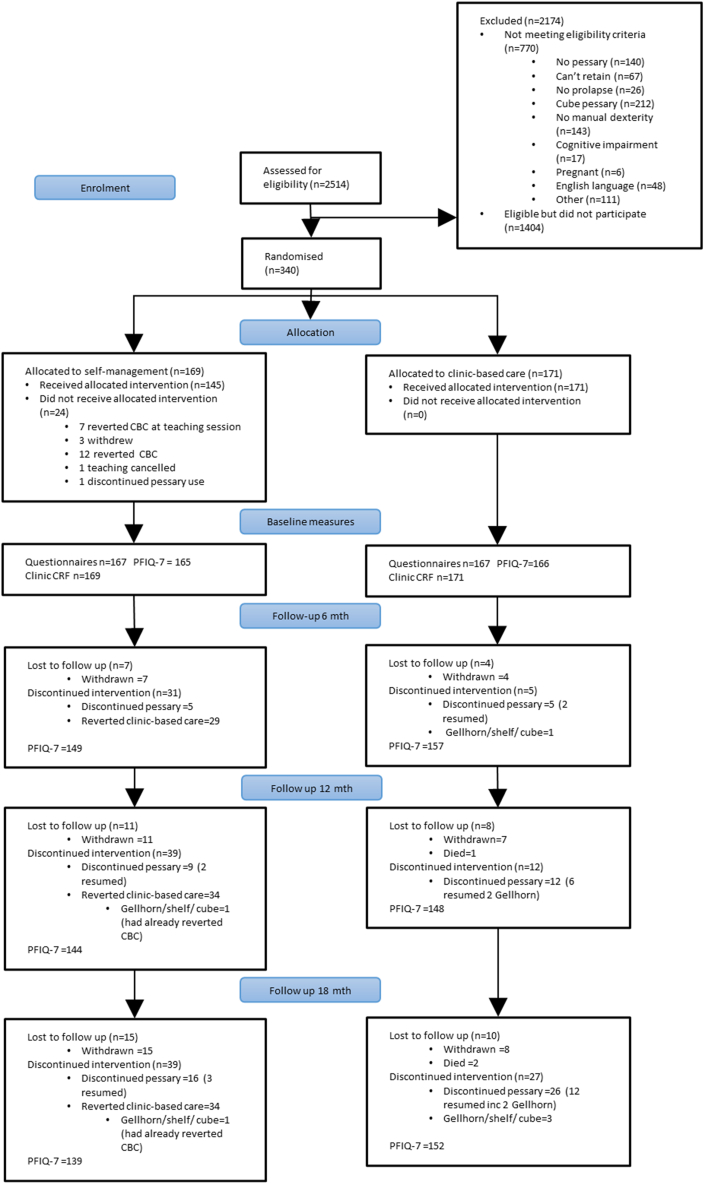
**Trial profile.** CBC = clinic-based care; CRF = case report form; PFIQ-7 = Pelvic Floor Impact Questionnaire short form 7. Note: “Discontinued intervention” categories are not mutually exclusive e.g., participants can both discontinue pessary use and revert to CBC; Participants may not have completed the PFIQ-7 but still completed other parts of the trial e.g., completed other outcome measures or attended 18-month clinic visit.

Between 16 May 2018 and 7 February 2020, 2514 women were screened for inclusion in the trial at 21 participating centres (Fig. 1) (Supplementary Tables S1 and S2). The most common reason for eligible women’s non-participation in the trial was a preference for their current treatment, and women who were randomised were younger and more likely to be a new user than those who were eligible but not randomised (Supplementary Table S3). Follow-up of participants was completed on 17 September 2021.

The recruitment target was met, with 340 women randomised (169 to self-management and 171 to clinic-based care) and primary outcome data available for 82.2% and 88.9%, respectively (Fig. 1). The main reason for non-response at follow-up was participants choosing to withdraw from the trial (n = 15 self-management and n = 8 clinic-based care) (Supplementary Table S4). Baseline characteristics of the randomised groups were similar (Table 1). Based on the question asked at each follow-up time-point “have YOU inserted your pessary in the last 6 months?”, at 18 months 141 (83.4%) and 103 (60.2%) of participants were “on treatment” in the self-management group (replied yes at one or more time-point) and clinic-based care groups (replied no or missing at all time-points), respectively. By 18 months, 34 (20.1%) participants had crossed-over from self-management to clinic-based care according to centre-completed “change of status” forms. No participant in the clinic-based care crossed over to self-management as none had received the trial self-management teaching. Of the 55 clinic-based care women who said they had removed their pessary at 18 months however, 27 (49%) reported they had received self-management training. Sixteen (9.4%) and 26 (15.2%) participants from self-management and clinic-based care, respectively had discontinued pessary use at 18 months.

**Table 1:** Characteristics of participants at baseline.

	Self-management N = 169		Clinic-based care N = 171		Total N = 340	
	N	Summary	N	Summary	N	Summary
Age						
Years–mean (SD)	169	63.2 (11.6)	171	64.2 (11.1)	340	63.7 (11.3)
Ethnicity–n (%)						
White	169	153 (90.5%)	171	156 (91.2%)	340	309 (90.9%)
Asian		3 (1.8)		4 (2.3)		7 (2.1)
Black		5 (3.0)		6 (3.5)		11 (3.2)
Mixed		1 (0.6)		0 (0)		1 (0.3)
Other		2 (1.2)		1 (0.6)		3 (0.9)
Missing		5 (3.0)		4 (2.3)		9 (2.7)
Number of births						
Births–mean (SD)	164	2.4 (1.1)	164	2.3 (1.2)	328	2.4 (1.1)
Median (IQR)		2 (2–3)		2 (2–3)		2 (2–3)
Body mass index						
Kg/m^2^–mean (SD)	165	26.1 (4.3)	162	26.6 (4.2)	327	26.3 (4.2)
Pessary user type						
New user—n (%)	169	53 (31.4%)	171	53 (31.0%)	340	106 (31.2%)
Systemic HRT						
Yes–n (%)	169	3 (1.8%)	171	10 (5.8%)	340	13 (3.8%)
Local oestrogen						
Yes–n (%)	169	47 (27.8%)	171	51 (29.8%)	340	98 (28.8%)
Hysterectomy						
Yes–n (%)	169	20 (11.8%)	171	18 (10.5%)	340	38 (11.2%)
Previous pelvic floor surgery						
Yes–n (%)	169	20 (11.8%)	171	19 (11.1%)	340	39 (11.5%)
Health of vaginal tissues						
Any of: ulceration/granulation/inflammation of tissues/other clinical concerns–n (%)	169	17 (10.1%)	171	20 (11.7%)	340	37 (10.9%)
Pessary material						
Latex–n (%)	167	1 (0.6%)	167	0	334	1 (0.3%)
Silicone–n (%)	167	54 (32.3%)	167	52 (31.1%)	334	106 (31.7%)
PVC–n (%)	167	90 (53.9%)	167	98 (58.7%)	334	188 (56.3%)
Vinyl–n (%)	167	22 (13.2%)	167	17 (10.2%)	334	39 (11.7%)
Pessary type						
Ring–n (%)	168	148 (88.1%)	171	151 (88.3%)	339	299 (88.2%)
Ring with support–n (%)	168	9 (5.4%)	171	11 (6.4%)	339	20 (5.9%)
Shaatz–n (%)	168	5 (3.0%)	171	6 (3.5%)	339	11 (3.2%)
Other–n (%)	168	6 (3.6%)	171	3 (1.8%)	339	9 (2.7%)
Pessary size						
mm–mean (SD)	168	71.0 (7.5)	170	70.7 (7.9)	338	70.9 (7.7)

Data are n/N (%) or mean (SD).

### Primary outcome

There were 291 out of a possible 340 (85.6%) 18-month questionnaires with valid primary clinical outcome data available for analysis: 139 (82.2%) in the self-management group and 152 (88.9%) in the clinic-based care group.

**Fig. 2:**
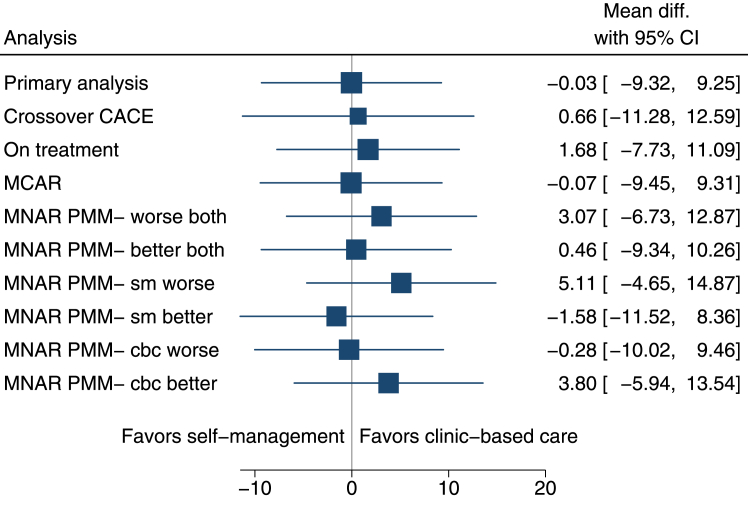
**Sensitivity analyses of treatment population definitions and missing data assumptions.** CACE = Complier Average Causal Effect model; MCAR = missing completely at random; MNAR = missing not at random; PMM = pattern mixture model, mean difference between trial groups in PFIQ-7 at 18 months with 95% confidence intervals.

The ITT analysis indicated there was not a statistically significant difference (self-management minus clinic-based care) between groups in PFIQ-7 at 18 months (adjusted mean difference −0.03 (−9.32 to 9.25)), or at 6 or 12 months (Table 2). The confidence intervals also rule out any clinical difference between groups, the smallest assumed meaningful difference being 20 points. Further pre-specified analysis of the primary outcome showed no significant difference between groups under different assumptions about the treatment population and missing data (Fig. 2).

**Table 2:** Results of primary outcome measure (PFIQ-7) intention-to-treat analysis by time-point.

	Self-management (SM)			Clinic-based care (CBC)			Unadjusted^a^ mean difference (SM-CBC) (95% CI)	Adjusted^b^ mean difference (SM-CB) (95% CI)	Effect size (adjusted estimate/pooled SD)
	N	Mean	SD	N	Mean	SD			
Baseline	165	32.5	49.6	166	31.7	48.0			
6 months	149	22.7	36.7	157	29.4	47.7	−6.71 (−16.31 to 2.89)	−5.90 (−15.00 to 3.20)	0.138
12 months	144	30.3	52.0	148	33.1	53.3	−2.78 (−14.90 to 9.35)	−3.45 (−12.71 to 5.82)	0.066
18 months	139	32.3	50.9	152	32.5	47.8	−0.17 (−11.55 to 11.22)	−0.03 (−9.32 to 9.25)	0.001

PFIQ-7 range from 0 to 300 with higher scores indicated poorer quality of life.

SM = self-management; CBC = clinic-based care.

a Unadjusted analysis included no random effects or covariates.

b Adjusted for age group, pessary user type (new vs existing) and baseline PFIQ-7 score and included random intercepts for participant and centre.

Similarly, planned sensitivity analyses found no statistically significant group difference in PFIQ-7 at 18 months with the following model adjustments: inclusion of previous hysterectomy as a covariate (−0.03 (95% CI −9.32 to 9.25)); applying the constrained longitudinal model with the baseline value in the outcome vector (95% CI −0.60 (−10.42 to 9.22)); zero inflated Poisson regression model for the 18-month PFIQ-7 (0.01 (95% CI −0.03 to 0.05)). Neither did a post-hoc analysis excluding 26 clinic-based care participants who had appointments cancelled due to the Covid-19 pandemic (−3.17, 95% CI −12.94 to 6.60).

**Fig. 3:**
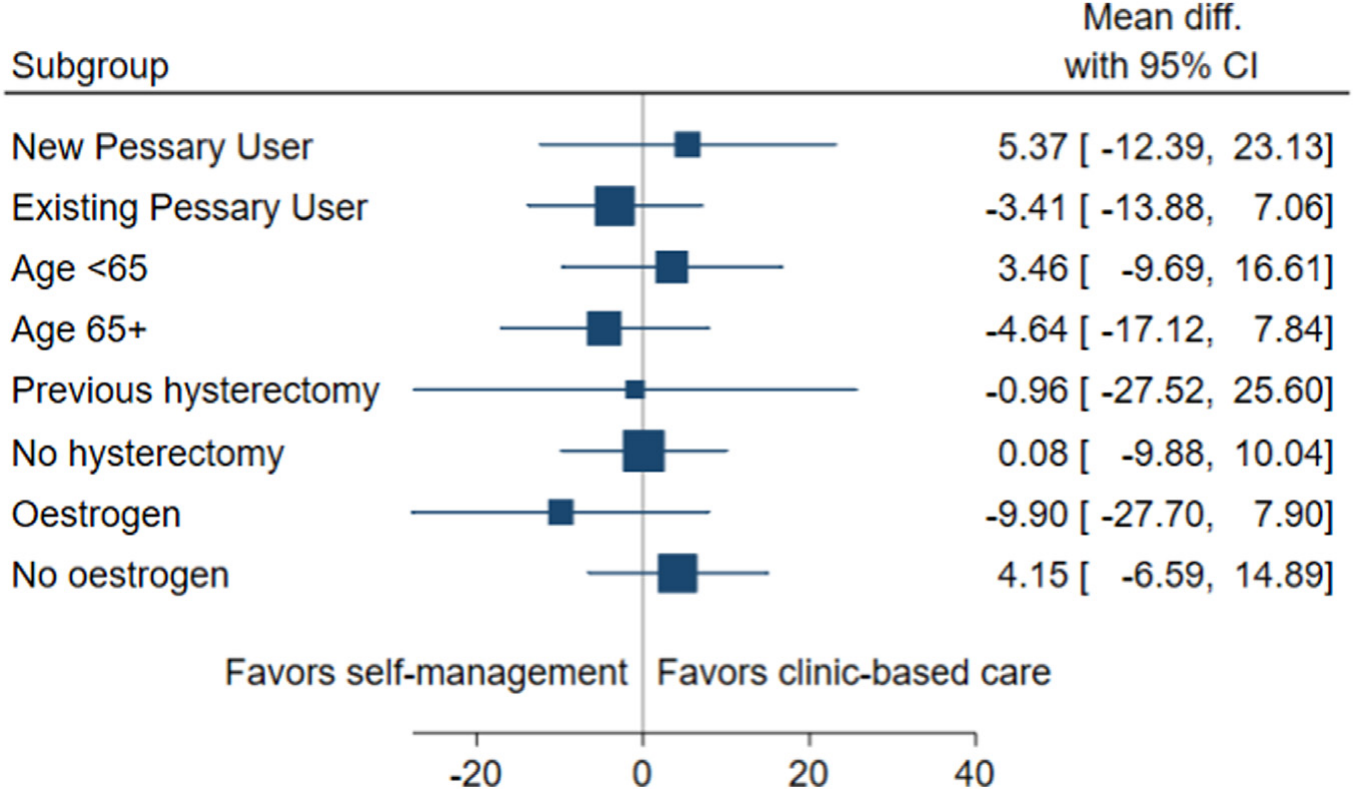
**Subgroup analyses.** Mean difference between trial groups in PFIQ-7 at 18 months with 95% confidence intervals.

Subgroup analysis of the primary outcome showed no significant effect of treatment group by subgroup interactions (subgroups were age <65 vs ≥65, p = 0.287, new vs existing pessary user p = 0.012, hysterectomy vs no hysterectomy at baseline p = 0.591 and local oestrogen vs no local oestrogen use at baseline p = 0.156) (Fig. 3).

### Secondary outcomes

Participants reported in the 18-month questionnaire whether they had experienced each of 15 categories of pessary-related complication, if applicable to them (Table 3). A lower percentage of pessary complications was reported in the self-management group (adjusted mean difference −3.83%, 95% CI –6.86% to −0.81%).

**Table 3:** Summary of participant-reported pessary-related complications at 18 months.

Complication reported	Self-management N = 142			Clinic-based care N = 152			Total N = 294		
	N	n	%	N	n	%	N	n	%
Vaginal discharge	141	41	29.1	151	49	32.5	292	90	30.8
Vaginal smell	141	26	18.4	150	33	22.0	291	59	20.3
Vaginal pain	141	11	7.8	147	17	11.6	288	28	9.7
Urine infection	141	17	12.1	151	16	10.6	292	33	11.3
Urine incontinence	140	71	50.7	152	79	52.0	292	150	51.4
Difficulty emptying bladder	141	25	17.7	147	41	27.9	288	66	22.9
Bowel incontinence	140	20	14.3	152	34	22.4	292	54	18.5
Difficulty emptying bowels	140	34	24.3	151	55	36.4	291	89	30.6
Unable to remove pessary	140	15^b^	10.7	150	11	7.3	290	26	9.0
Difficulty removing pessary	139	27^c^	19.4	149	12	8.1	288	39	13.5
Difficulty having sex	134	5	3.7	148	16	10.8	282	21	7.4
Pain during sex	136	4	2.9	147	9	6.1	283	13	4.6
Pessary fell out	138	6	4.3	145	19	13.1	283	25	8.8
Non-menstrual bleeding	138	15	10.9	150	22	14.7	288	37	12.8
Other	132	9	6.8	145	9	6.2	277	18	6.5
	N	Mean	SD	N	Mean	SD	N	Mean	SD

No. of complications reported as percentage of number relevant^a^	142	16.7	13.2	152	22.0	17.3	294	19.4	15.6

Data are n/N (%) or mean (SD).

a Percentage of complication types reported calculated for each participant; only 13 out of 15 categories applicable to both groups were included (2 pessary removal items excluded), and for participants who were not sexually active, 2 items relating to sex excluded.

b Of the 15 who were unable to remove their pessary, 11 had reverted to CBC.

c Of the 27 women who had difficulty removing their pessary 11 also reported not being able to remove their pessary.

At 18 months there was no significant difference between the groups in PFDI-20, PISQ-IR or General Self-Efficacy but women in the self-management group were more confident in their ability to manage pessary-related problems and to insert and remove their pessary (Table 4). Analysis of the PGI-I indicated that participants in the self-management group had significantly increased odds of perceiving an improvement in their pessary care: the proportion reporting their pessary care to be better was 9% higher than in the clinic-based care group (Table 4).

**Table 4:** Results of analysis of other secondary outcome measures at 18 months.

	Self-management		Clinic-based care		Adjusted mean difference (SM-CBC) between groups (95% CI)
	N	Mean (SD)	N	Mean (SD)	
PFDI-20	142	98.07 (47.13)	152	102.04 (52.05)	0.55 (−8.08–to 9.17)
General Self Efficacy	132	31.3 (5.4)	143	32.0 (4.0)	−0.77 (−1.69 to 0.14)
PISQ-IR^a^	67	2.9 (1.3)	73	2.8 (1.2)	0.34 (−7.19 to 9.87)
PISQ-IR NSA-condition specific	37	1.59 (0.77)	48	1.60 (0.65)	d
PISQ-IR NSA condition impact	44	1.88 (1.01)	50	1.97 (0.99)	d
PISQ-IR NSA global quality	28	2.67 (1.22)	39	2.63 (1.10)	d
PISQ-IR NSA partner related	41	2.37 (0.89)	47	2.69 (1.10)	d
Confident manage pessary	141	78.95 (26.03)	149	70.86 (28.10)	7.99 (1.82–14.15)
Confident insert pessary	142	81.28 (33.31)	152	48.36 (37.74)	32.78 (25.10–40.45)
Confident remove pessary	142	85.28 (30.24)	152	52.63 (38.63)	32.92 (25.19–40.64)
	N	n (%)	N	n (%)	Adjusted odds ratio (95% CI)

PGI-I–better	126	42 (33.3)	143	35 (24.5)	3.23 (1.47–7.13)^c^
Telephone support^b^	169	28 (16.6)	171	26 (15.2)	0.83 (0.45–1.55)

Data are n/N (%) or mean (SD).

PFDI-20 Pelvic Floor Distress Inventory-20.

SM = self-management; CBC = clinic-based care.

a PISQ-IR Pelvic Organ Prolapse/Urinary Incontinence Sexual Questionnaire IUGA Revised–only applicable to participants who are sexually active.

b From CRF completed by centre staff rather than participant questionnaires therefore N is higher. Binary response (any telephone support vs none) as only 5 participants received more than one support call.

c Odds ratio is from ordinal regression.

d Not sexually active (NSA) subscales of PISQ-IR, no planned comparison.

Of participants who answered questions about sexual activity at both baseline and 18 months in the self-management group, 51.1% (72/141) were not sexually active at baseline and of these 9 reported being sexually active at 18-month follow-up. In the clinic-based care group, 51.0% (77/151) reported not being sexually active at baseline and 4 of these women changed to reporting being sexually active at 18 months. At 18 months a higher percentage of the self-management group compared to clinic-based group participants intended to continue pessary use (130/138, 94.2% vs 131/148, 88.5%), and found pessary changes convenient (101/134, 75.4% vs 92/143, 64.3%), whilst similar percentages found pessary changes to be comfortable (64/135, 47.4% vs 76/143, 50.0%) and acceptable (122/135, 90.4% vs 131/146, 89.7%). Ulceration, granulation or other clinical concerns about the vaginal tissues (e.g., vaginal atrophy, erythema) on examination at 18 months were less common in the self-management group (27/152, 17.8% vs 43/160, 26.9%). Rates of serious adverse events were similar (17/169, 10.1% vs 14/171, 8.2%) and these were all evaluated as unrelated to participation in the trial. There were 323 reports of uptake of other prolapse-related healthcare (e.g., GP appointment, physiotherapy, dietetics) from 143 women in the self-management group over the 18-month follow-up (mean 2.3 per participant), compared to 428 reports from 154 women in the clinic-based care group (mean 2.8 per participant).

### Cost utility analysis

Health care resource use over the 18 months was summarised for each group (Table 5). Resource use by category and unit cost for each item is presented in Supplementary Table S5.

**Table 5:** Health care resource use in monetary terms by trial group over the 18-month follow-up.

	Self-management					Clinic-based care				
	n	Mean^a^ (GBP£, 2019 prices)	SD	Min	Max	n	Mean^a^ (GBP£, 2019 prices)	SD	Min	Max
Teaching appointment^b^	125	31.77	9.98	20	56.88	139	0	0	0	0
Clinic visits^c^		16.81	39.54	0	324.59		77.45	42.37	0	338.41
Telephone support^c^		1.45	3.51	0	17.09		1.76	4.07	0	18.85
NHS costs^d^		528.27	588.34	0	3743.29		649.63	654.02	0	3542.48
Medications^d^		15.52	45.57	0	348.00		24.90	79.88	0	667.88

a Mean calculations include zero reported resource use.

b Teaching appointment that applies only to self-management, costed as a 30-min appointment based on individual staff grades of first 156 appointments.

c From CRF data relates to follow-up appointments and calls for pessary-related treatment.

d Prolapse-related costs reported by participants including NHS appointments with a GP, practice nurse, district nurse, physiotherapist, dietician, outpatient consultant or nurse, accident and emergency and inpatient hospital episodes, and medications.

EQ-5D-5L responses were available for 264 participants at the 18-month time-point. No significant differences were found between the self-management and clinic-based care groups at any time-point (Table 6).

**Table 6:** EQ-5D-5L health state utility scores at baseline, 6 months, 12 months and 18 months follow-up, by randomised group.

Assessment/time-point	Self-management	Clinic-based care	Self-management	Clinic-based care	p-value^a^
	Mean (SD); n = 124^b^	Mean (SD); n = 140^b^	Median	Median	
**Index score**					
Baseline	0.851 (0.170); 125	0.840 (0.185); 139	1.000	1.000	0.732
6 months	0.841 (0.187); 125	0.829 (0.190); 139	0.814	0.814	0.593
12 months	0.833 (0.193); 125	0.811 (0.192); 139	0.814	0.814	0.301
18 months	0.823 (0.190); 125	0.819 (0.188); 139	0.814	0.814	0.856
**EQ-VAS**					
Baseline	83.28 (12.65); 125	82.40 (15.50); 139	85	85	0.912
6 months	80.83 (14.61); 125	80.39 (15.84); 139	85	85	0.903
12 months	79.59 (15.08); 125	79.50 (17.95); 139	80	80	0.524
18 months	78.56 (17.35); 125	79.15 (16.80); 139	80	81	0.608

a Mann–Whitney two sample test that data are from populations with the same distribution. A high p-value suggests no evidence of a difference between groups.

b Analysis sample.

The incremental cost and incremental effectiveness (QALYs) of self-management compared to clinic-based care were calculated along with the incremental cost-effectiveness ratio (ICER) and incremental net benefit (Table 7). The incremental net benefit was calculated by translating both effectiveness and cost into a monetary valuation using a willingness to pay for a QALY gained of £20,000. Self-management was less costly than clinic-based care and there was no difference in QALYs between the two groups. The positive incremental net benefit suggests self-management is cost-effective when compared to the clinic-based care.

**Table 7:** Cost effectiveness results for self-management and clinic-based care over the 18-month follow-up.

	Total cost (£GBP)	Total QALYs	Incremental cost	Incremental QALYs	ICER	Incremental net benefit (SE)
Self-management	£578.30	1.241	−150.53	0.021	Dominated	£564.32 (£581.50)
Clinic-based care	£728.84	1.221				

## Discussion

There was no evidence that pessary self-management was better or worse than clinic-based care in terms of the primary clinical outcome, women’s pelvic floor-specific quality of life. Self-management was less costly than clinic-based care, and this was driven by less resource use and health-seeking behaviour in the self-management group. Women who self-managed reported less pessary-related complications and greater confidence in managing pessary problems than women who received clinic-based care, and they were more likely to perceive an improvement in their pessary care.

Further analyses of the primary clinical outcome under differing assumptions relating to data distribution, non-compliance and missing data all led to the same conclusion as the intention-to-treat analysis, as did an analysis excluding data for women who had clinic-based care disrupted due to COVID-19. Pessary user type, age, hysterectomy and local oestrogen use status were investigated in subgroup analyses of the primary clinical outcome measure, however there were no significant differences in effect for any subgroup.

A key strength of this trial is its uniqueness in the field. There is no other randomised trial comparing self-management to clinic-based care for vaginal pessary.^2^^,^^23^

Women who were randomised were slightly younger (64 vs 67 years) and less likely to be an existing user (68% vs 80%) than those who were eligible but did not participate. This may mean the trial findings are less generalisable to older, existing pessary users. Although there were no significant subgroup effects relating to age or pessary user type, the direction of the effect favoured self-management for older and existing users which might suggest a differential effect of self-management on pelvic floor-specific quality of life in these groups. Thirty-nine percent declined randomisation because they had a treatment preference (33% CBC, 6% SM). Low participation rates and strong treatment preferences are seen in other conditions such as cancer.^24^ This will be explored further in the process evaluation analysis of interviews with randomised and non-randomised women.

Follow-up response rates were greater than 87% at all time-points. Although the 18-month response rate was higher for the clinic-based care group (88.9% vs 82.2%), it is reassuring that the investigation of data missing not at random concluded that this did not impact the primary outcome analysis conclusions. The core finding of no difference between groups was consistent across time and sensitivity analyses. The sample was drawn from geographically spread and diverse locations across the UK, however there was minimal ethnic diversity.

Thirty-four women (20.1%) crossed over from self-management to clinic-based care, including 11 women who had been unable to remove their pessary, and nearly 40% of those randomised to clinic-based care inserted their pessary themselves at least once over the 18-month follow-up. Both actions could potentially dilute the pelvic floor-specific quality of life effect of self-management in an intention-to-treat analysis, consequently leading to a loss of statistical power. However, this was a pragmatic trial, and cross-overs between treatments therefore occurred as they would in routine pessary management, and as such, this is an important part of the assessment of effectiveness. In addition, the on treatment analysis findings agreed with those of the ITT analysis.

There have been no previous randomised trials evaluating pessary self-management.^2^ Two previous small observational studies focused on pessary self-management. One offered a non-randomised comparison between self-management of vaginal pessary (n = 88) and clinic-based care (n = 46)^21^ but did not measure quality of life. The authors did report pessary changes being more comfortable and convenient for the self-management group. Similarly, self-managing women in this trial found pessary changes more convenient. The second study, a retrospective chart review of 289 women, again did not measure quality of life but identified self-management as a strong predictor of continuation.^25^ This is consistent with the current trial where the rate of pessary continuation was higher in the self-management group. Reviews of self-management interventions in other long-term conditions have found improvements in quality of life,^26^ whilst others have not,^27^ although the quality of evidence presented was low. This trial suggests a lack of association between self-management and quality of life in a previously unexplored population.

One possible explanation for the finding of no difference is that the trial compared two models of delivering the same intervention (self-management and clinic-based care for a vaginal pessary). Both groups of women used a pessary which is likely to have improved symptoms and quality of life, irrespective of the delivery model. More sensitive measures of quality of life factors that are important to women with pelvic floor dysfunction, other than symptoms, are being developed (https://www.nihr.ac.uk/documents/21583-patient-reported-outcome-measures-proms-for-pelvic-floor-disorders-commissioning-brief/29590) and will be useful in future pessary research. Generic quality of life as measured by the EQ-5D-5L also did not differ between groups, adding further evidence of no quality of life impact, positive or negative, of self-management in this population.

It was hypothesised that pessary self-management would increase women’s self-efficacy, leading to better quality of life. We found there was no difference between the trial groups in general self-efficacy, nor in quality of life. Women in the self-management group however did have greater self-efficacy specifically in relation to managing problems associated with their pessary, and, as expected had more confidence in their ability to remove and replace their pessary than women in the clinic-based care group who had not received the trial self-management teaching.

Despite the lack of impact on quality of life, women in the self-management group experienced proportionally fewer complications than women in the clinic-based care group: difficulties with emptying the bladder (17.7% vs 27.9%) and bowel (24.3% vs 36.4%) were more prevalent in the clinic-based care group, as were issues with vaginal tissues on examination at 18-month follow-up (17.8% vs 26.9%). This was unlikely to be due to differences in group characteristics, for example in pessary material or use of vaginal oestrogen, as groups were comparable at baseline. One small observational study of 100 women with prolapse identified self-management as a means of reducing adverse events, with 16% of self-managing women experiencing adverse events compared to 62% of non-self-managing women^28^; and another audit of 75 self-managing women noted a low complication rate of 11.8%.^29^

Fewer complications in the self-management group may be explained by these women having greater confidence in their abilities to remove and insert their pessary, to change it more frequently and use it for less time. One small randomised study identified that the rate of vaginal pessary complications was lower when pessary changes were more frequent, although not statistically so.^30^ Complications have also been linked to the duration of time the pessary is in situ.^8^

Previous observational, longitudinal studies have found that 86.1% of women continue pessary use at 5 years^31^ and a link between complications and pessary discontinuation has been reported.^25^ This is in line with the current trial as 91.5% and 86.2% of self-management and clinic-based care women, respectively intended to continue pessary use after 18-month follow-up.

The UK Clinical Guideline for best practice in vaginal pessary use for prolapse recommends that women who are assessed to be willing and suitable should be offered self-management.^32^ This trial supports this recommendation to clinicians and policymakers, providing evidence that self-management does not negatively impact quality of life or patient safety compared to clinic-based care, but is cost-effective and associated with a reduced rate of complications.

Further research is needed to understand the mechanisms through which self-management reduces complication rates, and whether it leads to a reduction in pessary discontinuation. Research on models of pessary care in ethnic minority populations is also needed. A validated pessary complications questionnaire needs to be developed to enhance the rigor and consistency of future trials of pessaries. Future research is needed to focus on models of pessary self-management follow-up, for example, should a pessary review be woman-initiated or does it require to be planned at specific intervals? A 4-year follow-up is underway in this trial, and an implementation study planned, which will contribute data to address some of these gaps.

## Contributors

All authors were involved in reading, commenting upon, and taking the decision to submit the manuscript for publication. All authors had access to the data analysis output and SH, CB and AE directly accessed and verified the underlying data reported in the manuscript. Additional contributions were as follows:

SH: Had responsibility overall for the manuscript, and for delivery of the trial. She was co-Chief investigator and was involved in early conception of the trial and made intellectual input to the study design.

RK: Had responsibility overall for the clinical aspects of the trial delivery. She was co-Chief investigator and was involved in early conception of the trial and made intellectual input to the study design.

KG: Was Trial Manager, responsible for the day-to-day operationalisation and management of the trial.

CB: Was responsible for preparing the statistical analysis plan, delivery and reporting of the statistical analysis.

AE: Was responsible for supervising the conduct of the trial data analysis.

LM: Was responsible for trial data management.

LD: Was involved in early conception of the trial, contributed to developing the trial interventions, delivered training and on-going clinical support to clinical staff at recruiting centres.

MD: Was responsible for delivery and reporting of the process evaluation component of the trial.

MG: Provided advice from the perspective of a service user, preparing the protocol and contributing to the Project Management Group.

WA: Contributed expertise in clinical trials and pelvic floor dysfunction and was a local Principal Investigator.

SB: Provided senior trial management expertise and support for the trial.

JC: Provided advice from the perspective of a service user, preparing the protocol and contributing to the Project Management Group.

AF: Provided advice from the perspective of a service user, preparing the protocol and contributing to the Project Management Group.

MF: Developed and provided on-going support for the trial database.

KG: Contributed expertise in clinical trials and pelvic floor dysfunction and was a local Principal Investigator.

CH: Contributed expertise in clinical trials and pelvic floor dysfunction and was a local Principal Investigator.

AK: Contributed expertise in clinical trials and pelvic floor dysfunction and was a local Principal Investigator.

SM: Conducted the health economics analysis.

HM: Was responsible for delivery, supervision and reporting of the health economics analysis.

DM: Was involved in early conception of the trial, made intellectual input to the study design.

JN: Contributed expertise in complex intervention trials methodology.

RT: Contributed expertise in clinical trials and pelvic floor dysfunction and was a local Principal Investigator.

CBu: Was Chief Investigator for the entire research, led the conceptualisation of the trial and parallel process evaluation, and was responsible for delivery and reporting of the qualitative components of the study.

## Data sharing statement

Individual participant data collected for this trial, and a data dictionary defining each field in the dataset, will be made available to others; all available data will be de-identified participant data. To access data, a request should be submitted to the corresponding author with a scientific proposal including objectives. The Chief Investigator, in collaboration with the sponsor, will assess all requests for data sharing. Consent from participants has been obtained to share their anonymised data with other researchers to support future research.

## Declaration of interests

All authors declare a grant from National Institute for Health and Care Research (NIHR) Health Technology Assessment (HTA) programme relating to the research described in the manuscript. In addition **Lucy Dwyer** reports being a member of the NICE guideline committee for non-surgical management and prevention of pelvic floor dysfunction and a member of the UK Clinical Guideline for best practice in the use of vaginal pessaries for pelvic organ prolapse committee; **Aethele Khunda** reports receiving an educational grant from Olympus Medical Systems to cover travel and accommodation costs to attend a laparoscopic urogynaecology workshop; **Wael Agur** reports grants from NIHR, consulting fees and payment for testimony from Oaklaw Consultancy Ltd., for Medico-legal Consultancy and financial/non-financial interest associated with Medical Innovation Systems; **Karen Guerrero** reports payment for expert testimony for the Medical Advisor NHS Scotland Central Legal Office and for NHS-funded study leave; **Christine Hemming** reports grants from NIHR HTA for two RCTs (11/129/183 and 07/60/18); **John Norrie** reports being a member of the following committees: NIHR CTU Standing Advisory Committee (2018-23), NIHR HTA & EME Editorial Board (2015–2019), EME Funding Committee Member (2019–2022), HTA General Committee (2016–2019), HTA Post-Funding Committee (2016–2019), HTA Funding Committee Policy Group (2016–2019), COVID-19 Reviewing (2020); **Ranee Thakar** reports a voluntary role at Royal College of Obstetricians and Gynaecologists; **Rohna Kearney** reports being topic lead for prolapse on NICE guideline NG123 published 2019; **Angela Forrest** reports payment for attending project meetings relating to the research; **Suzanne Breeman** reports grants from NIHR HTA for the VUE trial (11/129/183) and the PROSPECT trial (07/60/18 and NIHR133665). **Suzanne Hagen, Carol Bugge, Andrew Elders, Helen Mason, Kirsteen Goodman, Doreen McClurg, Melanie Dembinsky, Lynn Melone, Catherine Best, Sarkis Manoukian, Margaret Graham, Jane Culverhouse and Mark Forrest** have no additional disclosures to report relating to the manuscript.

## Appendix A Supplementary data

Supplementary Tables

TOPSY FINAL Protocol V11 08.11.2022
