# Supplementary material for: Clinical effectiveness of vaginal pessary self-management vs clinic-based care for pelvic organ prolapse (TOPSY): a randomised controlled superiority trial
Source: eClinicalMedicine. 2023 Nov 23;66:102326. doi: 10.1016/j.eclinm.2023.102326 (PMC10701109; doi:10.1016/j.eclinm.2023.102326)
Supplement: Supplementary Tables [file mmc1.docx]

**Supplementary tables**

**Table I. Reason for non-inclusion of eligible women**

| **Reason** | **n (%)** |
| --- | --- |
| Missed by centre | 155 (11·04%) |
| Declined without reason | 110 (7·83%) |
| Did not want to be randomised | 123 (8·76%) |
| Had a preference for clinic-based care | 459 (32·69%) |
| Had a preference for self-management | 89 (6·34%) |
| Wished surgery | 47 (3·35%) |
| Declined with reason (excluding preference for trial group or surgery) | 69 (4·91%) |
| Other/missing | 352 (25·07%) |
| Total | 1404 (100%) |

**Table II. Recruitment by centre**

| **Centre Name** | **No. Randomised** |
| --- | --- |
| Ayrshire and Arran (Ayr Hospital) | 17 |
| Aberdeen | 10 |
| Croydon | 33 |
| Manchester (St.Marys) | 50 |
| Middlesbrough | 57 |
| Glasgow | 16 |
| Kings College London | 11 |
| Basingstoke | 14 |
| Addenbrookes Hospital | 11 |
| Sheffield | 17 |
| Birmingham | 23 |
| Liverpool | 7 |
| NHS Lothian | 11 |
| Dunfermline | 16 |
| County Durham and Darlington | 4 |
| Plymouth | 4 |
| Yeovil | 6 |
| Taunton and Somerset (Musgrove Park Hospital) | 9 |
| Newcastle | 9 |
| NHS Lanarkshire | 4 |
| Norwich | 11 |
| Total | 340 |

**Table III. Comparison of characteristics of eligible women who did and did not participate**

|  | **Eligible but not included** | **Randomised** | **Test, p value** |
| --- | --- | --- | --- |
| Age | 67·1 (13·1) n=1257 | 64·1 (11·4) n=310 | t = -3·659  P<0·001 |
| Existing users | 1015/1268 (80·1%) | 213/312 (68·3%) | Chi^2^(1)=20·06 p<0·001 |

**Table IV. Reasons for loss to follow-up at 18 months**

|  | Self-management  group | Clinic-based care  group |
| --- | --- | --- |
| Died |  | 2 |
|  |  |  |
| Chose to withdraw from trial because: |  |  |
| Reverted to clinic-based care | 6 |  |
| Did not feel they could self-manage | 4 |  |
| Had surgery | 2 | 2 |
| Wanted surgery | 1 |  |
| No longer interested in trial | 1 |  |
| Discontinued pessary | 1 | 4 |
| Struggled with paperwork |  | 1 |
| Moved away |  | 1 |
| Total | 15 | 10 |

**Table V. Healthcare Resource use by randomised group over the 18-month follow-up period for prolapse and other health reasons**

|  | **Self-Management Group** | | **Clinic-based Care Group** | |  |  |
| --- | --- | --- | --- | --- | --- | --- |
|  | **Prolapse related appointment** | **Other health reason** | **Prolapse related appointment** | **Other health reason** | **Total Self-Management** | **Total clinic-based Care** |
| N* |  | | | | 158 | 152 |
| Clinic Appointment for pessary fitting and check up | N/A | N/A | N/A | N/A | 91 | 333 |
| Telephone Support Calls | N/A | N/A | N/A | N/A | 30 | 29 |
| GP surgery appointment | 37 | 319 | 71 | 424 | 356 | 495 |
| Nurse surgery appointment | 17 | 189 | 16 | 213 | 206 | 229 |
| GP home visit | 0 | 5 | 1 | 6 | 5 | 7 |
| Nurse home visit | 0 | 4 | 1 | 12 | 4 | 13 |
| District nurse home visit | 31 | 21 | 1 | 6 | 52 | 27 |
| Physiotherapy | 53 | 33 | 34 | 89 | 86 | 123 |
| Clinic dietitian | 0 | 0 | 0 | 11 | 0 | 11 |
| Outpatient doctor | 70 | 139 | 112 | 207 | 209 | 319 |
| Outpatient nurse | 110 | 87 | 180 | 94 | 197 | 274 |
| Accident and Emergency visits | 5 | 53 | 12 | 60 | 58 | 72 |
| Hospital bed-days** | 24*** | 47 | 5 | 99 | 71 | 104 |
| Hospital episodes |  | | | | 15 | 15 |
| *Sample at 6 month follow-up. Changes to self-management 146, clinic based 152 at 12 months and self-management 143, clinic based 154 patients at 18 months.  ** Reported without adjustments for known issues for example some patients reported stay for knee replacement as prolapse.  ***Only 6 confirmed as prolapse related. | | | | | | |
