# Supplementary material for: Clinical effectiveness of vaginal pessary self-management vs clinic-based care for pelvic organ prolapse (TOPSY): a randomised controlled superiority trial
Source: eClinicalMedicine. 2023 Nov 23;66:102326. doi: 10.1016/j.eclinm.2023.102326 (PMC10701109; doi:10.1016/j.eclinm.2023.102326)
Supplement: TOPSY FINAL Protocol V11 08.11.2022 [file mmc2.pdf]

A multicentre randomised controlled trial, with nested process evaluation, to test the clinical and cost-effectiveness of self-management of vaginal pessaries to treat pelvic organ prolapse, compared to standard care to improve women's quality of life.

(**TOPSY TRIAL** – **T**reatment **O**f **P**rolapse with **S**elf-care pessar**Y**).

PROTOCOL VERSION 11
08th November 2022

CI: Dr Carol Bugge
Sponsor: Glasgow Caledonian University

A UK Collaborative Study funded by the
NIHR Evaluation, Trials and Studies Coordinating Centre (NETSCC),
Health Technology Assessment (HTA) Programme (16/82/01)

TOPSY Protocol Version Log

Version	Version Date	Significant Changes
1.0	29th November 2017	Protocol submitted to ethics for original application
2.0	08th February 2018	Protocol updated due to provisional opinion letter from ethics. This version of the protocol was used at the start of the pilot centre set up.
3.0	27th June 2018	Minor clarifications in the protocol. Two clarifications on timings were added to the protocol so that centres were clear about acceptable timelines between screening and randomisation and randomisation and the teaching appointment. *Randomisation should occur within **4 weeks** of assessing eligibility (page 21)* *The teaching appointment should take place within **4 weeks** of the randomisation date (page 27).* *The wording was changed slightly in consent pathway B on page 60. No processes were changed just a clarification*
4.0	17th January 2019	Update on the way centres can recruit women (additional phone call after clinical contact or after non response to postal pack) and outcome table on page 30.
5.0	25th July 2019	Clarifications on timings and Section 9.9
6.0	22nd July 2020	An additional questionnaire to capture the impact of COVID-19 on participants' views on pessary care/ self-management. Addition of COVID 19 protocol annex
7.0	23rd November 2020	Updated project timelines for 6 month extension
8.0	11h March 2022	Addition of Appendix D, protocol Annex for 4 year follow up
9.0	18TH May 2022	Change of Sponsor
10.0	02nd August 2022	Update on funder contact details (P57/58); addition of SWAT 86 to Appendix D (Participant Selection and Enrolment); update on adverse and serious adverse event report to Appendix D; and addition of Appendix E (SWAT 86 Protocol)
11.0	08th November 2022	Addition of Appendix F, protocol annex for APPLE (sub-study of TOPSY)

TOPSY PROTOCOL APPROVAL

Treatment **O**f **P**rolapse with **S**elf-care pessar**Y**

The undersigned confirm that the following protocol has been agreed and accepted and that the Chief Investigator (CI) agrees to conduct the trial in compliance with all regulatory requirements.

Signatures

Dr Carol Bugge<br>**Chief Investigator**	____________________<br>Signature	____________<br>Date
Professor Suzanne Hagen<br>**Co -Chief Investigator**	____________________<br>Signature	____________<br>Date
Dr Rohna Kearney<br>**Co- Chief Investigato**r	____________________<br>Signature	____________<br>Date
Dr Kirsteen Goodman<br>**Trial Manager**	____________________<br>Signature	____________<br>Date
Mr Andrew Elders<br>**Trial Statistician**	____________________<br>Signature	____________<br>Date

Each Principal Investigator of each centre will sign and date a protocol declaration to confirm they agree and have accepted the current protocol.

CONTENTS

TOPSY protocol approval ... 3
PROTOCOL SUMMARY TABLE ... 6
QUESTIONS ADDRESSED ... 6
PROTOCOL SUMMARY IN PLAIN ENGLISH ... 8
Glossary of abbreviations ... 9
TOPSY Study FLOW DIAGRAM ... 10
1. BACKGROUND AND RATIONALE ... 11
2. STUDY RESEARCH QUESTIONS AND OBJECTIVES ... 13
2.1 Research Questions ... 13
2.1 Objectives ... 13
3. STUDY DESIGN ... 14
3.1 Internal Pilot ... 14
3.2 Process Evaluation ... 16
3.3 Cost-effectiveness analysis ... 16
4. STUDY POPULATION ... 17
4.1 Sample Size Calculation ... 17
4.2 Inclusion Criteria ... 18
4.3 Exclusion Criteria ... 18
4.4 Summary of the sample ... 18
5 PARTICIPANT SELECTION AND ENROLMENT ... 19
5.1 Identifying and consenting women and Health Care Professionals ... 19
5.1.3 Recruitment and consent to audio-record recruitment sessions (n=12-18, pilot study) ... 21
5.1.5 Recruitment and Consent for interview study with randomised women (n=36) ... 23
5.1.6 Recruitment and consent for interview study with non-randomised women (n=20) ... 23
5.1.7 Recruitment and consent for healthcare professional interviews ... 24
5.2 Randomisation and web-based data management system ... 25
5.3 Consent and withdrawal of Study Participants ... 25
5.4 Retention methods ... 26
6 INTERVENTION ... 27
6.1 Pessary self-management ... 27
6.2 Standard pessary care ... 29
7. DATA COLLECTION ... 30
7.1 Data collection for main trial ... 30
7.1.1 Primary outcome ... 31
7.1.2 Validated secondary outcome measures ... 31
7.1.3 Non-Validated secondary outcome measures ... 32

7.1. 4 COVID-19 Survey 34
7.2 Data collection for Process Evaluation 34
7.3 Data collection for Economic Evaluation 36
8 PROPOSED ANALYSES 38
8.1 Quantitative data analysis 38
8.2 Process Evaluation Data Analysis 39
8.3 Economic Analysis 41
9 ORGANISATION - Trial coordination 42
9.1 Sponsor 42
9.2 Clinical Trials Unit 42
9.3 Study offices 43
9.4 Trial centres 43
9.5 TSC and DMEC 44
9.6 Milestones 44
9.7 Finance 46
9.8 Confidentiality 46
9.9 Data Protection 46
10. SAFETY REPORTING 47
10.1 Procedure for reporting AEs and SAEs in TOPSY 47
10.1.1 Detecting AEs, ARs SAEs and SARs 49
10.1.2 Recording AEs and SAEs 49
10.1.3 Assessment of AEs and SAEs 50
10.2 Reporting responsibilities of the local PI and CI 50
11. END OF STUDY 50
12. CONTINUATION OF INTERVENTION FOLLOWING THE END OF STUDY 51
13. INDEMNITY 51
14. PUBLICATION AND DISSEMINATION 51
15. REFERENCES 53
APPENDIX A: TOPSY Contact Information and Oversight groups 57
APPENDIX B: The TOPSY study consent pathways. 59
APPENDIX C- TOPSY COVID-19 Protocol Annex 65
APPENDIX D - TOPSY 4 year follow up Protocol Annex 69
APPENDIX E - SWAT 86 Protocol........ 75
APPENDIX F - The APPLE sub-study........78

PROTOCOL SUMMARY TABLE

**QUESTIONS ADDRESSED**	1. What is the clinical and cost-effectiveness of self-management of vaginal pessaries to treat pelvic organ prolapse, compared to standard pessary care, on condition-specific quality of life?<br>2. What are the barriers and facilitators to intervention acceptability, intervention effectiveness, fidelity to delivery, and adherence for women treated with vaginal pessary and the healthcare professionals who treat them, and how does this differ between randomised groups?
**TRIAL PARTICIPANTS**	Woman 18 years or older with prolapse of any type or severity, who have successfully used a pessary for at least two weeks.
**TRIAL ENTRY**	Consent will be obtained from eligible women after written and verbal information has been provided.
**INTERVENTIONS**	1. Women in the standard care group will be seen at regular intervals (as per local centre protocol) to have their pessary removed and a new one inserted.<br>2. Women in the self-management group will have a 30 minute teaching appointment with a healthcare professional trained in delivery of the self-management intervention who will teach women to remove, clean and re-insert their pessary. Women will receive a follow up phone call and a phone number to call if problems are experienced.
**OUTCOMES**	Primary outcome measure is prolapse-specific quality of life (PFIQ-7).<br><br>Secondary outcomes/Questionnaires;<br>• Generic quality of life (EQ-5D-5L)<br>• Prolapse symptoms (PFDI-20);<br>• Sexual dysfunction (PISQ-IR);<br>• Self-efficacy (GSE)<br>• Pessary Use Questionnaire (Patterns of pessary use, acceptability and benefit)<br>• Pessary complications<br>• Pessary Confidence Questionnaire (Efficacy)<br>• Adherence to trial group; crossover to other group.<br>• Resource Use Questionnaire (for health economic evaluation)
**CO-ORDINATION**	Local: by local lead Principal Investigator, an Intervention healthcare professional and a Recruitment Officer.<br>Central: by TOPSY Study Glasgow Office in Glasgow Caledonian University.<br>Overall: by the Project Management Group and overseen by the Trial Steering Committee and the Data Monitoring and Ethics Committee.

**FUNDING**	National Institute for Health Research Evaluation, Trials and Studies Coordinating Centre, Health Technology Assessment (NETSCC HTA) Programme
**Start date:**	01st November 2017 (data collection start earliest 01.04.18)
**Planned finish date:**	31st Jan 2021 (all participant follow ups completed)
**Planned reporting date:**	31st March 2022
4 year follow-up start date:	01st February 2022
4 year follow-up finish date:	31st January 2024

PROTOCOL SUMMARY IN PLAIN ENGLISH

Pelvic organ prolapse (or prolapse) is a common condition in women where the pelvic organs (bladder, bowel or womb) descend into the vagina and cause distressing symptoms that adversely affect quality of life. Two thirds of women will opt to try a vaginal pessary to treat their prolapse symptoms when it is offered. The pessary is inserted into the vagina and helps to support the pelvic organs. It is usually fitted at a gynaecological clinic and the woman returns approximately every six months to have it removed and changed.

However, it is possible that women could remove, clean and re-insert their pessary themselves at home; this is called self-management. The evidence suggests that self- management is effective because people become more confident in their ability to look after their own health.

This study aims to assess if self-management of a vaginal pessary is more effective at improving the quality of life of women with prolapse than standard follow up care (visiting the clinic for regular follow-up care).

Women with any severity or kind of prolapse will be invited to take part in the study as long as they have successfully used a pessary for at least two weeks. They will receive written information about the study and, if eligible and willing to take part, they will sign a consent form and then be assigned at random to either self-management or standard care. Women in the standard care group will be seen approximately every four to six months in a hospital or community clinic for the pessary to be removed and new one inserted. Woman in the self-management group will have a 30-minute appointment with a specialist nurse or physiotherapist to be shown how to remove and change their pessary, and to allow them time to practise this. Woman will be encouraged to try changing and cleaning their pessary within two weeks of the appointment and they will be telephoned to find out if they have been able to do so successfully. Women in the self-management group will be offered a centre specific phone number to call if they have any problems with their pessary.

To find out if self-management does improve women's quality of life, all participants will be asked to fill out questionnaires at the start of the study and six,12 and 18 months later. We will collect information to determine if self-management is more or less expensive for the NHS and for women. To help understand how and why self-management may or may not work we will record some of the appointments where self-management is taught and we will interview a sample of women and healthcare professionals taking part in the study.

Glossary of abbreviations

TOPSY	**T**reatment **O**f **P**rolapse with **S**elf-care pessar**Y**
CI	Chief Investigator
CRF	Case Report Form
DMEC	Data Monitoring and Ethics Committee
HTA	Health Technology Assessment
ISF	Investigator Site File
ISRCTN	International Standard Randomised Controlled Trial Number
ITT	Intention to treat
NETSCC	NIHR Evaluation, Trials and Studies Coordinating Centre
NIHR	National Institute for Health Research
PFDI	Pelvic Floor Distress Inventory
PFIQ	Pelvic Floor Impact Questionnaire
PI	Principal Investigator
PISQ	Prolapse/Incontinence Sexual Questionnaire
PMG	Project Management Group
QALY	Quality Adjusted Life Year
REC	Research Ethics Committee
SAE	Serious Adverse Event
SOP	Standard Operating Procedure
TSC	Trial Steering Committee

TOPSY Study FLOW DIAGRAM

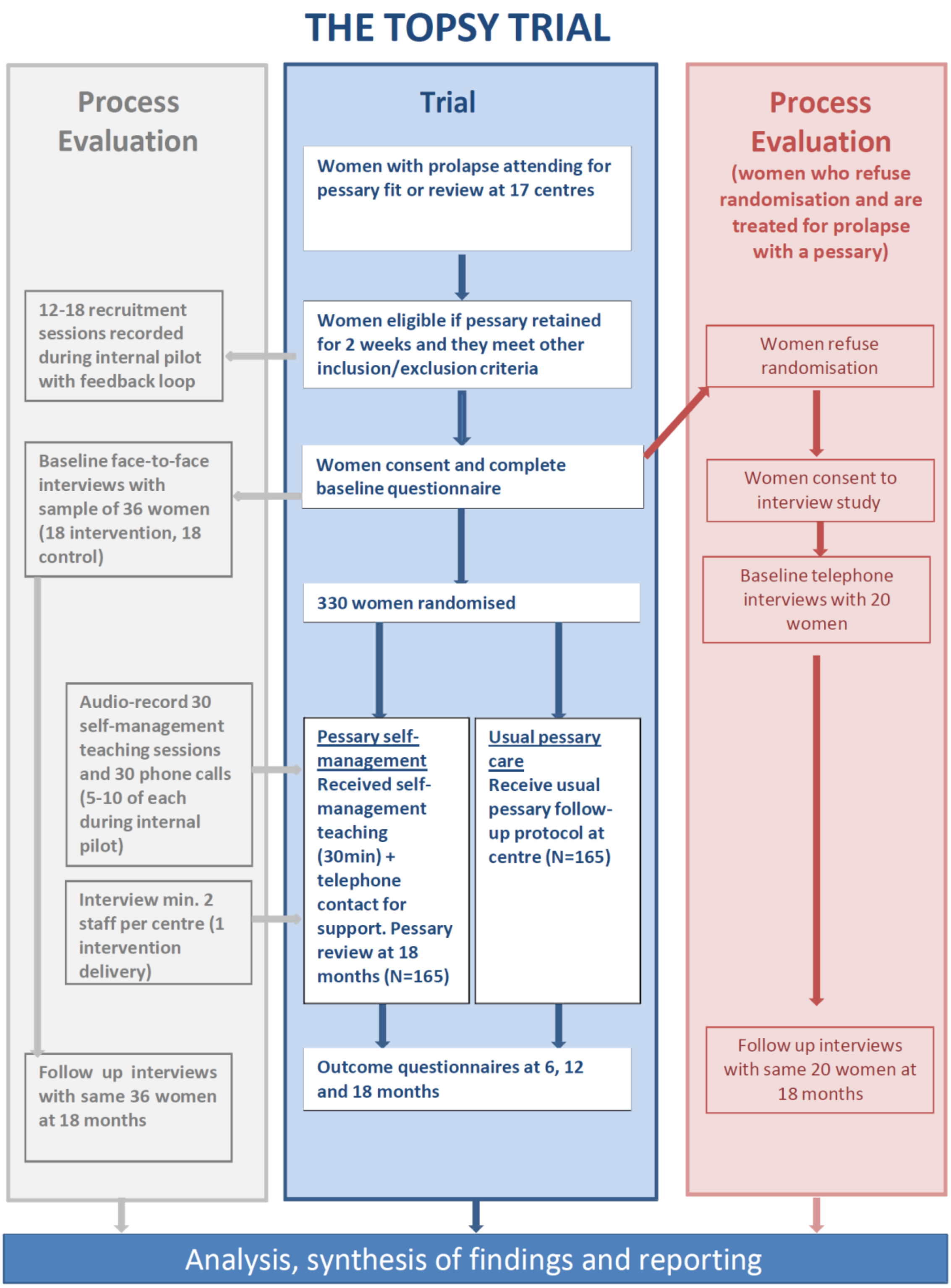

1. BACKGROUND AND RATIONALE

Pelvic organ prolapse affects about 40% of women over 40 years of age (Hendrix et al., 2002) and the numbers of women affected is expected to rise (Wu et al., 2011). Prolapse is categorised into different stages and types and affects women of varying ages. The distressing symptoms include a sensation of "something coming down" in the vagina, urinary, bowel and sexual problems and pelvic and back pain. These symptoms impact negatively on a woman's quality of life (Jelovsek and Barber, 2006).

Women presenting with prolapse are most commonly offered the option of conservative management (such as a vaginal pessary) or surgery. About 9.5% of women will undergo surgery for prolapse in their lifetime (Abdel-Fattah et al., 2011). Over 29,000 prolapse repairs were performed in England in 2012/13 costing over £60 million (NHS Information centre, 2013). However, surgery is not always effective or durable with 30% of women requiring at least one further procedure (Olsen et al., 1997). With the high re-operation rates and the controversy surrounding surgery and the use of mesh implants, it is timely to consider the evidence supporting conservative options in more detail.

Currently women who have prolapse of all types and stages can receive pessary treatment (although pessary use is uncommon in women with stage I prolapse). Most commonly women who use a pessary are over 60 years of age (Bugge et al., 2013) and two thirds of women will opt to try a pessary when offered (Kapoor et al., 2009). Although previous research indicates that the ring pessary is most commonly used in practice, a wide range of pessaries are available and are used (Kapoor et al., 2009). In scoping pessary provision for a previous study it was found that typically less than ten pessaries a year are fitted in an average primary care practice. Hospital-based care remains the most common delivery setting for pessaries with community-based clinics also offering services. Thus the most common service model for women is to return to clinic to have their pessary removed and changed (Bugge et al., 2013). Most commonly, women attend a clinic every six months for a pessary change, but time between changes does vary (3-12 months) (Gorti et al., 2009; Bugge et al., 2013). It is not clear if pessaries would be used more often if pessary care was less reliant on follow-up clinic appointments, allowing easier integration with a woman's lifestyle.

The largest UK-based study reported that 86% of women successfully retaining a pessary at four weeks, will continue to use a pessary at five years (Lone at al., 2011). However other studies have reported much lower continuation rates (Sarma et al., 2009; Ramsay et al., 2016). Reasons for

discontinuation of pessary use include developing complications such as bleeding or infection, dislike of the pessary changing procedure and inconvenience of attending appointments (Gorti et al., 2009).

A UK multi-professional survey undertaken by members of the research team in 2013 found that only 17% of clinicians offered women the option of self-managing their pessary (Bugge et al., 2013). This is a significant difference in practice compared with North America, where the majority of clinicians teach women pessary self-care (Hanson et al., 2006). The ongoing Cochrane review update has identified seven completed and five ongoing trials evaluating various aspects of pessary use. None of these trials tested self-management for pessary in any comparison.

Self-management focusses on actions that people undertake for themselves to manage their health and illness. In order to self-manage people need self-management support (actions taken to support people to self-manage e.g. by healthcare professionals). Self-management has been shown to be effective in improving health outcomes such as quality of life; e.g. condition-specific quality of life is improved for people with Chronic Obstructive Pulmonary Disease (Zwerink et al., 2014).

There is only one small (n=88) non-randomised study, undertaken by a member of the research team, that assesses self-management of vaginal pessaries, which reported gains from self-management, in that women reported higher levels of convenience, ability to access help, support and comfort than those having clinic management (Kearney and Brown, 2014). Women who were self-managing had one clinic appointment scheduled at two years, compared to standard care where women attended every four to six months for pessary changes. Whilst these may be promising findings there is an urgent need to robustly investigate whether pessary self-management is more clinically and cost-effective than standard pessary care.

Self-management interventions are highly heterogeneous (Lorig and Holman, 2003; Ryan and Sawin, 2009; Zwerink et al., 2014), making identification of the effective component parts of an intervention difficult. However, based on evidence drawn from large scale self-management programmes, three tasks need to be achieved in order for individuals to self-manage (Lorig and Holman, 2003): medical management of the condition; role management and emotional management. This study aims to intervene at various levels (service level, professional level and individual woman level) to ensure the woman can achieve the necessary tasks to self-manage.

There is an anticipated rise in the prevalence of prolapse with an ageing population (Wu et al., 2011).

In 2014 Dr Foster reported that there were 52,567 outpatient hospital appointments in England for pessary care. The impact that self-management may have on the current and future NHS workload, as well as women's quality of life, needs to be evaluated.

Previous pessary trials, where women are randomised prior to pessary fitting, have an attrition rate of approximately 40% (Cundiff et al., 2007; Cheung et al., 2016; Panman et al., 2016). As we are aiming to assess the effectiveness of self-management and not of the pessary itself, it will be important to minimise the early attrition associated with pessary treatment (e.g. discontinuation due to discomfort or failure to retain the pessary) and on which self-management would have no effect.

To maximise the likelihood of improving public health and increasing NHS efficiencies, the TOPSY study will pragmatically recruit women aged 18 or older, who use any pessary type/material (except Shelf, Gellhorn and cube pessary) and have retained the pessary for at least two weeks.

2. STUDY RESEARCH QUESTIONS AND OBJECTIVES

2.1 Research Questions

1. What is the clinical and cost-effectiveness of self-management of vaginal pessaries to treat pelvic organ prolapse, compared to standard pessary care on condition-specific quality of life? (RQ1)
2 What are the barriers and facilitators to intervention acceptability, intervention effectiveness, fidelity to delivery, and adherence for women treated with vaginal pessary and the health professionals who treat them, and how does this differ between randomised groups? (RQ2)

2.1 Objectives

1. To undertake a parallel group multicentre individual randomised controlled trial to test for superiority of pessary self-management compared to standard pessary care in terms of women's condition-specific quality of life.
2. To undertake an internal pilot study to ensure the trial can recruit, randomise and retain sufficient numbers of participants while delivering the intervention as planned.
3. To undertake a process evaluation in parallel to the trial to: maximise recruitment; assess eligible but non-randomised women; understand women's experience of and acceptability of the

interventions; assess adherence to allocated trial group; describe fidelity to intervention delivery; and identify contextual factors that may interact with intervention effectiveness.

4. To undertake an economic evaluation to establish whether pessary self-management is cost-effective compared to standard pessary care.

3. STUDY DESIGN

The research design is a multicentre, parallel group, superiority randomised controlled trial aiming to test the effectiveness and cost-effectiveness of self-management of vaginal pessaries for women with pelvic organ prolapse on condition-specific quality of life in comparison to standard pessary care. The study also contains 1) an internal pilot; 2) a nested process evaluation; and 3) a cost-effectiveness analysis.

3.1 Internal Pilot

The internal pilot will take place at six of the 17 trial centres already identified and the aim is to recruit 63 women in six months across those six centres. Each pilot centre should recruit one woman per month in the first three months and on average 2.5 women per centre in each of the following three months.

We will also seek permission (written consent) from approximately 12-18 women to audio-record the recruitment sessions (two to three women in each of the six pilot centres) to determine how the information about pessary use and the TOPSY study is delivered, and how consent is taken. This information will allow us to teach staff at all other centres the optimal way to recruit women to the study.

As part of the pilot study, and nested within the wider process evaluation: some self-management teaching sessions and follow up phone calls will be recorded (five to ten of each); some interviews with women who are randomised (approximately five); and, some interviews with women who are not randomised (approximately five) will be undertaken. The processes and rationale for this is detailed in the relevant process evaluation sections.

The primary **stop-go rules** to be applied following the internal pilot are:

- If the overall recruitment rate across pilot centres is 75% or more of the total expected recruitment (i.e. at least 47 out of 63) the trial will continue.
- If the recruitment rate is 50-75% (31-46 women), the trial will continue with a clear plan to overcome barriers to recruitment that is based on review of screening logs at centres, the trial protocol and the qualitative recruitment data (process evaluation).
- If the recruitment rate is 25-50% (16-30 women), screening logs, the protocol and the qualitative recruitment data (process evaluation) will be reviewed and the trial will only continue after discussion with and approval by NIHR HTA and with a clear plan to recruit within more centres and address the recruitment shortfall.
- Should recruitment be <25% (15 women or less), we will enter into discussions with NIHR HTA but it is not expected the trial will progress. The decision to stop the trial will be made by the TSC and the NIHR HTA.

In addition, we have set the following secondary targets:

- 40% of eligible new and 20% of eligible existing pessary users invited agree to randomisation;
- 60% of the pilot self-management women (n=19 of 31 women randomised to self-management) still self-managing at two week telephone follow-up (i.e. have removed and re-inserted their pessary at least once).

If the primary stop/go criterion is met but these secondary targets are not met, screening logs and qualitative data from the process evaluation will be reviewed, and recommendations will be made to the PMG, TSC and HTA for changes to the protocol. The trial will continue with these changes approved.

Seventeen centres have agreed to participate. Once 63 women have been randomised, the following will be reviewed:

- The centre numbers to ensure that 17 centres is sufficient to complete the randomisation of the remaining women in the available time, and if not we will adjust the number of centres as necessary.
- The standard deviation of the baseline PFIQ-7 to confirm that our estimated value of 50, which was used in the sample size calculation, is accurate.

The decision about whether to proceed with the trial will be made at month 12 in discussion with the HTA, the TSC and the DMEC.

3.2 Process Evaluation

The process evaluation will run throughout the internal pilot and the main trial. The process evaluation will use mixed methods (qualitative and quantitative), specifically: audio-recording of participant recruitment sessions (pilot study only), self-management teaching and self-management support phone calls; qualitative interviews with randomised women, healthcare professionals who recruit to the trial and deliver pessary self-management and standard pessary care, and women who decline to take part in the main trial; questions included within other means of data collection. The process evaluation purposes and methods of data collection are outlined in section 7.2. Qualitative analysis following Framework methods will be used for all qualitative data ("Process Evaluation Data Analysis" section 8.2); analysis of quantitative data arising from the process evaluation will follow principles laid out for the main trial section 8.1.

3.3 Cost-effectiveness analysis

Cost and resource-use data for all participants will be collected using a combination of routinely-collected NHS data and participant-completed questionnaires. The primary analysis will be undertaken at 18 months from an NHS perspective. It will include a cost-effectiveness analysis based on the primary clinical outcome measure (PFIQ-7) and using EQ-5D-5L to calculate quality adjusted life years (QALYs) in a cost-utility analysis. We will undertake longer term decision modelling analysis to examine costs and outcomes of pessary self-management compared to standard pessary care beyond the trial period.

4. STUDY POPULATION

4.1 Sample Size Calculation

A sample size of 330 women (165 per group) is required to provide 90% power to detect a difference of 20 points in the PFIQ−7 score at 18 months, assuming a standard deviation of 50, two-sided alpha of 0.05, and 20% loss to follow-up. In order to detect this standardised effect size of 0.4 SDs (20/50 points), 132 women will need to be recruited per group, or 165 per group to allow for dropout. Since the identified centres each see approximately 35 women a month for pessary care, we aim to recruit two to three women per month per centre.

The aim is to recruit a sample size sufficient to detect a 20-point difference in the PFIQ−7 score (the potential range of the PFIQ-7 is 0 to 300), although we acknowledge that an appropriate minimum clinically important difference for PFIQ−7 has not been formally determined. Barber et al. (2005) suggested a minimum clinically important difference (MCID) of 36 points, but it was emphasised that this was based on a small sample and applies only to within-group rather than between-group differences. Therefore, as part of the internal pilot, we will canvass PPI, investigators, and other participating clinicians to gauge the perceived importance of a 20-point difference.

Two recent trials which have used PFIQ−7 in populations of women using pessaries have reported SDs at 12 months and 24 months between 25 and 40 (Weigersma et al., 2014; Panman et al., 2016). These studies however were relatively small, conducted in only a few centres, and neither measured PFIQ−7 at 18 months. Given this uncertainty, a conservative assumption that the SD may be as high as 50 has been made. This will be monitored by examining the variability in the PFIQ−7 score for those women recruited in the internal pilot and we will continue to monitor the aggregated (blinded) SD regularly (every 40 cases using six-month outcome data; and as cases complete at 18 months), with a view to re-estimating the sample size at an appropriate point if required.

A sample size of 330 also provides power for the analysis of secondary outcomes, e.g. we will have more than 90% power (2-sided 5% level of significance) to detect a 20% difference in pessary use continuation rate (assuming at least 50% in the standard care group continue to use a pessary at 18 months).

4.2 Inclusion Criteria

- Women with pelvic organ prolapse of any type or stage
- Aged 18 years or older
- Have already been fitted with a vaginal pessary at their centre (all pessaries used in the NHS are CE marked)
- Pessary has been successfully retained for two weeks or more.

4.3 Exclusion Criteria

- Women who have been fitted with a shelf or Gellhorn pessary as these are difficult for women to remove and replace themselves
- Women with a cube pessary fitted as these require self-management
- Women lacking in manual dexterity, e.g. those with arthritis, or those with physical symptoms, such as leg spasm, which would affect their ability to remove and replace their own pessary
- Women judged by the treating healthcare professional to have a cognitive deficit such that it is not possible to obtain informed consent or self-management is not achievable
- Pregnant women
- Women who do not have sufficient understanding of the English language to understand the self-management instruction.

4.4 Summary of the sample

Women aged 18 years or older with pelvic organ prolapse, of any type or stage, who are being treated with a vaginal pessary that they have retained for at least two weeks will be invited to take part in the TOPSY study. We aim to recruit 330 women over 15 months in 17 centres (NHS outpatient, community, and primary care settings throughout the UK where women access pessary treatment for prolapse). We aim to recruit 63 women in six centres in the internal pilot study in the first six months of recruitment. Women will be randomised to either self-management or standard pessary care.

The sample of women for the process evaluation is described in data collection sections for each data set.

5 PARTICIPANT SELECTION AND ENROLMENT

5.1 Identifying and consenting women and Health Care Professionals

5.1.1 Context of Recruitment and Consent

Recruitment will take place in approximately 17 UK urogynaecology and gynaecology hospital-based, community-based or primary care-based centres where pessary services are currently delivered for women with prolapse. Patient Identifying Centres (PICs) may also be set up after the internal pilot stage to boost recruitment if required. Each centre will have a "Local TOPSY research team" led by a local Principal Investigator (PI). Members of the Local TOPSY research team will be NHS employees at the recruiting centre, and will be listed on the delegation log. Each local PI will ensure they have appropriately trained and qualified staff in the Local TOPSY research team to comply with the following centre responsibilities:

1. Identify women who could be eligible for the study (via patient notes, clinic lists or caseloads);
2. Provide potential participants with the relevant TOPSY study information;
3. Screen women for eligibility for TOPSY;
4. Contact potential participants (those that have returned an expression of interest form 02) and arrange a baseline clinic appointment (can coincide with women's usual pessary appointment);
5. Obtain written consent from willing participants;
6. Deliver the pessary self-management intervention to participants; and,
7. Undertake any study-related participant follow-up data collection and recording tasks.

TOPSY is a complex study containing a pilot study, main trial and process evaluation (which contains multiple components). To ensure participants are given sufficient information for the part(s) of the study they may be involved in; there are five Participant Information Leaflet (PIL)/ consent form pairs for the different components of the study (four for women participating in TOPSY and one for healthcare professionals who are interviewed in TOPSY.

Appendix B diagrammatically outlines the individual recruitment and consent pathways. The pathways are arranged chronologically. Each pathway is summarised below and described in detail in sections 5.1.3 onwards:

1. **PIL and Consent 01**: for the process evaluation, audio-recording of recruitment session between potential trial participants and the local recruiter (n=12-18 women in pilot study only)
2. **PIL and Consent 02**: for the main TOPSY trial (n=330 women), including an individual statement on the consent form that asks for consent relating to the process evaluation for audio-recording of self-management teaching sessions (n=30 women) and follow up phone calls (n=30 women) and willingness to be approached for an interview study.
3. **PIL and Consent 03**: for the process evaluation, interviewing women who are randomised and have initialled the statement on the main trial consent form indicating that they are willing to be approached for the interview study (n=36 women; 2 interviews each): self-management group (n=18 women) and standard pessary care group (n=18 women).
4. **PIL and Consent 04**: for the process evaluation, interviewing women who are potential participants but who decide not to take part in the main TOPSY trial (n=20 women).
5. **PIL and Consent 05**: for the process evaluation, interviewing healthcare professionals from TOPSY centres (aiming for a minimum n= 2 staff per centre, one staff member involved in recruiting women and one staff member who delivers the standard care and/or self-management intervention).

5.1.2 Participant identification

Potential participants for all patient focussed parts of the TOPSY study will be identified by a delegated member of the Local TOPSY research team by the following methods:

1. Reviewing patient notes, clinic lists or caseloads to identify women who are currently using a pessary and could be approached; and,
2. At a pessary appointment when women attend for pessary review (existing users) or are fitted with a pessary for the first time (new users).

TOPSY PPI representatives were keen that women would also be able to find out about TOPSY for themselves. Therefore, women may contact the TOPSY research team directly if they see posters in pessary clinics, or if they visit the TOPSY study website. In this instance, TOPSY staff will determine if there is a local study centre that the woman could attend and, if the woman agrees, her contact details will be shared with the specific centre. The local TOPSY research team at that centre will contact the woman and follow the recruitment pathway as required for that centre. If no local centre is available, women will be thanked for their interest in the study but will not be able to take part.

Potential healthcare professional participants will be identified from delegation logs at each centre.

5.1.3 Recruitment and consent to audio-record recruitment sessions (n=12-18, pilot study)

Only women who are approached to take part in the main TOPSY trial (please see 5.1.4 below) at their pessary appointment will be invited to take part in this component of the process evaluation. Based on work by the QuinteT group (Donovan et al, 2016), we aim to audio-record 12-18 "initial recruitment discussions" with women, as part of the pilot study, to determine how the information about potential participation in the TOPSY study is delivered and discussed. This will be on average 2-3 women in each of the six pilot centres. We expect this discussion to last between 10 to 15 minutes.

Potential participants will receive a one page participant information leaflet (PIL 01) about the audio-recording of the "initial recruitment discussion" from a delegated member of the local TOPSY research team. If willing to take part, written consent (Consent 01) will be gained prior to the session being audio-recorded. If women do not want to take part, the "initial recruitment discussion" will still take place, but will not be audio-recorded (See appendix B, Section 1)

5.1.4 Consent to the TOPSY trial (n=330)

Women who are identified as potential participants through the mechanisms listed in 5.1.2 above will be given a "recruitment pack" which contains an introductory letter, a participant information leaflet (PIL 02), an expression of interest form (EoI-02) and a reply paid envelope. Women identified via patient notes, clinic lists or caseloads will have the recruitment pack posted to them by the local TOPSY research team. Women identified at their pessary appointment will be given the same recruitment pack in clinic. If time restraints in clinic mean that the clinical care team are unable to fully discuss the study with the women after giving out a pack, they will ask if they can telephone them at a suitable time to discuss any questions they may have. If the women agrees, this will be taken as verbal consent for this phone call. This call will only be carried out by the local clinical care team. In addition, in both recruitment methods women can receive further information about participating at any time by using the contact details in the participant information leaflet (contact details for both the local centre and the TOPSY Glasgow study office will be provided). Once women have had enough time to make their decision, they can return the expression of interest form by post or in person to the local TOPSY clinical team to indicate if they are interested in participating or not. One reminder recruitment pack will be posted out if there is no response after four weeks. If women do not return the expression of interest after this reminder the local clinical team will call them to check they have received the information and to answer any questions. The current Cochrane review on recruitment interventions finds that

telephone reminders are effective in increasing recruitment to trials with low baseline rates of recruitment (Treweek S et al, 2018).

On receiving a positive expression of interest form, a member of the local TOPSY research team will discuss the study further with the woman, screen her for eligibility and arrange a baseline appointment if appropriate.

A short screening pro forma will be completed by a member of the local TOPSY research team to confirm if a woman is eligible to participate. For women who are new pessary users, eligibility screening will be finalised by telephone by a member of the local TOPSY research team, to assess if the pessary has been retained for at least two weeks. If the pessary has not been retained for two weeks standard centre protocol would be followed for further pessary care. If women indicate to the local research team that they remain interested in participating in TOPSY, eligibility will be reassessed once standard centre protocol is followed and the pessary has been retained for two weeks.

If a woman is eligible and has verbally reported that she is willing to take part, she will be asked to come to a baseline clinic appointment for consent, randomisation and completion of baseline questionnaires. Randomisation should occur within **4 weeks** of assessing eligibility. An existing pessary user attending a routine appointment can be screened, consented and randomised at the routine appointment if the women has had enough time to review the study information, discuss the information with the local research team and have all her questions answered and both the woman and the staff member recruiting agree.

Women who are eligible and attend the baseline clinic appointment will be asked to provide written informed consent for the main trial at that baseline appointment (Consent 02).

PIL 02 highlights to women that, if they are in the self-management group, a self-management teaching session or a follow up call may be recorded. There is a statement within Consent 02 ("**If I am in the pessary self-management group, I am willing for** the teaching session and/or telephone calls with the local TOPSY research team to be audio recorded") which women are asked to indicate 'yes/no' by initialling the relevant box. Women who mark 'no' to this statement can still take part in the study and their teaching appointments or follow up calls will not be recorded. Prior to recording an appointment or phone call verbal consent will be checked with both the woman and the healthcare professional. If both are agreeable a delegated member of the *Local TOPSY research team* will record

the teaching session/phone call using the digital recorder provided. The aim is to record 30 self–management teaching sessions and 30 phone calls (five to ten of each of these in the internal pilot study).

The main study consent form also asks women if they would be willing to being contacted about the interview study (see section 5.1.5). Women who do not consent to being approached for the interview study can still take part in the TOPSY study and they will not be approached for the interview study. (See appendix B, Section 2)

5.1.5 Recruitment and Consent for interview study with randomised women (n=36)

PIL two outlines that some women will be invited for interview. The main trial consent form (Consent 02) asks women who are willing to be contacted to hear more about an interview study (*I am willing to be contacted about taking part in an interview study*) to indicate 'yes' by initialling the box. Women will be purposively sampled for interview. The qualitative researcher (TOPSY study Stirling office) will post out the interview Participant Information Leaflet (PIL 03) and will call the woman a few days later to discuss their possible participation in the interview study. For those who agree to be interviewed after the discussion, a suitable time will be arranged for a face to face interview where the woman will be asked to provide written informed consent for the interview study (Consent 03). A repeat interview with each woman will take place at 18 months. Prior to that 18 month interview the woman will be phoned to ask for verbal consent for this second interview and to arrange a suitable time for the interview to take place. These procedures are as followed in the NIHR HTA funded OPAL (REC no; 13/WS/0048 and AMBER (REC No; 14/WS/011) trials to recruit women for interview when they had already agreed to take part in the trial and initialled their willingness to hear about the interview study on the consent form (See appendix B, Section 3)

5.1.6 Recruitment and consent for interview study with <u>non-randomised</u> women (n=20)

The main trial Participant Information Leaflet (PIL-02) contains a statement indicating that if women do not want to take part in the main trial, they may be approached to take part in a telephone interview study regarding pessary care and symptoms more generally. Only women who are invited to take part in clinic, and decline in clinic, will be asked to take part in this component of the study. They will be asked if they are willing to take a recruitment pack away for an interview study. Those who indicate that they are willing to take the pack away will be given a recruitment pack in clinic for

the interview study with non-randomised women. They will be asked to take the pack home to consider participation at their leisure.

The recruitment pack will contain an introductory letter, a Participant Information Leaflet (PIL four), an expression of interest form (EoI-04), a consent form (Consent 04) and two stamped addressed envelopes. Participants can opt into this component of the study by returning the expression of interest form (04) to the TOPSY study Stirling office in one of the stamped addressed envelopes provided. Those who do not return the expression of interest form will be deemed to be refusing participation and will **not be sent any reminders**. Those that return the form will be contacted by the TOPSY study Stirling office and the study explained further. If participants are willing to take part, the Stirling researcher will talk through the consent form with the woman on the phone and they will be asked to sign and return the consent form (Consent 04) to the TOPSY study Stirling office in the second stamped addressed envelope. On receipt of the consent form the Stirling researcher will sign the form and return a copy to the woman and a telephone interview will be arranged for a baseline interview. A second interview with each woman will take place 18 months after their first interview. Prior to that 18 month interview the woman will be phoned to ask for verbal consent for this second interview and to arrange a suitable time for the interview to take place. (See appendix B, Section 4)

5.1.7 Recruitment and consent for healthcare professional interviews

During site initiation visits, healthcare professionals who are identified as part of the Local TOPSY research team will be advised that they may be approached and invited to take part in an interview as part of the TOPSY study. Hence, healthcare professionals who are listed in the delegation log will be known to the TOPSY research team and will know that they may be approached for interview. All contact details for the Local TOPSY research team will be collected at the site initiation visit/prior to the centre being opened to recruitment. At least one healthcare professional who has delivered the self-management and/or standard care intervention at each centre and a sample of those who have been involved in other roles in relation to the study (e.g. recruitment) will be given an information leaflet (PIL -05) and invited to take part in a telephone interview study. A member of the TOPSY study Stirling office will phone the healthcare professional to answer their questions and to ask if they are willing to be interviewed. Those that are willing will be asked to complete and return the consent form. Once that written consent (Consent 05) is obtained a suitable date and time for telephone interview will be arranged. (See appendix B, Section 5)

5.2 Randomisation and web-based data management system

Randomisation will be minimised by age (<65/65+ years), pessary user type (new users/ existing users) and centre and will utilise the existing proven remote automated computer randomisation application at the study CTU - the Centre for Healthcare Randomised Trials (CHaRT, a fully registered UK CRN clinical trials unit) in the Health Services Research Unit, University of Aberdeen. This randomisation application will be available as an internet based service, located within the TOPSY data management system.

5.3 Consent and withdrawal of Study Participants

During all consent processes women (and healthcare professionals where applicable) will have the opportunity to ask any questions and be given further explanation about the study. Once written consent has been obtained for the main trial; one copy of the completed consent form will be placed in the woman's notes, one in the Trial Site File, one given to the woman, and one will be sent to the TOPSY Study Glasgow office. For women participating in the main trial, a letter will be sent to their GP, notifying them of their involvement. For all other parts of the TOPSY study once consent is obtained: one copy will be sent to/ retained by the participant, one will be put into the site file and one will be sent to the TOPSY study Stirling office.

Informed consent procedures will ensure that women and healthcare professionals understand participation is purely voluntary and that they can withdraw from all or any part of the research at any time without this affecting their participation in other parts, or their other medical treatment.

Women in particular may choose to withdraw from the treatment aspect of the study, but continue to provide data, for example by completing questionnaires. Where women cannot, or choose not to, continue to self-manage this will be recorded and women, where willing to do so, will continue to be asked to complete questionnaires.

If withdrawal occurs, the primary reason for withdrawal will be documented in the participant's CRF, if possible. After full withdrawal, no further data will be collected from the participant but data collected up to that point will be analysed.

If a participant is randomised and then withdraws prior to any intervention being undertaken, we will continue to include those women within their original allocated group, where data are available, in the intention to treat (ITT) analysis. If a woman is deemed ineligible post-randomisation, although her outcomes will still be assessed as per randomised, her treatment might revert to normal care if randomised to self-management. We will however monitor the extent to which both withdrawal and ineligibility post randomisation occurs and if it varies between the groups in the pilot study.

A change of status form will be completed in all of the above examples to indicate the nature of the withdrawal and to monitor participant attrition rates. Women in the self-management arm who withdraw from treatment will be referred back to the NHS.

5.4 Retention methods

Active measures to minimise loss to follow-up of participants include:

- Recording at the outset women's email addresses and mobile phone numbers, their preferred method of contact (for follow up contact) and their preferred method of completion of questionnaires. Questionnaires can be completed online (via an email link) or in paper format and returned by post.
- Participants who do not return their questionnaires within three weeks will be sent up to three reminders using a variety of methods (post/email/ text message dependent on participants preferred method). The third reminder will be by telephone where the researchers will aim to gather the primary outcome data during the call.

  Response rates to the self-reported questionnaires will be monitored to ensure they remain above 80%. If response rates are seen to drop, the team will discuss appropriate actions with the Project Management Group. Relevant action may include phone calls at different times of day or asking women to only complete the primary outcome measure.

To facilitate potential long-term follow-up of participants in the trial, women will be asked to consent (in the main trial consent form) to the research team accessing their NHS records via record linkage to ISD data in Scotland and NHS Digital in England. Any further follow up work would only be carried out after relevant regulatory review and approvals.

6 INTERVENTION

6.1 Pessary self-management

To support a woman to achieve the three tasks needed for self-management (medical management of the condition, role management and emotional management) the intervention will be directed at three levels:

- at **service level** to facilitate a supportive culture for a self-management treatment pathway.
- at **professional level** to ensure that staff have the self-management teaching and support skills.
- at **individual woman level** to ensure women can achieve the necessary tasks to self-manage.

Supporting delivery of self-management at service and professional levels

At service level, the TOPSY training team (a clinical co-applicant and the trial manager) will visit all trial centres and will inform staff in the centre about the trial processes and the self-management protocol. They will aim to talk to as many staff at the centre as they can to ensure that the staff at the centre as a whole know about the trial and about the philosophy of self-management. The training team will also develop a local trial implementation plan with those working directly on the trial to ensure that the trial processes work in the local centre context.

A training manual for those staff teaching women self-management has been developed with PPI input (including a focus group with women from the Royal College of Obstetricians and Gynaecologists (RCOG) PPI group, Women's Voices), through discussion with our clinical co-applicants (which includes urogynaecologists from across the UK, nurses and a physiotherapist), using International Consultation on Incontinence recommendations (Dumoulin et al 2017) and using best practice from the self-management literature. The manual outlines the key components of the self-management protocol. The training manual will sent to centres in advance so that intervention healthcare professionals have time to study it prior to centre visits. The TOPSY training team will go through the training manual with those who will deliver pessary self-management at each centre (the intervention healthcare professional) as part of the centre visits and the training manual will be part of the Investigator Site File (ISF). Fidelity to delivery of the self-management protocol will be assessed throughout the internal pilot and the main trial as part of the process evaluation.

Intervention components delivered to individual women

Each woman in the self-management group will receive a 30 minute, one-to-one **self-management teaching appointment** with an intervention healthcare professional (HCP) who has been trained in the pessary self-management intervention by the TOPSY training team. The teaching appointment should take place within **4 weeks** of the randomisation date. The intervention HCP is most likely to be a specialist nurse or physiotherapist, but may also be a urogynaecologist or GP. The self-management training manual specifies in detail the key components of the self-management intervention, facilitating standardisation of the self-management intervention across the centres. The key components as laid out in the training manual will be used by the intervention HCP when teaching women within the teaching appointment.

During the self-management teaching appointment, women will be given a **self-management information leaflet** containing written information on pessary self-management including diagrams of various pessary types and of pelvic floor anatomy. The leaflet will also contain information about common complications and what to do if these are experienced. The same written materials will be used across all centres. The leaflets initially developed as part of our previous non-randomised study (Kearney and Brown, 2014) were based on the viewpoints of, and feedback from, PPI representatives. These leaflets will be used in this study and have undergone further development drawing on the expertise of our TOPSY PPI representatives, Women's Voices (RCOG) focus group members and clinical co-applicants.

Women in the self-management group will be asked to **remove, clean and re-insert their pessary at least once in the two weeks following the self-management teaching appointment**. The intervention HCP who treated the woman will telephone her two weeks after the appointment and ask if she has been successful in removing, cleaning and re-inserting her pessary. They will discuss any difficulties experienced. If the woman has not changed the pessary, the healthcare professional will ask her to do so over the next week, and will call her again to check if this has been achieved. Where a woman has experienced difficulty that requires assessment by the healthcare professional or where the woman has not changed the pessary by the time of the second phone call she will be offered a second self-management teaching appointment. If, after this second appointment, the woman is unable to self-manage or does not wish to do so, she will be given the choice to transfer to standard pessary care. All information on these interactions with women and any subsequent cross-overs will be recorded in the CRF by centre staff. If it takes greater than 4 weeks (after the self-management teaching session)

to get in contact with the woman to complete the 2 week follow on call, the deviation log will be completed with reasons why. If the woman is not successfully contacted within 2 months of the self-management teaching session, the local clinician will contact the woman and inform her she has been reverted to standard care and a normal clinical appointment will be made for her.

Women in the self-management group will receive **a local telephone number** and **an email address** to use to make contact with the intervention healthcare professional at their centre if they experience any pessary problems or have questions (numbers of contacts received and details of reasons for calls will be recorded). Women's feedback from our previous non-randomised study suggested that one telephone call provided good support following the initial teaching appointment and access to a telephone number thereafter was adequate.

Once it is clear that the woman has been able to remove and re-insert the pessary at least once, she will be **asked to remove and re-insert the pessary at least once every six months**. This information will be given as part of the self-management teaching appointment and is written into the information leaflet. Women with PVC pessaries in both groups will receive a new PVC pessary every six months (women in the self-management group will receive their new pessary by post or by picking up a prescription). Silicone pessaries are more durable and are changed less frequently. Women with silicone pessaries in the self-management group will have the pessary changed by request if required (e.g. if the pessary becomes damaged) and women with silicone pessaries in the standard care group will have the pessaries changed as per local centre protocol. Self-management leaflets will include information about what women need to do if they require a new pessary out with these anticipated changes.

Women in both trial groups will be asked to complete questionnaires every six months which will include questions regarding their patterns of pessary removal and re-insertion. At **18 months** after randomisation women in both groups will attend a **clinic appointment** which will include an examination of vaginal tissues as in standard pessary care (see below).

6.2 Standard pessary care

Women will receive a clinic appointment for their pessary care according to the local management pathway. Content of appointments will follow local protocol which usually includes vaginal examination being performed to remove the pessary, inspection of the vaginal tissues and insertion

of a new pessary. Data on frequency of appointments and of pessary changes at appointments will be recorded in the CRF. Healthcare professionals who deliver standard pessary care at each centre will be interviewed as part of the process evaluation allowing variation in standard pessary care to be described.

7. DATA COLLECTION

Data collection in this section is described in three parts. Section 7.1 describes the data collected for the main trial, section 7.2 the data collection for the process evaluation component and section 7.3 the data collection for the health economic element of TOPSY.

Throughout the TOPSY study data will be gathered from both the women in the trial and the research staff at each study centre. Data will be collected on paper CRFs and sent to the TOPSY Study Glasgow office from centres. Women who opt to complete the questionnaires on paper will also post these back to the TOPSY Study Glasgow office. The data coordinator will check for data completeness and accuracy and enter to the data management system. For those participants who opt to complete questionnaires online, no further data entry is required. Centres will do minimal data entry, consisting mainly of entering screening information or information required for randomisation.

7.1 Data collection for main trial

Table 7.1 T*rial data collection summary*

**Data to be collected**	**Time-point**			
	**Baseline**	**6 months**	**12 months**	**18 months**
**Consent and randomisation**	X			
**Demographics and medical history**	X			
**Primary outcome**				
Condition-specific quality of life (PFIQ-7)	X	X	X	X
**Secondary outcomes (validated)**				
Generic quality of life (EQ-5D-5L)	X	X	X	X
Pelvic floor symptoms (PFDI-20)	X	x	x	X
Sexual function (PISQ-IR)	X	x	x	X
General Self-Efficacy (GSE)	X			X
**Secondary Outcomes (non-validated)**				
Pessary complications questionnaire	X	X	X	X
Pessary use questionnaire (to assess pessary use, acceptability and benefit)	X	X	X	X
Pessary confidence questionnaire (to measure pessary-specific self-efficacy)	X	X	X	X
Health Resource Questionnaire (uptake of additional prolapse treatment /support)		X	X	X
Telephone Support log (uptake of telephone support related to pessary use)	Continuous data collection			
Adherence to randomised protocol	Continuous monitoring			

Health of vaginal tissues (Vaginal examination in clinic)*	X			X
COVID-19 Survey**	Completed at first clinic visit once services have resumed			

* Women in standard care group will have vaginal tissues assessed at each clinic appointment as per standard practice. Women in the self-management group will have their vaginal tissues assessed at baseline and 18 month appointments.

** This will only be completed by women who had a standard care or 18 month appointment cancelled/postponed due to COVID-19. It may be posted/emailed out to women with their next due questionnaire booklet if centres have specific resource issues where this can't be competed at the first clinic visit (post COVID).

After the participants have consented, demographic and medical history data will be collected. Outcome measures on which data will be collected are described in the sections below.

7.1.1 Primary outcome

The primary outcome of condition-specific quality of life at 18 months post-randomisation will be measured in participant-completed questionnaires using the PFIQ-7. The PFIQ-7 (Barber et al., 2005) is a reliable, valid and responsive short-form of the PFIQ which measures condition-specific quality of life in women with pelvic floor disorders including urinary incontinence, prolapse and faecal incontinence. The participant-completed instrument includes items asking about the effect of bladder, bowel and vaginal symptoms on the woman's activities, relationships and feelings. There are three subscales (UIQ-7, CRAIQ-7, POPIQ-7), with each sub-score ranging from 0-100, and a total score ranging from 0-300. Data will be collected at each time-point to allow repeated measures analysis of the PFIQ-7 scores.

7.1.2 Validated secondary outcome measures

Several secondary outcomes will be collected as described below. Frequency of the collection of each outcome is shown in table 7.1.

Euroqol (EQ-5D-5L) (The EuroQol Group, 1990) will be used to measure participants' generic quality of life, complementing the primary outcome measure of condition-specific quality of life, and also providing data for the analysis of cost-effectiveness. The EQ-5D-5L is a two-part instrument. The first section, the EQ-5D descriptive systems contains five items: mobility, self-care, usual activities, pain/discomfort and anxiety/depression. The second part, the EQ-5D VAS, is a Visual Analogue Scale. Data will be collected at each time-point to give a complete profile of QALYs across the trial time-points, calculated using an area under the curve method (see section 8.3).

PFDI-20 will measure the severity of prolapse-related symptoms. This was developed and validated in parallel with the PFIQ-7 (Barber et al., 2005). It contains 20 questions about the presence of bladder, bowel and pelvic symptoms, and how bothersome these are. There are three subscales (UDI-6, CRADI-8, POPDI-6), with each subscore ranging from 0-100 and a total score of 0-300.

The Pelvic Organ prolapse/Urinary Incontinence Sexual Questionnaire (PISQ-IR) (Constantine et al., 2017), will be used to assess women's sexual symptoms. It contains 20 items with a score ranging from 0 to 48.

General Self Efficacy scale (GSE) (Schwarzer and Jerusalem, 1995) will be used to assess a woman's general self-efficacy (hypothesised to be a moderator of quality of life). This is a ten item scale with score ranging from 10 to 40.

7.1.3 Non-Validated secondary outcome measures

Non-validated questionnaires/data collection are described below:

Pessary Complications Questionnaire

A new pessary questionnaire (with 15 possible complications of pessary use), developed based on the literature, PPI opinion, and the team's experiences in the pilot study, will be used to assess women's pessary complications (e.g. discharge, odour, pain, discomfort, bleeding). Pessary complications are used to assess impact and safety of the trial interventions.

Pessary Use Questionnaire

A new questionnaire (includes nine questions**)** developed based on the literature, PPI consultation, and the team's experiences in the pilot study, will be used to assess the pattern of a woman's pessary use, including perceived acceptability and benefit. This will include questions that ask women: whether or not they are still using a pessary as treatment for prolapse; when they last removed and re-inserted their pessary; reasons for pessary removal; interference of the pessary with everyday life and if they find the pessary an acceptable treatment. Also included is a question adapted from the **Patient Generated Index of Improvement (PGI-I)** which will be used to assess perceived benefit of the pessary care regimens being evaluated. The PGI-I is a single-item tool asking the individual to rate the change in their condition since having treatment, which has been validated for urogenital prolapse (Yalcin and Bump, 2003; Srikrishna et al., 2010). An amended version asking women to describe how they feel about their pessary care since taking part in the study will be used, with response options ranging from very much better to very much worse.

Patterns of pessary use are used to measure impact, adherence, and acceptability of the trial interventions.

Pessary Confidence Questionnaire (to measure pessary specific self-efficacy)

No suitable condition-specific measure exists, thus we have chosen to develop questions relating to pessary self-efficacy based on the guidance from Bandura (1977). These six questions have been discussed with PPI representatives and will be reviewed further by PPI, statistical, PMG and clinical team members before use and in the pilot study. We will use both the generic validated measure of self-efficacy (GSE) and the responses to the developed pessary-specific self-efficacy questions to measure self-efficacy and help us understand the influence it has as a moderating factor on quality of life.

Uptake of additional treatment for prolapse

As an indicator of intervention effectiveness, the uptake of other treatment for prolapse since the start of the study, or treatment awaited, will be recorded in participant questionnaires (e.g. surgery, pelvic floor muscle training, oestrogen, lifestyle advice). Women's access to professional pessary-related support since starting the study will also be recorded (e.g. telephone support, a hospital appointment, a GP appointment). These data will be collected at all trial time-points to maximise reliability as they rely on women recalling events occurring over a period of some months. Additional treatment will be described as part of the main trial findings to assist in understanding adherence and the level of support women need as well as being used as part of the cost-effectiveness analysis.

Uptake of telephone support related to pessary use

Using a Telephone Support Log Form we will ask the intervention HCP who receives women's calls to record frequency and details of all calls received to the telephone support line. There will be a question in the pessary complication questionnaire that asks ALL women if they required telephone support as some women in the standard care group may also telephone for support from their local team. This will support understanding of adherence, effectiveness and level of support relating to the trial interventions.

Adherence to randomised protocol

Adherence to the self-management or standard care protocol will be monitored throughout the trial. Monitoring will be via multiple data sources: questions within the pessary use questionnaire;

telephone support contacts; health records). It will include cross-over to the other trial group (i.e. self-managing women opting to move to standard care). Standard care women will not have access to the trial self-management teaching and support intervention, but they may choose to remove and replace their pessary at home and this will be recorded in the pessary use questionnaire. More detailed exploration of adherence will be undertaken within the process evaluation section (section 8.2).

Health of vaginal tissues: At baseline and 18 months, women will have a vaginal examination undertaken at the clinic by a healthcare professional to assess the health of the vaginal tissues and identify problems associated with pessary use, for example, tissue granulation or ulceration.

7.1. 4 COVID-19 Survey

The COVID-19 pandemic resulted in the cancellation of regular urogynaecology clinic appointments for a minimum of three months. A short questionnaire has been developed to capture the impact this disruption to services has had on participants' views on pessary self-management/ clinic-based care. The questionnaire will be given to women for completion during their next clinic visit once services have resumed or may be posted/emailed out to women along with their next due follow up questionnaire (6, 12 or 18 months).

7.2 Data collection for Process Evaluation

The different components of the process evaluation data collection are described below:

Audio-recording of trial recruitment appointment (objective: maximise recruitment [Donovan et al, 2016]). Two to three recruitment appointments will be audio-recorded with consent from the healthcare professional and the woman in each of the six pilot centres (total n=12-18 sessions). If more than one person is undertaking recruitment at any of the pilot centres, recruitment will aim to sample for diversity across centres in professional background of recruiter. Sessions will be recorded using small, unobtrusive digital recorders. All six pilot centres will be asked to start recording recruitment sessions as soon as possible after the start of recruitment at their centre.

Audio-recording of self-management teaching appointments and self-management support telephone calls (objective: assess fidelity). Five to 10 teaching appointments and five to 10 follow up

phone calls will be recorded as part of the internal pilot and analysed with feedback given to all centres in order to maximise fidelity to delivery of the self-management protocol. A further 20-25 self-management teaching appointments and 20-25 phone calls (at least one self-management teaching appointment and one phone call will be recorded in each centre) will be audio-recorded in the main trial. Variance across the sample will be aimed for within pilot and main trial in treating healthcare professional (nurse/physiotherapist/doctor) and women's age. Small digital recorders will be placed, with consent of the woman and healthcare professional, in the consulting room or attached to the phone to record all instruction and support given. Consent for these recordings is included within the main trial consent and will be checked verbally before each recording.

Qualitative semi-structured face to face interviews with randomised women (objective: maximise recruitment, describe women's experiences/acceptability, adherence, contextual factors)

Thirty six women will be recruited (18 in the standard care group and 18 in the self-management group). Purposive sampling will aim for variance in age, treating healthcare professional (nurse/physiotherapist/doctor); and centre type (outpatient/community/primary care). The same number of interviewees for each trial group has been chosen to ensure that the groups as far as possible remain the same, since the interview could act as an intervention and unequal numbers in groups could cause a differential effect.

Interviews will be semi-structured and face to face and will explore: perspectives on recruitment (baseline); symptoms and quality of life (baseline)/ change in symptoms and quality of life (18 months); experience and acceptability of standard care or self-management (18 months); adherence to the allocated trial group (18 months); and contextual factors that are perceived to interact with effectiveness of the intervention (18 months). All interview schedules will be developed with our PPI co-applicant and other PPI representatives and Women's Voices. All interviews will be digitally recorded.

Qualitative semi-structured telephone interviews with women who decline randomisation (objective: maximise recruitment and assessment of non-randomised women). 20 women (including approximately five within the internal pilot) who are potential participants for the trial and do not consent to randomisation, but who do consent to taking part in an interview, will be interviewed at baseline by telephone using a semi-structured interview schedule. Sampling variance will be on woman's age and centre type (outpatient/community/primary care).

If women consent to future participation in the interview study they will also be interviewed by phone at 18 months. Interviews will focus on reasons for declining the trial entry (baseline); symptoms and quality of life (baseline)/ change in symptoms and quality of life (18 months); treatment received for prolapse (18 months); and contextual factors that may interact with future service implementation (baseline and 18 months).

Qualitative semi-structured interviews with healthcare professionals who recruit to the trial and deliver the interventions (objective: maximise recruitment, fidelity, contextual factors). During the internal pilot we will aim to interview at least one local recruiter in each pilot centre with findings fed back to all centres. In the main trial we will aim to interview, at least two staff involved in the trial at each centre, at least one of whom has delivered the self-management intervention. Sampling will aim for diversity of professional group both for recruitment and for delivery. Interviews, both during the internal pilot study and the main trial, will be semi-structured, last approximately 30 minutes and be undertaken by telephone. For recruiters, interviews will focus on factors that influence recruitment, including service structures. For those who have been involved in delivering standard pessary care and/or self-management, interviews will focus on experiences of delivering standard care/self-management, including variance in delivery and reasons for the variance; and contextual factors that were perceived to impact upon delivery

Questions within participant-completed questionnaires to randomised women (objective: assess experience/acceptability, adherence, contextual factors). As detailed in section 7.1 =, we will gather data within the pessary use questionnaire on acceptability and adherence to trial group. Within the pessary use questionnaire will be one open question that assesses women's experience of their trial group (standard care or self-management). The pessary confidence questionnaire (detailed in section 7.1.3) will form part of the process evaluation analysis to explore the moderating influence of self-efficacy on quality of life. .

7.3 Data collection for Economic Evaluation

Economic evaluation will be conducted alongside the main trial. For both groups, the EQ-5D-5L will be completed as part of the participant questionnaires at baseline, six, 12 and 18 months (http://www.euroqol.org/) to allow for the calculation of QALYs. Resource use will be captured by a combination of routinely-collected health data and participant-completed questionnaires (see table 7.2).

Overall costs will be estimated by multiplying resource use by unit costs obtained from the appropriate sources including trial specific costs, NHS reference costs, Unit costs of Health and Social Care and the British National Formulary (BNF).

Table 7.2 Resource use and outcome date collected for economic evaluation

**Pessary Self-Management group**		**Standard pessary care group**	
**Resource Use**	**Recorded by/how**	**Resource Use**	**Recorded by/how**
Training the healthcare professionals to deliver the self-management protocol in the trial	Study team – standard pro forma per training session to record how many staff at the session, location, time taken, any equipment required		
Appointment to fit initial pessary (staff grade and length of appointment to be recorded)	Captured from Patient Records by local research nurse	Appointment to fit initial pessary (staff grade and length of appointment to be recorded)	Captured from Patient Records by local researcher nurse
Initial 30-minute appointment with healthcare professional to teach women self-management (staff grades to be recorded)<br>Information leaflet given re. pessary management and symptoms/complications.	CRF completed by staff for each appointment. Include travel questions to women.		
Any unplanned/emergency contact (attendances or phone/email) with healthcare professionals for pessary care	Participant-completed questionnaire at 6, 12 and 18 months	Any unplanned/emergency contact (attendances or phone/email) with healthcare professionals for pessary care	Participant questionnaire at 6, 12 and 18 months
Phone call from healthcare professional 2 weeks after appointment. Other self-management support e.g. telephone, further teaching.	Recorded by staff on CRF		
Health Care Resource Use – Pessary and prolapse-related health care resource use (oestrogen, GP appointments, Nurse Appointments, Outpatient/Inpatient appointment, surgery,	Participant-completed questionnaire at 6, 12 and 18 months	Health Care Resource Use – Pessary and prolapse-related health care resource use (oestrogen, GP appointments, Nurse Appointments, Outpatient/Inpatient appointment, surgery,	Participant-completed questionnaire at 6, 12 and 18 months

telephone/ e-mail contact with healthcare)		telephone/ e-mail contact with healthcare))	
Follow-up appointment at 18 months (with who, for how long, record any treatment given or new pessary)	Patient records. Recorded on CRF.	Follow-up outpatient appointment at 6, 12, 18 months (with who, for how long, record any treatment given or new pessary)	Patient records. Recorded on CRF.
Out of pocket expenditure related to prolapse or pessary<br><br>Travel, time off work, parking, childcare to attend follow up appointments	Participant-completed questionnaire at 6, 12 and 18 months	Out of pocket expenditure related to prolapse or pessary<br><br>Travel, time off work, parking, childcare to attend follow up appointments	Participant-completed questionnaire at 6, 12 and 18 months

8 PROPOSED ANALYSES

8.1 Quantitative data analysis

All analyses will be conducted according to a pre-specified statistical analysis plan. All outcomes will be described with the appropriate descriptive statistics where relevant: mean and SD for continuous outcomes (or medians and interquartile range for skewed data), and counts and percentages for dichotomous and categorical outcomes.

The main effectiveness analysis will be based on the ITT principle. The analysis of the primary outcome will estimate the mean difference (with 95% confidence intervals) in the PFIQ−7 score at 18 months between the self-management and standard care groups using a mixed effects repeated measures model (which assumes incomplete outcome data to be missing at random). The model will incorporate PFIQ−7 scores at baseline, 6, 12 and 18 months, with age (<65/≥ 65) and pessary user type (new/existing) as fixed effects and recruitment centre as a random effect. Missing baseline data will be imputed. The repeated measures model will also estimate mean differences in PFIQ−7 at six and 12 months. Statistical significance will be at the 5% level.

The missing at random assumption for primary outcome data will be assessed further in sensitivity analyses. Treatment effects will be estimated under varying assumptions of data being missing not at random using pattern-mixture models. A complete case analysis will also be conducted.

Secondary outcomes will be analysed using an appropriate generalised linear model (for example binary logistic regression for dichotomous outcomes such as discontinuation with pessary (Y/N), and ordinal logistic regression for ordered categorical outcomes such as women's global impression of improvement (PGI-I). All models will be adjusted for minimisation covariates (age, pessary user type and centre) and baseline score (where applicable).

Given the potential for crossover, we will conduct a secondary analysis of compliers to estimate the effect of receiving the self-management intervention, using complier average causal effect (CACE) estimators. The CACE analysis will take a maximum likelihood approach, which can assume incomplete data to be missing at random, and can be adjusted for covariates. This analysis will provide unbiased effect estimates of receiving the self-management intervention, which will complement the unbiased ITT effect estimates of being offered self-management.

Subgroup analyses will be carried out within the following groups: Age (<65/≥65 years)hysterectomy (Y/N) and type of pessary user (new versus existing). Stricter levels of statistical significance (2P<0.01) will be sought, reflecting the exploratory nature of these analyses. Heterogeneity of treatment effects amongst subgroups will be tested for using the appropriate subgroup by treatment group interactions (Wang 2007).

A single main analysis will be performed at the end of the trial when 18-month follow-up has been completed. The independent DMEC will review confidential interim analyses of accumulating data at its discretion but at least annually.

8.2 Process Evaluation Data Analysis

Each data source will be analysed individually in the first instance to reach separate conclusions and findings then synthesised across data sources. All qualitative data sources will be transcribed verbatim and entered into Nvivo 11 for data management. Ten percent of transcripts within each data source will be coded independently by two analysts to assess for inter-rater reliability. All of the analysis

described below will be undertaken by the process evaluation subgroup of grant-holders. Analysis will not be shared with the wider grant-holding group until the main trial findings are revealed.

All **qualitative data** (audio-recordings of recruitment sessions; interviews with randomised women, women who declined randomisation and with healthcare professionals; and the open question within the questionnaire) will be analysed using the Framework Approach (Spencer et al., 2014) except for the audio-recordings of the self-management teaching appointments and the self-management follow up phone calls. Framework analysis will move through stages of data management, descriptive analysis and finally onto interpretive and explanatory analysis. For **each individual dataset** the recommended Framework stages will be followed:

<u>*Data Management*</u>

- *Familiarisation:* by listening to audio-recordings and reading transcribed data the analyst will familiarise themselves with the individual data set.
- *Construction of an initial thematic framework:* The thematic framework for each dataset will be developed from: the research questions; the data collection tools (e.g. interview schedules); themes that have arisen iteratively from the familiarisation process; discussion with co-applicants; and PPI consultation.
- *Indexing and sorting:* The thematic framework will be applied to all data within each dataset. The thematic framework will continue to iteratively evolve as indexing and sorting occurs.
- *Reviewing data extracts:* Involving PPI representatives and the qualitative analysis team the thematic framework will be reviewed and alternative ways of indexing the data considered.
- *Data summary and display:* Using framework tables (generated within Nvivo) each main theme will be displayed in a table(s). Some tables will have arisen from iterative analysis and others will arise from the purposes of the study. Specifically, there will be at least one table from each of the relevant data sources that focusses on: maximising recruitment, assessment of eligible but non-randomised women; experience/acceptability; adherence to allocated trial group; fidelity to intervention delivery; and contextual factors that influence effectiveness of standard pessary care/ self-management. For example, for maximising recruitment there will be a table each from the analysis of: recruitment sessions in the internal pilot study; interviews with randomised women; interviews with women who decline randomisation and interviews with recruiting healthcare professionals.

<u>*Abstraction and Interpretation*</u>

Abstraction and interpretation will occur first for each individual dataset and then the datasets will be combined.

- *Description (developing categories and mapping linkage):* Categories, within each data set, will be developed by reviewing tables and analytic memos, and by comparing and contrasting tables with a view to moving analysis onto a more interpretive level. Linkages will be made through exploration of how tables and the themes within them connect to one another. This process will be repeated for each of the purposes of the process evaluation to develop one overarching matrix for each purpose (O'Cathain et al., 2010) e.g. bring the tables together that focus on maximising recruitment into one matrix.
- *Explanation* will aim to bring the data together to interpret why the data have come together in the specific way that is presented. Analysis at this point will particularly focus on comparisons between standard care and self-management in ways that facilitate drawing overall conclusions about why self-management may or may not be effective, and which components of self-management are most important for future implementation.

Audio-recording of self-management teaching appointments and self-management follow up phone calls (fidelity) will be analysed by developing a structured analytic grid using the intervention protocols and the theory underlying the protocols. The grid will assess for key features within the teaching appointments and phone calls, for example does the healthcare professional teaching self-management offer the woman an opportunity to practice taking the pessary out and replacing it. The grid will contain explicit guidance as to what codes have to be applied in what circumstances. Coded data will then be subject to quantitative descriptive and interpretive analysis.

Analysis of secondary outcome self-report questionnaires to randomised women (experience/acceptability, adherence, contextual factors) will be analysed in line with the quantitative analysis described in section 8.1.

8.3 Economic Analysis

Cost-Effectiveness Analysis

The primary analysis will be undertaken at 18 months from an NHS perspective. All costs and outcomes beyond one year will be discounted at 3.5% (NICE, 2013). A broader perspective including women's personal expenditures will be included in a sensitivity analysis. Incremental cost-effectiveness ratios (ICERS) will be computed by comparing the costs and outcomes of the self-

management and standard care trial groups. The difference in effectiveness will be expressed in terms of the change in score on the primary outcome measure PFIQ-7 (cost-effectiveness analysis). The difference in utility between the two groups will be expressed in terms of QALYs calculated using the UK value set for patient-reported EQ-5D-5L data (Devlin et al., 2017).

This will be used in a Cost Utility Analysis to calculate the incremental cost per QALY gained.

Longer term Decision Modelling

To examine the costs and outcomes of self-management compared to standard care beyond the trial period we will undertake decision modelling. This will involve extrapolating data we have from the trial period and adding additional data from the literature and routine data sources. A five-year time frame would be used and the care pathway over this period will be mapped out. We will incorporate data on: number of women who would want to self-manage, continued pessary use, continuation rates for self-management, complications and adverse events, conversion to surgery rates for both self-management and standard care, health outcomes (prolapse and general quality of life outcomes), expenditure attending follow-up appointments in both groups, expenditure on replacement pessaries in both groups (type-dependent), other (potentially rare) outcomes of interest that we would unlikely to see during the 18 month trial period (e.g. fistula).

9 ORGANISATION - Trial coordination

9.1 Sponsor

Glasgow Caledonian University will now act as study sponsor and their finance department will be responsible for the financial management of the grant including agreeing contracts with collaborating institutions and submitting financial reports to HTA as required.

9.2 Clinical Trials Unit

The trial is supported by The Centre for Healthcare Randomised Trials (CHaRT Aberdeen). CHaRT has considerable experience with similar trials in this area and will develop the data management system, a remote randomisation system and will be responsible for ensuring the reliability of data at data-lock to ensure compliance with the Research Governance Framework and Good Clinical Practice.

9.3 Study offices

From June 2022, there will only be one TOPSY study office. This will be based at Glasgow Caledonian University.

9.4 Trial centres

Local Principal Investigator

Each collaborating centre will identify a lead clinician (local Principal Investigator) who will be the point of contact for that centre. The responsibilities of this person will be to:

- establish the trial locally (for example, by getting agreement from clinical colleagues; facilitate local regulatory approvals; identify, appoint, train and supervise staff who are recruiting at the site; and inform all relevant local staff about the trial)
- take responsibility for clinical aspects of the study locally (for example if any particular concerns occur relating to wellbeing of a participant)
- notify the TOPSY Study Glasgow Office of any unexpected clinical events which might be related to trial participation
- provide support, training and supervision for the 'local TOPSY research team'
- reporting any adverse events or serious adverse events
- represent the centre at any collaborators' meetings.

Intervention Healthcare Professional (HCP)

Each collaborating centre will appoint a local Intervention HCP as part of the local TOPSY research team who will be trained on the self-management intervention and who will keep regular contact with the local PI, with notification of any problem or unexpected development.

Each centre will have a centre initiation visit to ensure all study processes are in place before recruitment commences. The TOPSY Study Glasgow Office will set-up regular centre 'forums' for all centres to 'phone in' and discuss any problems experienced and share learning. Updates will be provided via quarterly newsletters. Centres having specific problems with recruitment and/or retention will be offered additional support either remotely or by an additional centre visit.

9.5 TSC and DMEC

An independent Trial Steering Committee (TSC) will review the study on behalf of the sponsor and the funder. The TSC will include an independent chair, clinicians with expertise in prolapse/pessary management, a member of the public and a member of the PMG; a representative from the sponsor and the funder will be invited to meetings of the TSC and minutes will be copied to the funder. The TSC will meet at least annually to supervise trial conduct to ensure the principles of Good Clinical Practice and relevant regulations are adhered to and will advise on the stop/go criteria specified in section 3.1.

A separate and independent Data Monitoring and Ethics Committee (DMEC) will be convened. This Committee will be independent of the trial organisers and the TSC. It is anticipated the members will meet once to agree terms of reference and then annually (or more frequently if required) to monitor accumulating data and oversee safety issues.

During the period of recruitment to the trial, the DMEC will review a report on accumulating safety data, together with any other analyses that the committee may request, at each meeting, and any serious adverse events reported to the DMEC as detailed in Section 10.1. In the light of this report, the DMEC will advise the Trial Steering Committee if, in its view, the trial should continue as planned, or stop early due to clear harm or benefit of a treatment, or external evidence; it may also make recommendations as to other amendments to the trial protocol based on this report.

The Chair and the other independent members of the TSC and DMEC are to be appointed after confirmation by the HTA.

9.6 Milestones

Table 9.1 shows the project milestones. Six-monthly reports will be submitted to the funder during the duration of the trial and the final report will be submitted at month 45. Other reports may be requested by the funder and will be submitted on request.

Table 9.1 Milestones

**Year one (Nov 2017-Oct 2018)**		
By month 3	Jan 18	Construct database, web-based data entry system, randomisation program<br>Establish first six centres (R&D negotiations, appoint local recruitment officers).<br>Collaborator agreements signed off<br>First TSC and DMEC (Joint)<br>Focus group with Women's Voices completed and analysed
By month 6	Apr 18	Staff training and teaching materials finalised. Study documents printed.<br>Centre visits at first six centres. Ready to start recruitment at first six centres
By Month 9	Jul 18	Recruitment open in six pilot centres with 18 women randomised<br>Record six to nine recruitment sessions<br>Record five self-management teaching appointments<br>Start recruitment of randomised women to interview study<br>Start recruitment of non-randomised women to interviews
By Month 12	Oct 18	Ten centres active and 75 women randomised (63 women from pilot centres and 12 women from additional centres)<br>Record 12-18 recruitment sessions<br>Undertake interviews with recruiters in each pilot centre<br>First annual report to funders<br>Complete analysis of recording data to support recruitment<br>Complete internal pilot study
**Year Two (Nov 2018 – Oct 2019)**		
By month 15	Jan 19	14 centres active 133 women randomised<br>Complete recruitment with women who decline randomisation<br>Second DMEC and second TSC<br>HTA provided with all information about internal pilot (including TSC and DMEC advice) and decision made regarding the best course of action
By month 18	Apr 19	17 centres active 229 women randomised
By Month 21	July 19	36 randomised women recruited for interview study
By month 24	Oct 19	Second annual report to funders<br>Interviews commence with healthcare professionals in recruiting centres<br>30 self-management teaching appointments recorded
**Year Three (Nov 2019 – Oct 2020)**		
By month 27	Jan 20	Recruitment complete in centres (n=330 women randomised)
By month 33	Jul 20	Questionnaire follow up at six months after randomisation completed<br>Complete data collection with women who decline randomisation

By month 36	Oct 20	Third annual report to funders<br>Third DMEC and third TSC
**Year Four (Jan 2021 – Jan 2022)**		
By month 39	Jan 21	Questionnaire follow up at 12 months completed<br><br>Interview follow up with women at 18 months complete (by March 21)<br>Interviews complete with healthcare professionals in recruiting centres
By month 45	Jul 21	Questionnaire follow up at 18 months complete
By Month 47	Sep 21	All data cleaning complete and database lock
By Month 50	Dec 21	End of study DMEC and TSC meeting<br>All centres closed down
By Month 51	Jan 22	Complete RCT analysis<br>Complete Process evaluation analysis (all data sources)<br>Complete Health Economic analysis<br>Data archiving and arrangements for long term follow-up<br>Dissemination – including final report to funder

9.7 Finance

The trial is supported by a grant from the NIHR Evaluation, Trials and Studies Coordinating Centre (NETSCC), Health Technology Assessment (HTA) Programme (HTA 16/82/01).

9.8 Confidentiality

All paper records will be kept in a secure storage area with limited access. The investigators and study centre staff involved with this study will not disclose or use for any purpose other than performance of the study, any data, record, or other unpublished, confidential information disclosed to those individuals for the purpose of the study.

Computers used to collate the data will have limited access measures via user names and passwords. Published results will not contain any personal data that could allow identification of individual participants.

9.9 Data Protection

All investigators and study centre staff involved with this study will comply with the requirements of the General data Protection Regulation(GDPR) and the Data Protection Act 2018 with regard to the collection, storage, processing and disclosure of personal information Access to collated participant data will be restricted to individuals treating the women, representatives of the sponsor(s) and representatives of regulatory authorities.

10. SAFETY REPORTING

Collaborators and participants may contact the Chair of the TSC through the TOPSY Study Glasgow Office about any concerns they may have about the trial. If concerns arise about procedures, participants or clinical or research staff (including risks to staff) these will be relayed to the Chair of the DMEC.

As the trial group to which participants are allocated cannot be masked from the participants or the centre staff after randomisation has occurred, unblinding is not an issue in this trial.
All women in the TOPSY study have had a vaginal pessary inserted. As a foreign body placed in the vagina, this is recognised as a potential cause of specific symptoms e.g. bleeding and vaginal ulceration/erosion.

Expected events arising from pessary treatment are noted below and thus will NOT be collected as adverse events but will be recorded:

- Granulation of vaginal tissue
- Involuntary expulsion of pessary
- Vaginal smell
- Vaginal discharge
- Bleeding during pessary change.

10.1 Procedure for reporting AEs and SAEs in TOPSY

DEFINITIONS

Adverse Event (AE)
Any untoward medical occurrence in a study participant, which does not necessarily have a causal relationship with the study intervention.

Adverse Reaction (AR)

Any untoward and unintended response that has occurred due to the intervention.

- Symptomatic and treated UTI (treated by HCP)
- Confirmed or suspected vaginal infection requiring time without pessary
- New or worsening urinary incontinence.
- New or worsening voiding dysfunction (except urinary retention requiring medical intervention)
- New or worsening defaecation dysfunction (except impaction requiring hospital intervention)
- Unscheduled vaginal bleeding
- Vaginal erosion or ulceration diagnosed by a healthcare professional and requiring time without the pessary
- Pain or irritation in the vagina or lower abdomen in the absence of infection
- New or worsening dyspareunia
- Vaginal fibrosis or constriction ring

For the purpose of the TOPSY study we will record any AEs that require the study participant to seek advice from a healthcare professional (e.g. common colds dealt with at home will not be reported) and which are NOT expected events of having a vaginal pessary as previously outlined.

A **serious adverse event (SAE), or Serious Adverse Reaction (SAR)** is any AE or AR which

- results in death;
- results in persistent or significant disability or incapacity;
- is life threatening (i.e. the subject was at risk of death at the time of the event; it does not refer to an event which hypothetically might have caused death if it were more severe);
- requires in-patient hospitalisation or prolongation of existing hospitalisation.

Serious adverse events potentially associated with a pessary include:

- Pessary entrapment in the vagina requiring removal in theatre
- Urinary retention requiring catheterisation
- Faecal impaction requiring hospital intervention
- Fistula: recto-vaginal or vesico-vaginal.

- Vaginal cancer
- Ureteric obstruction

Note: Hospitalisations for treatment planned prior to randomisation will not meet SAE criteria. Any hospitalisation post randomisation, will be recorded.

Pregnancy is not considered an AE or SAE, however if a woman becomes pregnant during the trial, they will be withdrawn from the study and pessary care advice would be given according to local standard care. This would be recorded on a TOPSY study change of status form.

10.1.1 Detecting AEs, ARs SAEs and SARs

All adverse events (including adverse reactions, serious adverse events and unexpected) must be recorded from the time a participant signs the consent form to take part in the study until the last follow up (18 months). (Expected events from having a pessary are recorded on study specific CRF's and not recorded as additional AEs).

In the standard care group, the local TOPSY research team will ask about the occurrence of AEs/SAEs at every pessary follow up appointment. Open-ended and non-leading verbal questioning of the participant will be used to enquire about AE/SAE occurrence. Participants will also be asked if they have been admitted to hospital, had any accidents, used any new medicines or changed medication regimens. If there is any doubt as to whether a clinical observation is an AE, the event should be recorded. Women in the self-management group are asked during the teaching appointment and advised in the information leaflet to call the telephone helpline if they experience any of the symptoms that may be indicative of an SAE/AE. The Pessary complication questionnaire completed by all women at all time-points will also capture any adverse events experienced.

10.1.2 Recording AEs and SAEs

When an AE/SAE occurs, it is the responsibility of the local PI to review all documentation (e.g. hospital notes, laboratory and diagnostic reports) related to the event. The local PI should then record all relevant information in the CRF and on the SAE form (if the AE meets the criteria of serious).

Information to be collected includes type of event, onset date, local PI assessment of severity and causality, date of resolution as well as treatment required, investigations needed and outcome.

10.1.3 Assessment of AEs and SAEs

Each AE must be assessed for seriousness, causality, severity and expectedness by the local PI or another suitably qualified physician in the local TOPSY research team who is trained in recording and reporting AEs and who has been delegated this role.

10.2 Reporting responsibilities of the local PI and CI

Once the local PI becomes aware that an SAE has occurred in a study participant, they will report the information to the TOPSY Study Glasgow Office within 24 hours. The SAE form will be completed as thoroughly as possible with all available details of the event and signed by the PI. If the local PI does not have all information regarding an SAE, they will not wait for this additional information before notifying the TOPSY Study Glasgow Office. The form will be updated when the additional information is received.

The SAE form should be transmitted by fax/email to the TOPSY Study Glasgow Office on 0141 331 8101/TOPSY@gcu.ac.uk.

If, in the opinion of the local PI and the CI, the event is confirmed as being related and unexpected, the CI will submit a report to the main REC, the trial sponsor and the DMEC within 15 days of the CI becoming aware of it.

Follow up procedures

After initially recording an AE or recording and reporting an SAE, the local PI will follow each participant's medical progress. Follow up information on an SAE should be reported to the TOPSY Study Glasgow Office when received.

AEs still present in participants at the last study appointment should be monitored until resolution of the event or until no longer medically indicated (as confirmed by the local PI).

11. END OF STUDY

The end of study is defined as the receipt of the last participant's 18 month follow up data. The end of the study will be reported to the REC, and R&D Offices and the sponsor within 90 days, or 15 days if the study is terminated prematurely. The investigators will inform participants of the premature study closure and ensure that the appropriate follow-up is arranged for all participants involved.

A summary report of the study will be provided to the REC within one year of the end of the study.

12. CONTINUATION OF INTERVENTION FOLLOWING THE END OF STUDY

When participants attend their appointment at 18 months, they will have a discussion with their healthcare professional about future care, including discussion about pessary self-management if this is desired and is an option locally. If a woman does not attend the 18-month appointment, the local recruitment officer will contact her to arrange a further appointment to ensure follow-up care is in place.

13. INDEMNITY

The Participant Information Leaflet provides a statement regarding indemnity for negligent and non-negligent harm.

Pessaries have been used in the NHS for many years and are a relatively safe intervention. Self-management is a system used widely in health care and no serious ethical issues are foreseen. Potential risks relate to possible serious pessary complications, specifically: fistula, incarceration of pessary, occurrence of vaginal cancer. Incarceration of pessary, fistulae and rare reported cases of cancer are associated with the pessary not being removed and replaced for a prolonged period of time. As participants (self-management group and standard care group) are asked back to clinic at 18 months this is the longest that a participant should have a pessary in situ and very long periods without care are avoided.

Participation in the study will help evaluate the training and teaching procedures and effectiveness of self-management. Taking part in this study does not affect normal legal rights. Whether or not women take part, the same legal rights apply as any other patient in the NHS (which includes professional indemnity insurance for negligence). If a participant wishes to complain about their health care or any aspects of this study, the normal NHS mechanisms will be available.

In addition, the universities involved with the trial hold and maintain a ‘no fault’ insurance policy. This policy covers all employees of the universities and those working under their direction.

14. PUBLICATION AND DISSEMINATION

The success of the trial depends entirely on the collaboration of a large number of women having pessary treatment for prolapse, as well as their healthcare team. For this reason, chief credit for the trial will be given, not to the committees or central organisers, but to all those who have collaborated in the trial. A trial publication policy will be developed. The results of the trial will be reported first to study collaborators.

The main funders report will be drafted by the Project Management Group and circulated to all collaborators for comment. The final version will be agreed by the Trial Steering Committee before submission for publication, on behalf of all the TOPSY collaborators. To safeguard the integrity of the main trial, reports of any explanatory or satellite studies will not be submitted for publication without prior agreement from the Project Management Group. Interest will be maintained in the trial by publication of newsletters at intervals for participants, staff and collaborators. Once the main report has been published, a lay summary of the findings will be sent to all involved in the trial.

To maximise the impact of the research, findings will be published in both practice based and high quality academic journals (such as The Lancet). Social media will be used to enable rapid dissemination of research results. The training manuals and any other training materials will be made freely available online and training days will be arranged. Where possible the PPI representatives will be involved in dissemination activities (training days, conference presentations).

15. REFERENCES

Abdel-fattah, M., Familusi, A., Fielding, S., Ford, J. and Bhattacharya, S. (2011) Primary and repeat surgical treatment for female pelvic organ prolapse and incontinence in parous women in the UK: a register linkage study. *BMJ Open.* 1(2), doi: 10.1136/bmjopen-2011-000206.

Bandura, A. (1977) Self-efficacy: Toward a unifying theory of behavioral change. *Psychological Review*, 84 (2), pp. 191-215.

Barber, M.D., Walters, M.D. and Bump, R.C. (2005) Short forms of two condition specific quality-of-life questionnaires for women with pelvic floor disorders (PFDI-20 & PFIQ-7). *American Journal of Obstetrics and Gynecology.* 193, pp.103-113.

Brown, C. and Kearney, R. (2015) Educational needs of healthcare professionals in caring for women with pelvic organ prolapse. A multi-professional survey. Poster Presentation IUGA Scientific Meeting. June 2015. Nice, France.

Bugge C, Adams L, Gopinath D, Kearney R Updating of Pessaries (mechanical devices) for pelvic organ prolapse in women – update is on-going and is based on: Bugge C, Adams L, Gopinath D, Reid F. Pessaries (mechanical devices) for pelvic organ prolapse in women. *Cochrane Database of Systematic Reviews* 2013(2):CD004010.

Bugge, C., Hagen, S. and Agur, W. (2017) Unpublished qualitative study about women's experiences of pessary for prolapse treatment. University of Stirling.

Bugge, C., Hagen, S. and Thakar, R. (2013) Vaginal pessaries for pelvic organ prolapse and urinary incontinence: a multiprofessional survey of practice. *International Urogynaecology Journal,* 24, pp. 1017-1024.

Cheung, R.Y.K., Lee, J.H.S., Chung, T.K.H., and Chan, S.S.C. (2016) Vaginal Pessary in Women With Symptomatic Pelvic Organ Prolapse: A Randomized Controlled Trial. *Obstetrics and Gynaecology*. 128(1), pp. 73-80.

Cundiff, G., Amundsen, C.L., Bent, A.E., Coates, K.W., Schaffer, J.I., Strohbehn, K. and Handa, V.L. (2007) The PESSRI study: symptom relief outcomes of a randomized crossover trial of the ring and Gellhorn pessaries. *American Journal of Obstetrics and Gynaecology.* 196, pp. 405 e1-e8.

Department of Health (2015) *NHS Reference Costs 2014-2015.* Available from https://www.gov.uk/government/publications/nhs-reference-costs-2014-to-2015 [Accessed 23.11.2017.

Devlin, N.J., Shah, K.K., Feng, Y., Mulhern, B. and Van Hout, B. (2017) Valuing health-related quality of life: An EQ-5D-5L value set for England. Office of Health Economics Research. Research Paper 16/01.

Donovan, J.L., Rooshenas, L., Jepson, M., Elliott, D., Wade, J., Avery, K, Mills, N. et al. (2016) Optimising recruitment and informed consent in randomised controlled trials: the development and implementation of the Quintet Recruitment Intervention (QRI). *Trials*. 17(1), pp. 283

Gorti, M., Hundelist, G. and Simons, A. (2009) Evaluation of vaginal pessary management: A UK-based survey. *Journal of Obstetrics and Gynaecology,* 29(2), pp. 129-131.

Hanson, L.M., Schultz, J., Flood, C.G., Cooley, B. and Tam, F. (2006) Vaginal pessaries in managing women with pelvic organ prolapse and urinary incontinence: patient characteristics and factors contributing to success. *International Urogynaecology Journal.* 17, pp. 155-159.

Hendrix, S., Clark, A., Nygaard, I., Aragaki, A., Barnabei, V. and McTiernan, A. (2002) Pelvic organ prolapse in the Women's Health Initiative: gravity and gravidity. *American Journal of Obstetrics & Gynecology.* 186 (6), pp.1160-1166.

Jelovsek, J.E. and Barber, M.D. (2006) Women seeking treatment for advanced pelvic organ prolapse have decreased body image and quality of life. *American Journal of Obstetrics and Gynaecology.* 194 (5), pp. 1455-1461.

Joint Formulary Committee: British National Formulary. London: BMJ Group and Pharmaceutical Press

Kapoor, D.S., Thakar, R., Sultan, A.H., Oliver, R. (2009) Conservative versus surgical management of prolapse: what dictates patient choice? *International Urogynaecology Journal.* 20, pp. 1157-61.

Kearney, R. and Brown, C. (2014) Self-management of vaginal pessaries for pelvic organ prolapse. *BMJ Quality Improvement Reports*. 3(1), doi:10.1136/bmjquality.u206180.w2533

Lone, F., Thakar, R., Sultan, A.H. and Karamalis, G. (2011) A 5-year prospective study of vaginal pessary use for pelvic organ prolapse. *International journal of gynaecology and obstetrics,* 114(1), pp. 56-59.

Lorig, K.R. and Holman, H.R. (2003) Self management education: History, definition, outcomes and mechanisms. *Annals of Behavioral Medicine*. 26(1), pp. 1-7.

Dumoulin, C., Bradley, C. et al. (2017) Adult Conservative Management. In: P.H. Abrams, L. Cardoza, A.E. Khoury, A. Wein eds. *6th International Consultation on Urinary Incontinence*. Plymbridge: Health Publication Ltd.

NHS Information Centre (2013) *Hospital Episode Statistics for England. Admitted Patient Care, 2012-13*. Available at: http://www.hscic.gov.uk/catalogue/PUB12566 [Accessed 23.11.2017]

NICE (2013) *Guide to the methods of technology appraisal*. Available from https://www.nice.org.uk/process/pmg9 [Accessed 21.03.2017].

O'Cathain, A., Murphy, E. and Nicholl, J. (2010) Three techniques for integrating data in mixed methods studies. *British Medical Journal*. 17, 341:c4587.

Olsen, A.L., Smith, V.,J. Bergstrom, J.O., Colling, J.C. and Clark, A.L. (1997) Epidemiology of surgically managed pelvic organ prolapse and urinary incontinence. *Obstetrics and gynecology.* 89(4), pp. 501-506.

Panman,C.M., Weigersma, M., Kollen, B.J., Berger, M.Y., Leeuwen, Y.L., Vermeulen, K.M. and Dekker, J.H. (2016) Effectiveness and cost-effectiveness of pessary treatment compared with pelvic floor muscle training in older women with pelvic organ prolapse: 2-year follow-up of a randomized controlled trial in primary care. *Menopause*. 23(12), pp. 1307-1318.

Ramsay, S., Tu, L.M. and Tannenbaum, C. (2016) Natural history of pessary use in women aged 65 – 74 versus 75 years and older with pelvic organ prolapse: a 12-year study. *International Urogynaecology Journal.* 27, 1201–1207.

Rogers RG, Rockwood TH, Constantine ML, Thakar R, Kammerer-Doak DN, Pauls RN, Parekh M, Ridgeway B, Jha S, Pitkin J, Reid F. A new measure of sexual function in women with pelvic floor disorders (PFD): the Pelvic Organ Prolapse/Incontinence Sexual Questionnaire, IUGA-Revised (PISQ-IR). International urogynecology journal. 2013 Jul 1;24(7):1091-103.

Ryan, P. and Sawin, K.J. (2009) The individual and family self-management theory: background and perspectives on context, process and outcomes. *Nursing Outlook.* 57(4), pp. 217-225.

Sacomori, C., Cardoso, F.L., Porto, I.P. and Negri, N.B. (2013) The development and psychometric evaluation of a self-efficacy scale for practicing pelvic floor exercises. *Brazilian Journal of Physical Therapy*. 17(4), pp. 336-342.

Sarma, S., Ying, T., Moore, K. (2009) Long-term vaginal ring pessary use: discontinuation rates and adverse events. *British Journal of Obstetrics and Gynaecology.* 116, pp. 1715–1721.

Schwarzer, R. and Jerusalem, M. (1995) Generalized Self-Efficacy scale. In J. Weinman, S. Wright, and M. Johnston. *Measures in health psychology: A user's portfolio. Causal and control beliefs*. Windsor, England: NFER-NELSON, pp. 35-37.

Spencer, L., Ritchie, J., O'Connor, W., Morrell, G. and Ormoston, R. (2014) Carrying out qualitative analysis. In J. Ritchie and J. Lewis, J. eds. *Qualitative research practice: A guide for Social Science Students and Researchers.* London: Sage Publications, pp.296-345.

Srikrishna, S., Robinson, D. and Cardozo, L. (2010) Validation of the Patient Global Impression of Improvement (PGI-I) for urogenital prolapse. *International Urogynaecology Journal***.** 21(5), pp. 523-528.

The EuroQol Group (1990) EuroQol-a new facility for the measurement of health-related quality of life. *Health Policy,* 16(3), pp. 199-208.

Treweek S, Pitkethly M, Cook J, Fraser C, Mitchell E, Sullivan F, Jackson C, Taskila TK, Gardner H (2018). Strategies to improve recruitment to randomised trials. *Cochrane Database of Systematic Reviews* , Issue 2. Art. No.: MR000013. DOI: 10.1002/14651858.MR000013.pub6.

Wang, R., Lagakos, S.W., Ware, J.H., Hunter, D. and Drazen, J.M. (2007) Statistics in medicine—reporting of subgroup analyses in clinical trials. The New England Journal of Medicine 357:2189–2194.
Weigersma, M., Panman, C.M., Kollen, B.J., Berger, M.Y., Lisman-Van Leeuwen, Y. and Dekker, J.H. (2014) Effect of pelvic floor muscle training compared with watchful waiting in older women with symptomatic mild pelvic organ prolapse: randomised controlled trial in primary care. *British Medical Journal*, 349: g7378.

Wu, J.M., Kawasaki, A., Hundley, A.F., Dieter, A.A., Myers, E.R. and Sung, V.W. (2011) Predicting the number of women who will undergo incontinence and prolapse surgery, 2010 to 2050. *American Journal of Obstetrics and Gynaecology.* 205(3), pp. 230.e1-5.

Yalcon, I. and Bump, R.C. (2003) Validation of two global impression questionnaires for incontinence. *American Journal of Obstetrics and Gynaecology* 189(1), pp. 98–101.

Zwerink, M., Brusse-Keizer, M., van der Valk, P.D., Zielhuis, G.A., Monninkhof, E.M., van der Palen, J., Frith, P.A. and Effing, T. (2014) Self-management for patients with chronic obstructive pulmonary disease. *Cochrane Database of Systematic Reviews* (3), CD002990, doi: 10.1002/14651858 [Accessed 23.11.2017].

APPENDIX A: TOPSY Contact Information and Oversight groups

**Chief Investigator**<br><br>Professor Carol Bugge<br>Department of Nursing<br>George Moore BuildingSchool of Health and Life Sciences<br>Glasgow Caledonian University Cowcaddens Road<br>Glasgow G4 0BA<br><br>**Tel:** 01413318796<br>**Email: Carol.Bugge@gcu.ac.uk**	**Co-Chief Investigator**<br><br>Dr Rohna Kearney<br>Consultant Urogynaecologist<br>Warrell Unit, St Mary's Hospital,<br>Oxford Road,<br>Manchester,<br>M13 9WL<br><br>**Tel:** 07793564172<br>**Email:** Rohna.kearney@mft.nhs.uk
**Co-Chief Investigator**<br><br>Professor Suzanne Hagen<br>Professor of Health Services Research<br>NMAHP Research Unit<br>Govan Mbeki building, Level 6<br>Glasgow Caledonian University<br>Cowcaddens Road<br>Glasgow G4 0BA<br><br>**Tel:** 0141 331 8104<br>**Email:** S.Hagen@gcu.ac.uk	**Trial Manager**<br><br>Dr Kirsteen Goodman<br>NMAHP Research Unit<br>Govan Mbeki building, Level 6<br>Glasgow Caledonian University<br>Cowcaddens Road<br>Glasgow G4 0BA<br><br>**Tel:** 0141 331 3516<br>**Fax:** 0141 331 8101<br>**Email:** Kirsteen.goodman@gcu.ac.uk
**Sponsor**<br><br>Professor Kay Currie<br>Associate Dean of Research<br>SHLS School Office<br>Govan Mbeki Building, Level 2<br>Glasgow Caledonian University<br>Glasgow G4 0BA<br><br>**Tel:** 0141 331 3472<br>**Email:** K.Currie@gcu.ac.uk	**Senior statistician**<br><br>Mr Andrew Elders<br>NMAHP Research Unit<br>Govan Mbeki building, Level 6<br>Glasgow Caledonian University<br>Cowcaddens Road<br>Glasgow G4 0BA<br><br>**Tel:** 0141 273 1307<br>**Email**: andrew.elders@gcu.ac.uk
**Funder**<br><br>National Institute for Health Research Evaluation, Trials and Studies Coordinating Centre<br>University of Southampton<br>Alpha House, Enterprise Road<br>Southampton<br>SO16 7NS<br>**Tel:** 023 8059 4259	**Clinical Trials Unit**<br><br>**Centre for Healthcare Randomised Trials (ChaRT)**<br>Health Services Research Unit<br>University of Aberdeen<br>3rd Floor, Health Sciences Building<br>Foresterhill, Aberdeen<br>AB25 2ZD

**Email:** netsmonitoring@nihr.ac.uk	**Tel:** 0 1224 438198<br>**Email:** chart@abdn.ac.uk
**Trial Statistician**<br><br>Dr Catherine Best<br>ISM and NMAHP RU<br>University of Stirling<br>FK9 4LA<br><br>**Tel:** 01786 466285<br>**Email**: catherine.best2@stir.ac.uk	**Health Economist**<br><br>Professor Helen Mason<br>Yunus Centre for Social Business and Health<br>George Moore Building, M201<br>Glasgow Caledonian University,<br>Glasgow<br>G4 0BA<br><br>**Tel:** 0141 331 8327<br>**Email:** Helen.Mason@gcu.ac.uk

TOPSY Study Trial Steering Committee

Name	Role in Committee	Job Title
Dr Lucia Dolan	Chair	Consultant Urogynaecologist
Mrs Valerie Taylor	Independent Member	PPI member
Dr Ann Capewell	Independent Member	Geriatrician
Dr Alex Wright-Hughes	Independent Member	Senior Medical Statistician
Dr Laura Ternent	Independent Member	Senior lecturer in Health Economics
Dr Carol Bugge	Non-independent Member	Associate Professor
Professor Suzanne Hagen	Non-independent Observer	Professor of Health Services Research
Dr Rohna Kearney	Non-independent Observer	Consultant Urogynaecologist
Dr Kirsteen Goodman	Non-independent Observer	Trial Manager

Dr Lois Thomas retired from the TSC at end of 2020.
Mrs Valerie Taylor stepped down from her role as PPI Rep from Nov 2021 and actively looking for a new PPI member (TBC June/July 2022).

TOPSY Data Monitoring Committee

Name	Role in Committee	Job Title
Dr Thomas Chadwick	Chair	Clinical Trials Statistician
Dr Barry O'Reilly	Independent Member	Consultant Obstetrician and Gynaecologist
Miss Pooja Balchandra	Independent Member	Consultant Urogynaecologist and Obstetrician

TOPSY Study – Co-Applicants

Professor Suzanne Hagen, Dr Rohna Kearney, Dr Wael Agur, Mr Andrew Elders, Miss Lucy Dwyer, Miss Ranee Thakar, Professor Doreen McClurg, Dr Helen Mason, Dr Karen Guerrero, Mr Aethele Khunda, Dr Christine Hemming, Ms Margaret Graham, Mr Mark Forrest, Dr Suzanne Breeman, Professor John Norrie.

APPENDIX B: The TOPSY study consent pathways.

The following five flow charts diagrammatically outlines the individual recruitment and consent pathways. The pathways are arranged chronologically. The Participant Information Leaflets (PILs) and consent forms used are summarised below and are referenced in the relevant flow chart.

1. **PIL and Consent 01**: for the process evaluation, audio-recording of recruitment session between potential trial participants and the local recruiter (n=12-18 women in pilot study only)
2. **PIL and Consent 02**: for the main TOPSY trial (n=330 women), including an individual item on the consent form that asks for consent relating to the process evaluation for audio-recording of self-management teaching sessions (n=30 women) and follow up phone calls (n=30 women) and willingness to be approached for an interview study.
3. **PIL and Consent 03**: for the process evaluation, interviewing women who are randomised and have initialled the statement on the main trial consent form indicating that they are willing to be approached for interview study (n=36 women; 2 interviews each): self-management group (n=18 women) and standard pessary care group (n=18 women).
4. **PIL and Consent 04**: for the process evaluation, interviewing women who are potential participants but who decide not to take part in the main TOPSY trial (n=20 women)
5. **PIL and Consent 05**: for the process evaluation, interviewing health care professionals from TOPSY centres (aiming for a minimum n= 2 staff per centre, one staff member involved in recruiting women and one staff member who delivers the standard care and/or self-management intervention).

Appendix B, Section 1:
TOPSY CONSENT pathway 1: Audio-recording of recruitment discussion
n=12-18 Audio-recordings

Part of the process evaluation in the pilot study only (2-3 per centre).

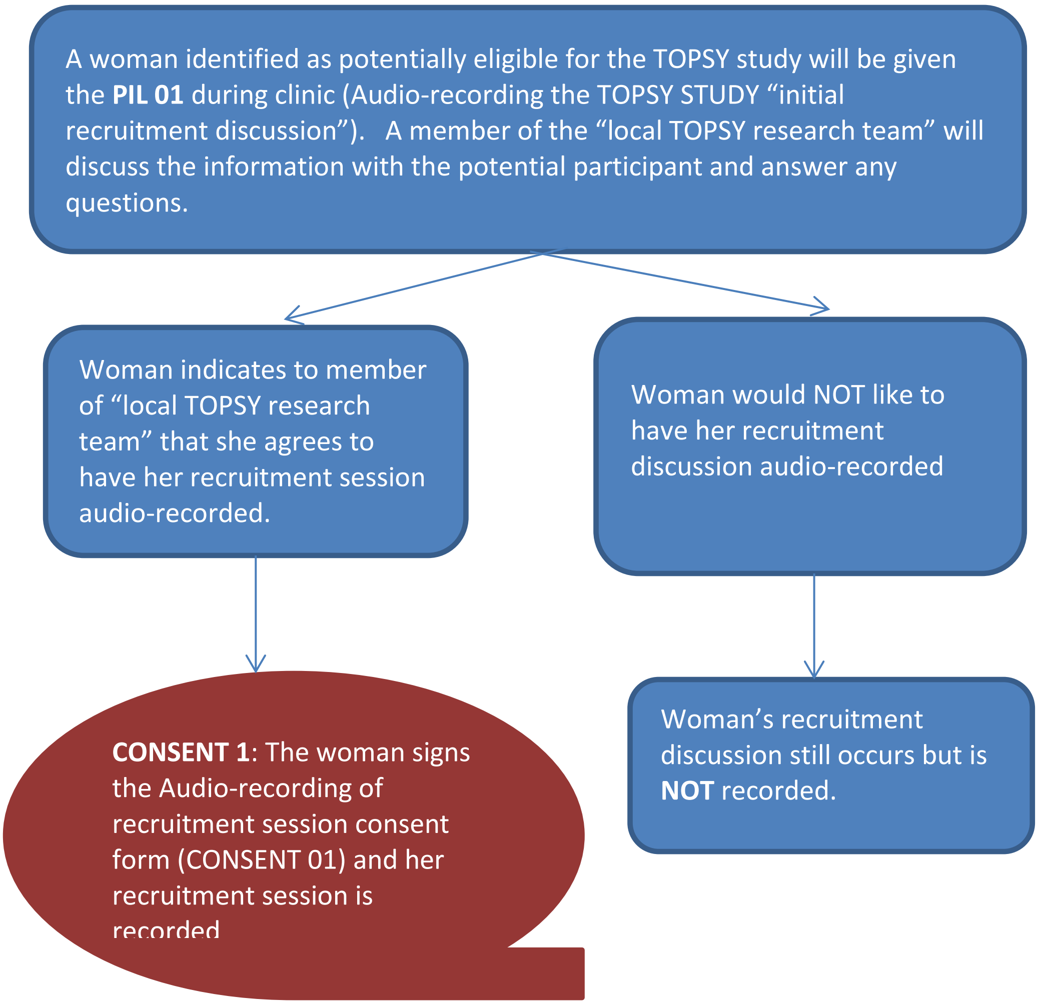

Appendix B, Section 2

TOPSY CONSENT pathway 2: Consent to the main study (n=330 women)

Note: Recruitment packs consists of; Main Trial PIL 02, invitation letter, expression of interest form 02 and reply-paid envelope.

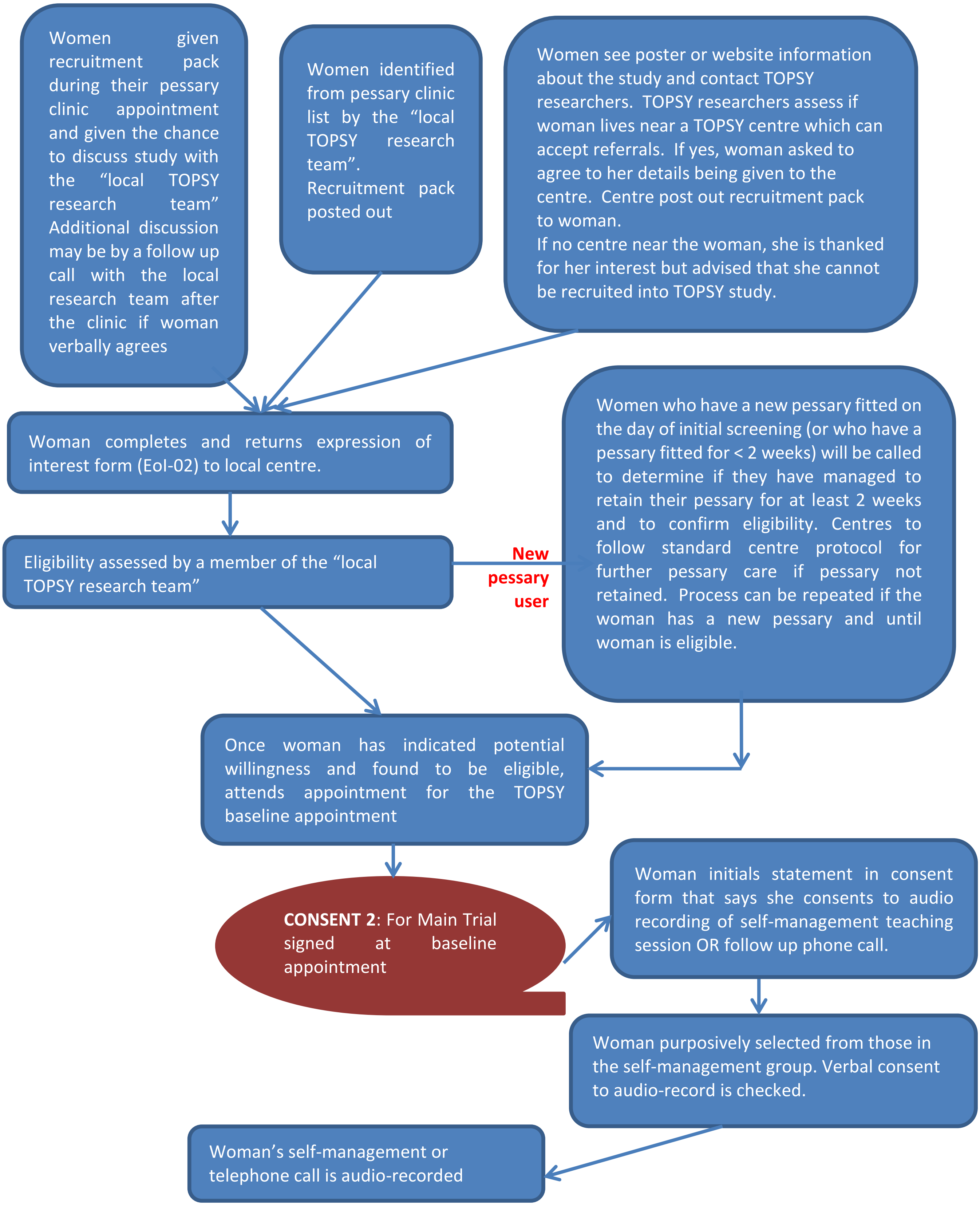

Appendix B, section 3:

TOPSY CONSENT pathway 3: Consent for woman to interview

(n=36 women): self-management group (n=18 women) and usual pessary care group (n=18 women)

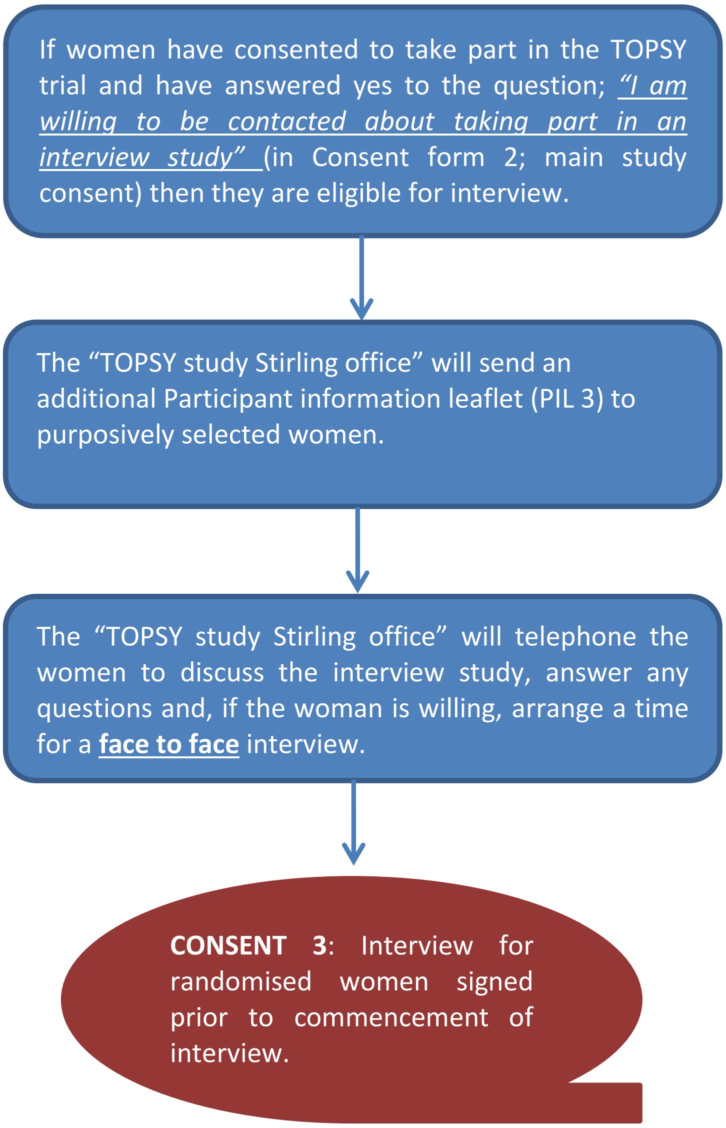

Note: Prior to 18 month interview participants will be contacted and consent to continue with 18-month interview will be checked verbally and a suitable time arranged to complete the second face-to-face interview.

Appendix B, section 4

TOPSY CONSENT pathway 4: Consent for interviewing non-randomised women (n=20)

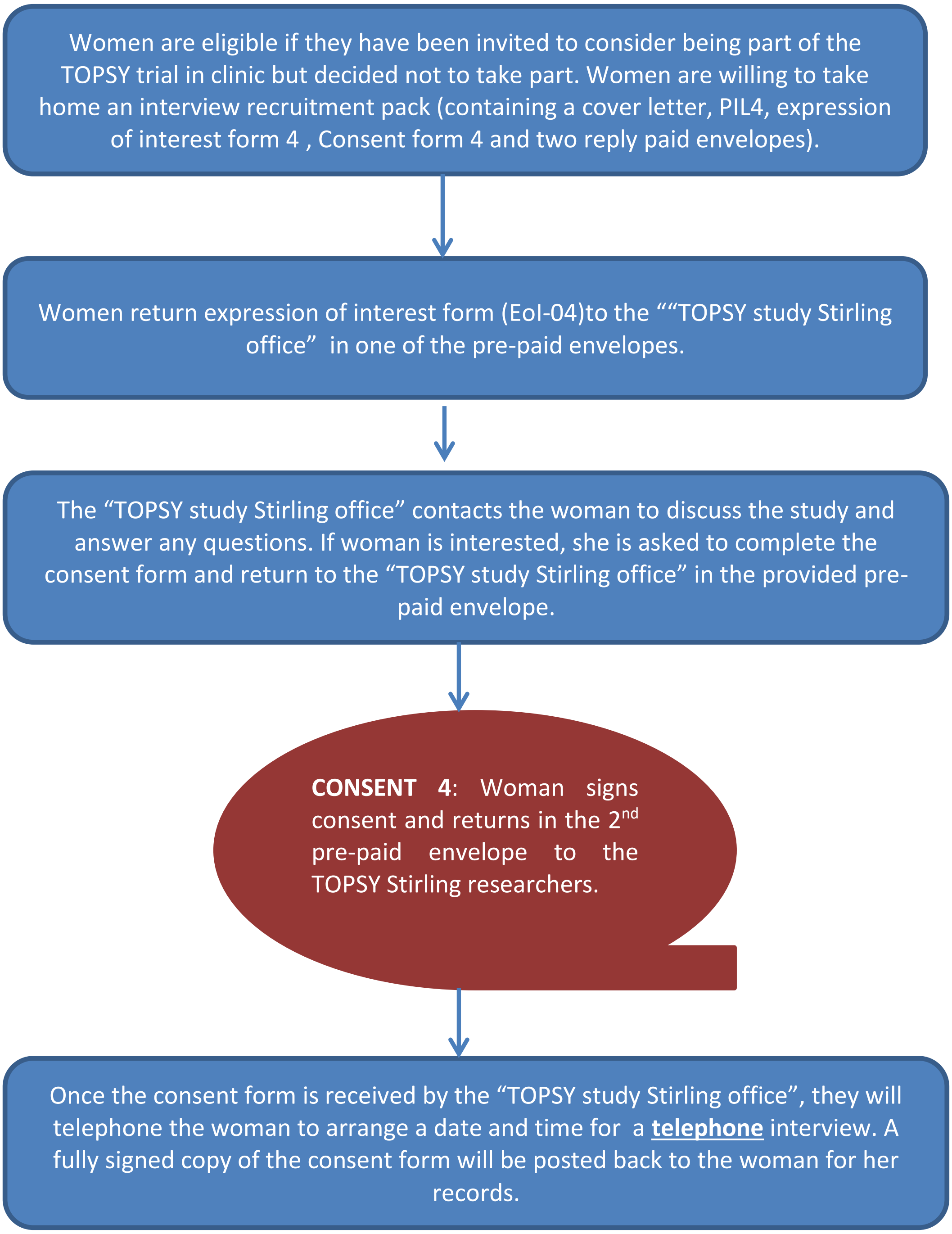

Appendix B, section 5

TOPSY CONSENT pathway 5: Consent for interviewing health care professionals from TOPSY centres

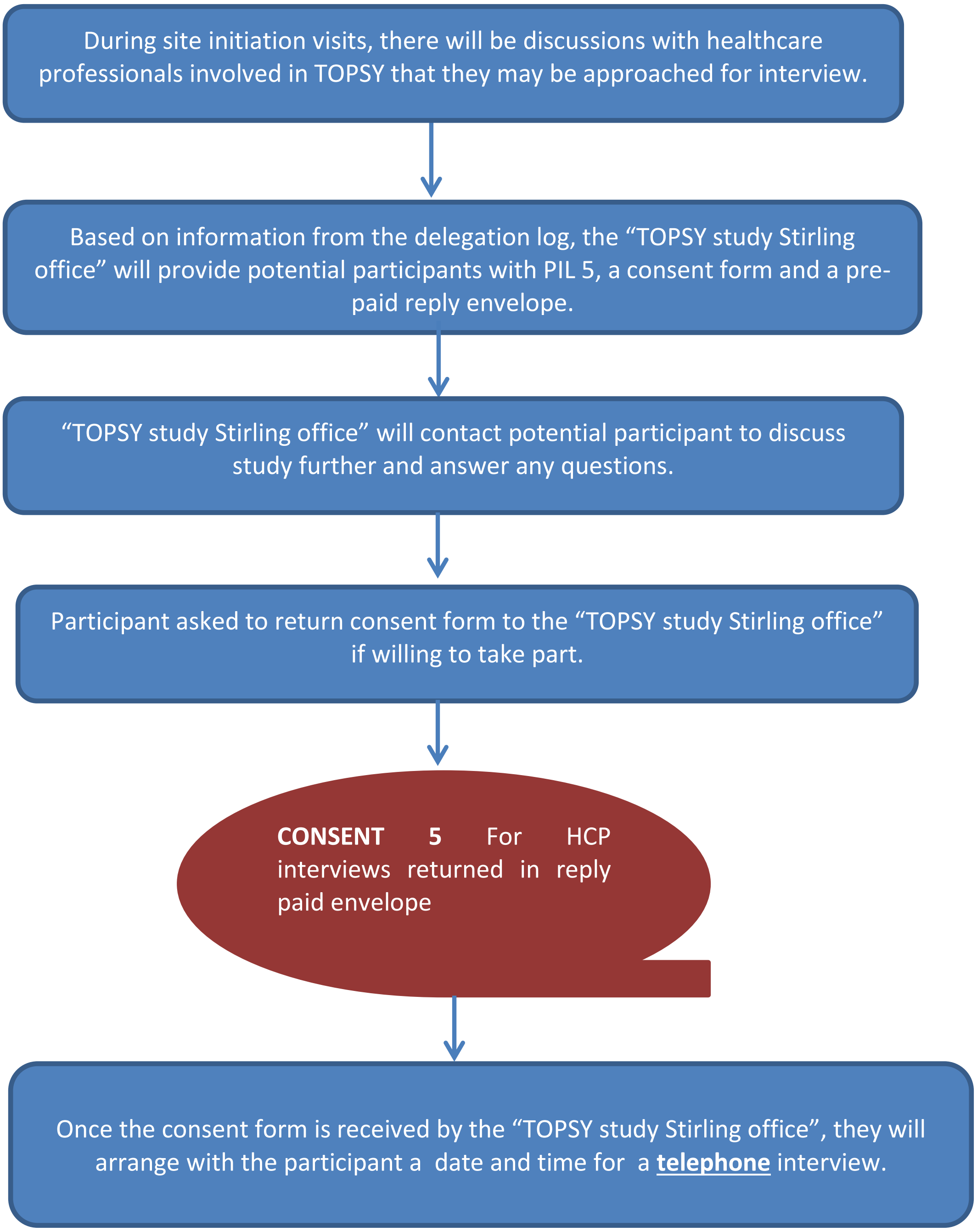

APPENDIX C- TOPSY COVID-19 Protocol Annex

At the time of the COVID-19 Pandemic, recruitment to TOPSY is complete. This document describes any differences in the processes undertaken during this time to make sure that there are no safety concerns and that the data integrity remains as high as possible.
The original text from the applicable sections in the protocol are noted first and then any changes to the process documented in red immediately after.

Section 5.4 retention methods

Active measures to minimise loss to follow-up of participants include:

- Recording at the outset women's email addresses and mobile phone numbers, their preferred method of contact (for follow up contact) and their preferred method of completion of questionnaires. Questionnaires can be completed online (via an email link) or in paper format and returned by post.
- Participants who do not return their questionnaires within three weeks will be sent up to three reminders using a variety of methods (post/email/ text message dependent on participants preferred method). The third reminder will be by telephone where the researchers will aim to gather the primary outcome data during the call.
  Response rates to the self-reported questionnaires will be monitored to ensure they remain above 80%. If response rates are seen to drop, the team will discuss appropriate actions with the Project Management Group. Relevant action may include phone calls at different times of day or asking women to only complete the primary outcome measure.

Section 5.4 retention methods (during COVID-19)

Questionnaires which were sent the week before "lockdown" will not have the first and 2nd reminders sent. Returned questionnaires are kept securely in the mail room at Glasgow Caledonian University and are delivered in batches to the data coordinator's house (these questionnaires do not contain patient identifiable information). For those who have not responded at the third reminder time-point, staff from the TOPSY office will call women to try and complete the questionnaires over the phone. For centres where letters of access are required for researchers to make these calls (very few), the centres will be asked to call the

women (if we know that resource is not an issue due to COVID-19) or the qualitative researcher (who has the relevant letters of access for each centre) will call the women. During the call, women will be offered the options of completing the questionnaire over the phone or if they provide an email address a link can be sent to them to complete online.

From the 11th May 2020, a member of the TOPSY team will access the study office and post out batches of questionnaires on a 6 weekly basis. For all batches of questionnaires sent from this date, reminder 1 (sent 3 weeks after the initial questionnaire) **will not** occur. We will post out a reminder at 6 weeks (previously the 2nd reminder) and then the 3rd reminder will be undertaken by phone as described previously.

A letter is being sent to all TOPSY participants with a COVID-19 update. In this letter it is stated that if a woman wants to complete her questionnaires online, rather than in paper format, she can email the TOPSY office with her email address.

If access to the TOPSY study office is stopped due to the continuing pandemic and we are unable to post out any further follow up questionnaires we will then explore the option of emailing links to all women we have email addresses for and calling those women we do not have email addresses for if they are due a follow up.

6.2 Standard pessary care

Women will receive a clinic appointment for their pessary care according to the local management pathway. Content of appointments will follow local protocol which usually includes vaginal examination being performed to remove the pessary, inspection of the vaginal tissues and insertion of a new pessary. Data on frequency of appointments and of pessary changes at appointments will be recorded in the CRF. Healthcare professionals who deliver standard pessary care at each centre will be interviewed as part of the process evaluation allowing variation in standard pessary care to be described.

6.2 Standard pessary care (during COVID-19 update)

All 21 centres have postponed pessary clinics for at least 3 months. All centres have let women know (either by letter or a phone call) what to do if they have any issues with their pessaries. Some centres are calling women when they would be due a follow up appointment to do a remote telephone follow up. If this happens, we are asking centres to complete the Telephone Support Log Form (CRF 07) which will capture the information that would be

collected if they attended clinic. If centres are not calling women as part of their standard care pathway we are not asking them to complete this CRF.

If women in the standard care arm call the centre and ask them to post a pessary out to them or to ask if they can attend their GP to get a pessary change, centres are asked to complete the Telephone Support Log Form (CRF 07) to capture this information. This information may be completed retrospectively.

7.1.3 Non-Validated secondary outcome measures

Uptake of telephone support related to pessary use

Using a Telephone Support Log Form we will ask the intervention HCP who receives women's calls to record frequency and details of all calls received to the telephone support line. There will be a question in the pessary complication questionnaire that asks ALL women if they required telephone support as some women in the standard care group may also telephone for support from their local team. This will support understanding of adherence, effectiveness and level of support relating to the trial interventions.

Uptake of telephone support related to pessary use (COVID-19)

As detailed in 6.2 the Telephone Support Log Form (CRF 07) will also be used if centres call women to do telephone follow ups during COVID-19.

Health of vaginal tissues: At baseline and 18 months, women will have a vaginal examination undertaken at the clinic by a healthcare professional to assess the health of the vaginal tissues and identify problems associated with pessary use, for example, tissue granulation or ulceration.

Health of vaginal tissues (COVID-19)

As all pessary clinics are postponed women can't attend their end of study (18 month) clinic visit to get their health of vaginal tissues checked. The following was proposed for all 18

month TOPSY end of study visits and has been approved by the sponsor and was rolled out to all centres on the 21st April 2020.

Since all centres have postponed pessary management clinics the TOPSY 18 month appointments will now be split into 2 parts;

1. Part 1- a telephone call where all end of study questions on the clinic visit from will be completed over the phone.
2. Part 2 – a clinic visit (when clinics are back up and running post COVID-19) where the vaginal examination will take place.

Centres who are due 18 month appointments in the next few months have all verbally agreed that they have the resource to call all women at 18 months. If during the pandemic resource at centres is affected and they are unable to call these women there are 2 options;

a) The lead urogyneacologist specialist nurse and co-applicant in Manchester could make the calls to these women to complete the 18 month visit over the phone. The nurse specialist would assess any safety concerns, report these to the relevant centre and thereby reduce any risks to participants of the centres not being able to contact these women.

b) If the lead urogyneacologist specialist nurse is unavailable or the centre would prefer, the process evaluation researcher (University of Stirling) has all the necessary letters of access in place for all centres. She could make the calls and report the clinical questions back to one of the clinical members of the research team at Manchester (most likely a clinical research fellow working with one of the Chief Investigators (Rohna Kearney)).

APPENDIX D - TOPSY 4 year follow up Protocol Annex

This annex describes the process of an additional 4-year follow up for TOPSY participants. This is an additional follow up time-point beyond the original funded 18 month end point.

Background and rationale for longer term follow up

All original objectives set out in the main protocol (Version 7, dated 23rd November 2020) for the 18 month TOPSY study will be met. This includes data on the effectiveness and cost-effectiveness of self-management when compared to standard care on the quality of life for women with pelvic organ prolapse (hereafter prolapse) at 18 months. However, following the current cohort of women in the TOPSY study for up to 4 years would provide evidence addressing four of the top 10 James Lind Alliance (JLA) uncertainties for pessary care (Lough et al, 2018) specifically:

- Priority number 1. How might a pessary affect sexual activity?
- Priority number 2. Do pessaries have an effect on the psychological well-being of women?
- Priority number 4. What are the risks and complications of pessary use for prolapse?
- Priority number 5. Are pessaries effective as a long-term treatment for prolapse?

The top 10 JLA priorities have been identified since TOPSY was commissioned. It is clear from those priorities that women, health care professionals and other relevant stakeholders have long-term follow up as a core issue for future pessary management.

***Research questio*n (RQ)**

This RQ is supportive of, and additional to, the original TOPSY trial. The long term follow up research question is:

- What is the longer term (4 years) clinical-effectiveness and cost-effectiveness of self-management of vaginal pessaries to treat pelvic organ prolapse, compared to standard pessary care on condition specific quality of life?

All women in TOPSY completed a series of outcome measures combined into one questionnaire at baseline, 6, 12 and 18 months. The questionnaire booklet for the 4-year follow up will contain the same series of outcome measures as the 18 months booklet with the addition of some questions about pessary use between 18 months and 4 years and the IPAQ-E questionnaire as a measure of physical activity.

The sections below are extracts from the main protocol and outline the updated sections within that original protocol (version 7, 23rd November 2020). These changes will apply to the 4-year follow up. They are documented here for clear identification of the additions.

4. STUDY POPULATION

4.1 Sample Size Calculation

As the primary outcome measure (PFIQ-7) remains unchanged then the sample size calculation for the original trial still applies to the long term follow up. We will conduct an intention to treat analysis including all 340 randomised participants, with imputed values for non-respondents, which will provide more than 90% power based on our original assumptions regardless of response rate at 4 years. If 264 women respond out of the 292 women who have consented to be approached at 4 years (90% response rate), then an observed cases analysis will also have 90% power (with two-sided alpha of 5%) to detect a difference in means of 20 points on the PFIQ-7 between the standard care and self-management groups, assuming a standard deviation for PFIQ-7 at 4 years of 50. We anticipate a high response rate given the current levels of retention, however a response rate of at least 65% would still provide more than 80% power in an observed cases analysis.

5 PARTICIPANT SELECTION AND ENROLMENT

All TOPSY participants who have not withdrawn from the study at the 18-month study time-point will be eligible and will be approached for follow up. All women who have participated in the TOPSY trial will be sent a summary of results newsletter from April 2022. Women were informed they would get this summary of results newsletter in the original participant information leaflet for TOPSY. Alongside this newsletter, a summary results cover letter, patient information leaflet and expression of interest form will also be sent which will include additional information about the 4-year follow up outlining what women are being asked to do i.e. complete one further questionnaire. Women will be invited to opt in should they wish to receive the 4 year questionnaire by either completing and returning the expression of interest form via mail, email or phone call as detailed in the flowchart below. If a woman returns her expression of interest form indicating she would like to complete the 4 year questionnaire, this will be sent on the required date (4 years from when first randomised to the TOPSY study).
Our retention process is the same as that detailed in Section 5.4 with one addition. SWAT 86 has been added for those who OPT in to the 4 year follow up. The SWAT 86 protocol is included as Appendix E. In summary, TOPSY participants remaining in the study 2 weeks prior to the distribution

of their 4 year questionnaire will be randomised to receive a pre-reminder or not to receive a pre-reminder. The mode of delivery of the reminder will be matched to the chosen mode of delivery of the follow-up questionnaire (email for those choosing to respond electronically and post, via a letter, for those choosing to self-complete a hard-copy questionnaire. A more detailed protocol for this SWAT can be found as Appendix E.

Appendix D, section 5
TOPSY Long term (4 year) Follow-Up Participant Selection and Enrolment

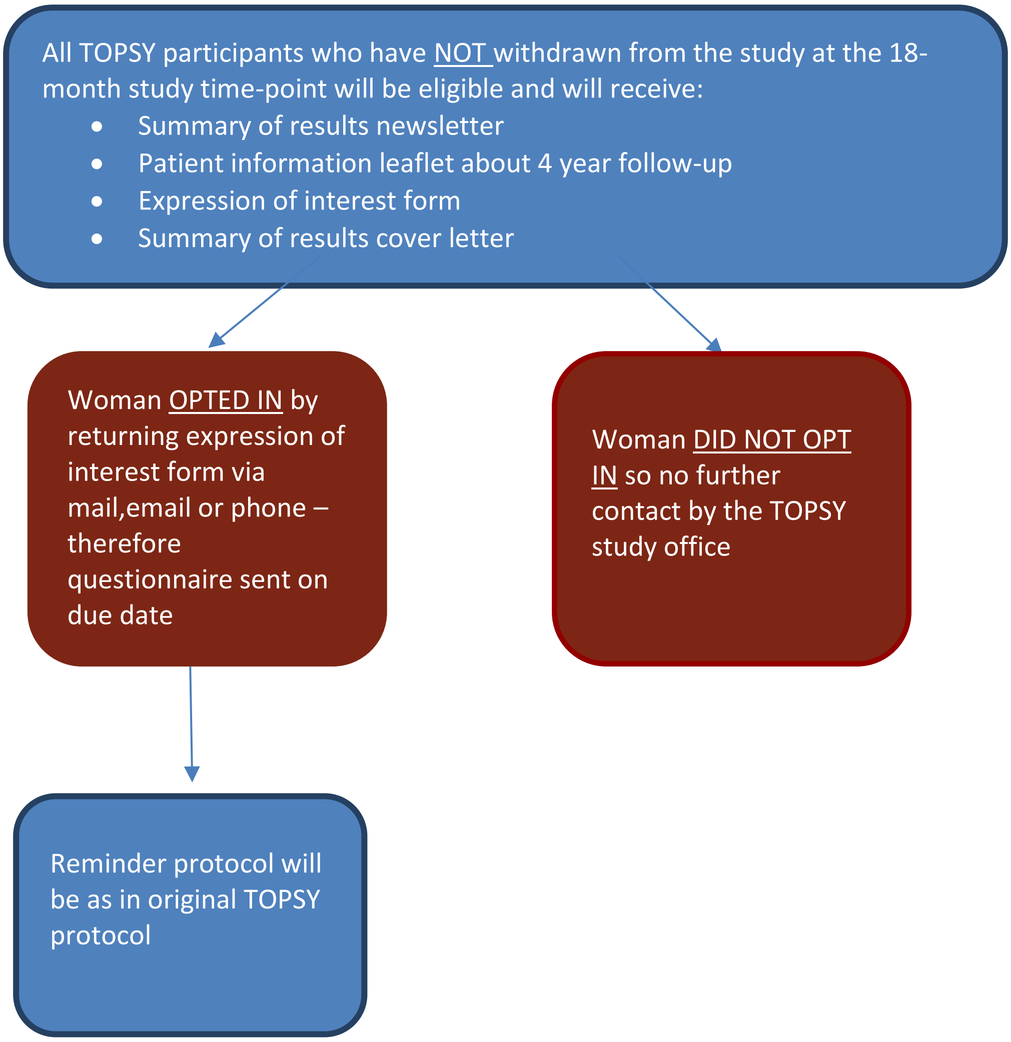

7. DATA COLLECTION

Section 7.1 in the protocol describes the data collected during TOPSY.

The first woman was randomised to TOPSY in May 2018, so the 4-year follow-up will commence in May 2022 and continue to October 2023.

Questionnaire Completion

Women selected how they would like to complete the questionnaire booklet (paper or via an online link via email) at randomisation and in letters to women sent to outline changes as a result of COVID-19. Women will be given the opportunity to change their preference for mode of completion should they wish to..

Outcome Measures

All measures utilised at the 18 month follow up would continue to be used at the 4-year follow up. Combined with the existing 18 month measures these data will be used to answer the long term follow up research question.

International Physical Activity Questionnaire modified for the elderly (IPAQ-E) (Hurtig-Wennlof et al, 2010) will be used to find out about the physical activities that people do as part of their everyday lives. The four questions ask about the amount of time in days and minutes, people spend sitting, walking, doing moderate and vigorous physical activity in the last 7 days. As the mean age of the sample is 64 years (SD 11 years) the IPAQ-E for the older age groups seemed more appropriate.

Section 7.3 outlines the data collection for the health economic element of TOPSY

For the 4 year follow up we will continue to ask about NHS resource use. These data will answer the cost-effectiveness elements of RQ1 along with the primary outcome measure and the EQ-5D 5L.

8 PROPOSED ANALYSES

8.1 Quantitative data analysis

All analyses will be conducted according to a pre-specified statistical analysis plan, in line with the original trial plan. All outcomes will be described with the appropriate descriptive statistics where relevant: mean and SD for continuous outcomes (or medians and interquartile range for skewed data), and counts and percentages for dichotomous and categorical outcomes. The IPAQ-E is an adaptation

of the International Physical Activity Questionnaire (IPAQ) and is suitable when participants include those aged 70 years or more. IPAQ-E data will be scored for all respondents using the same method as the original IPAQ with the number of MET minutes calculated in each case (Forde 2018). A MET (metabolic equivalent of task) is a standard unit of estimated resting energy expenditure. The mean number of MET minutes will be compared between randomised groups, using a linear mixed model adjusting for minimisation variables. Data will also be summarised according to the standard IPAQ categories of low, medium and high physical activity.

8.3 Economic Analysis
Cost-effectiveness analysis

The primary analysis will be undertaken at the end of the 4-year follow up from an NHS perspective. All costs and outcomes beyond 1 year will be discounted at 3.5% (NICE, 2013). A broader perspective including women's personal expenditures will be included in a sensitivity analysis. Incremental cost-effectiveness ratios (ICERs) will be computed by comparing the costs and outcomes of the self-management and standard care trial groups. The difference in utility between the two groups will be expressed in terms of QALYs calculated using the UK value set for patient-reported EQ-5D-5L data (Devlin et al, 2018). This will be used in a cost-utility analysis to calculate the incremental cost per QALY gained.

Longer-term decision modelling

The decision analytic model developed in the main TOPSY trial will be reviewed and updated with data from the 4-year trial measures where appropriate. We will investigate extending the model beyond the original 5 year follow up based on clinical advice on the potential care pathways for women and whether longer term self-management would be recommended.

9.4 Trial centres

Trial centres will remain open with a point of contact for any adverse event reporting or collecting data on any prolapse surgeries that occur during the 4-year follow up. No further information will be collected on women's standard clinic visits.

9.5 TSC and DMEC

The TSC and DMEC, including PPI participants, fully support the 4-year follow up. We will invite all members of the committees to stay involved (although there will be much less oversight required since there is only one additional questionnaire), however we propose at least 1 meeting a year for each of these committees.

9.6 Milestones

The milestones below show those of the 4-year follow up and time in months since the start of TOPSY in brackets.

Month 1 – 3 (Months 52-54)	Feb 22-May 22	All regulatory approvals/agreements, database development/user testing for 4 year outcomes and set up for recruitment of women into extension
Months 3-21 (Months 54-72)	May 22 – Oct 23	4 year follow up
Months 22-24 (Months 73-75)	Nov 23-Jan 24	4 year follow up analysis
Month 24 (Months 75)	Jan 24	Dissemination and final reporting.

10. SAFETY REPORTING

Women will not be asked about adverse events at clinics but TOPSY staff will review all responses in the 4 year questionnaires when they are completed and if any response indicates they may have had aserious adverse event that requires additional reporting we would contact the corresponding centre for more information. Any self reported adverse events noted by the women in the questionnaire which are not serious adverse events will be listed in a spreadsheet. This spreadsheet will be reviewed by the Clinical CI and this has been approved by the DMEC. This includes information on prolapse surgery repair for women who indicate in their response they have had this procedure. All other aspects of safety reporting remain as originally detailed in Section 10.

Additional References (Not already listed in main protocol)

The Independent Medicines and Medical Devices Safety Review (2020) ‘First Do No Harm’. https://www.immdsreview.org.uk/downloads/IMMDSReview_Web.pdf [Last accessed 29.06.21]

Forde C. (2018) Scoring the international physical activity questionnaire (IPAQ). The University of Dublin. https://tinyurl.com/IPAQscoring [Last accessed 11.02.22]

Hurtig-Wennlof A, Hagstromer M and Olsson, LA. (2010) The International Physical Activity Questionaire modified for the elderly: aspects of validitiy and feasibility. Public Health Nutrition. 13(11), pp1847-1854

Lough K, Hagen S, McClurg D, et al. (2018) Shared research priorities for pessary use in women with prolapse: results from a James Lind Alliance Priority Setting Partnership. BMJ Open 8: e021276. doi:10.1136/ bmjopen-2017-02127

APPENDIX E

SWAT 86: Advance notification of trial participants before outcome data collection to improve retention – ADAPTED FOR THE TOPSY STUDY

Objective of this SWAT

To evaluate the effects of a pre-notification letter or email on completion and return of outcome questionnaires.

Study area: Follow-up, Retention
Sample type: Participants

Estimated funding level needed: Medium

Background

Many trials struggle with participant retention and completion of follow-up questionnaires. A recent study found that the median (IQR) retention rate across 151 UK trials was 89% (79%-97%).[1] Reminders are generally an effective way of increasing response rates to questionnaires, with some evidence that pre-notification (contacting a participant in advance to say that they will be sent a questionnaire) also provides some benefit, although it is not high certainty evidence.[2] Therefore, there is no clear evidence that pre-notification is effective for trial retention,[3] nor whether any particular method (telephone, text, postcard, letter) of pre-notification confers any benefits over any other, although researchers have reported a lower odds of response following a postcard reminder than following a postal reminder to a survey, albeit after rather than preceding the original questionnaire mailing.[4]

There is also research on the content of contacts with participants, much of which relates to cover letters and post-reminders, although some relates to pre-reminders. Recent research on the content of cover letters by Duncan and colleagues developed a theory-based response letter intervention[5] using Michie's Theoretical Domains Framework (TDF)[6] and associated Behaviour Change Techniques (BCTs).[7,8] Evidence on the effectiveness of this approach is still inconclusive and has been included as a SWAT idea in the PROMETHEUS project (SWAT24).

It is therefore important to use what evidence is available to develop pre-notification interventions that might improve questionnaire return rates in trials and to evaluate these. Such pre-notifications should be developed using best current evidence in terms of mode of delivery and content, whilst practical considerations should, as ever, also be taken into account in the design and delivery.

This SWAT will test a pre-notification communication sent two weeks before participants are due to be sent their 4-year follow-up questionnaire in the TOPSY study, which is a pragmatic, multi- centre randomised trial testing self-management with clinic-based care for women who use a pessary for prolapse. The intervention to be tested is similar to that in SWAT 76, but will use a letter rather than a postcard in order to provide as consistent a form of pre-notification as possible between participants who opt to complete questionnaires postally and those who opt to complete electronically, whilst maintaining the patient choice of mode of communication.

The text in the pre-notification communication was informed by the theory and associated text used in the IQuad trial SWAT (SWAT 24). The reminder letter (or email) will be personalised to include the (typed) name of the participant because there is some evidence that personalising may improve response rates in surveys.[9]

Interventions and comparators

Intervention 1: Pre-notification communication in advance of follow-up questionnaire. Participants who elect to complete follow-up questionnaires online will be sent a personalised pre-notification in an email two weeks prior to the mailing of this. Participants who elect to complete follow-up questionnaires in hard copy form and return by post will be sent a personalised pre-notification letter. Similar wording and layout will be used in the email and letter.

Intervention 2: No pre-notification communication.

Index Type: Method of Follow-up

Method for allocating to intervention or comparator

Randomisation

Outcome measures

Primary: Valid response for TOPSY trial primary outcome (yes/no) (i.e. usable outcome data for the primary outcome measure.

Secondary: 1. Valid response for TOPSY trial primary outcome (yes/no) without reminder; 2. Number of reminders sent; 3. Time to response [or ceasing follow-up] (days); 4. Costs per participant retained.

Analysis plans

Baseline participant data, and the primary and secondary outcome measures will be summarised, using frequency (%), mean (SD) or median (IQR), as appropriate) both overall and by SWAT group allocation. For the analysis of the effect of the intervention, all randomised participants will be included in the analysis. Comparison of the primary outcome between the pre-notification letter group and the no pre-notification group will use binary logistic regression, including the randomised group factor and adjusting for stratification variables (TOPSY trial treatment allocation; chosen mode of response). Odds ratios and 95% confidence intervals for the between-groups difference in proportions completing the questionnaire will be estimated, and presented in conjunction with descriptive statistics of the number and percentage of respondents in each group. Analysis of the corresponding secondary outcome (valid response for TOPSY trial primary outcome without reminder) will be performed using the same method.

Time to response will be compared between the groups using Cox regression, adjusted for TOPSY treatment allocation and chosen mode of response. Data will be presented as a hazard ratio and related 95% confidence interval; median time to response in each group will also be presented. For the analysis of the difference in costs per participant retained (i.e. with a valid response for TOPSY trial primary outcome) between those randomised to pre-notification and those randomised to not be sent the pre-notification, costs will include the direct costs of printing the pre-notification letter, envelopes and postage, and the cost of staff time spent administering the mail out (for example filling and labelling envelopes for those who choose to receive questionnaires by post, sending emails to those who choose to receive questionnaires electronically). We will present a crude analysis of the ratio of the estimated between-groups difference in costs, divided by the corresponding difference in proportions providing valid responses for TOPSY trial primary outcome.

A meta-analytic framework will be undertaken by the TOPSY/PROMETHUS team to explore variability across different implementations of the pre-notification SWAT. Proportions of participants responding in each trial of a pre-notification card/letter/text intervention will be entered into a meta-analysis, and the heterogeneity of the intervention effect will be assessed using the I2 statistic. If substantial heterogeneity is demonstrated (I2 of 50% or greater), we will explore differences between trials that might explain that variation. The power of any such analyses may be limited if there are small number of trials, but in such an instance we will explore this issue qualitatively using data collected on the trial, the patient population, and the trial context.

Possible problems in implementing this SWAT

Retention to the host (TOPSY) trial for the initial stage of the study was extremely high. We do anticipate a slightly reduced retention rate (expected from longer term follow up studies), however, if retention rates stay high, there may be minimal effect seen by the proposed intervention.

References

1. Walters SJ, Bonacho dos Anjos Henriques-Cadby I, Bortolami O, et al. Recruitment and retention of participants in randomised controlled trials: a review of trials funded and published by the United Kingdom Health Technology Assessment Programme. BMJ Open 2017;7(3):e015276.
2. Edwards PJ, Roberts I, Clarke MJ, et al. Methods to increase response to postal and electronic questionnaires. Cochrane Database of Systematic Reviews 2009;(3):MR000008.
3. Brueton VC, Tierney JF, Stenning S, et al. Strategies to improve retention in randomised trials: a Cochrane systematic review and meta-analysis. BMJ Open 2014;4:e003821.
4. Sahlqvist S, Song Y, Bull F, et al. Effect of questionnaire length, personalisation and reminder type on response rates to a complex postal survey: randomised controlled trial. BMC Medical Research Methodology 2011;11:62.
5. Duncan A, Bonetti D, Clarkson J, Ramsay C. Improving trial questionnaire response rates using behaviour change theory. Trials 2015;16(Suppl 2):P92.
6. Michie S, Johnston M, Abraham C, et al. Making psychological theory useful for implementing evidence based practice. Safety & Quality in Health Care 2005;14:26-33.
7. Michie S, Johnston M, Francis J, et al. Mapping theoretically derived behavioural determinants to behaviour change techniques. Applied Psychology: An International Review, 2008;57(4):660-80.
8. Abraham C, Michie S. A taxonomy of behaviour change techniques used in interventions. Health Psychology 2008;27(3):379-87.
9. McColl E, Jacoby A, Thomas L, et al. Design and use of questionnaires: a review of best practice applicable to surveys of health service staff and patients. Health Technology Assessment 2001;5:31.
10. Lerner D, Amick B, Rogers WH, et al. The Work Limitations Questionnaire. Medical Care 2001;39(1):72-85.

implementation of this SWAT

Original SWAT contact details:People to show as the source of this idea: Chris Sutton, Sarah Cotterill, Denise Forshaw, Sarah Rhodes, Alison Hammond

Contact email address: Chris.J.Sutton@manchester.ac.uk
Date of idea: 31/AUG/2018

SWAT adapted on 27-07-22 for inclusion in the TOPSY trial (all references of WORKWELL study removed; original SWAT 86 protocol can be found here; https://www.qub.ac.uk/sites/TheNorthernIrelandNetworkforTrialsMethodologyResearch/SWATSWARInformation/Repositories/SWATStore/).

APPENDIX F – The APPLE study, a sub-study of TOPSY (Funded Variation to Contract Three)

This annex relates to a sub-study of the main TOPSY trial. In response to an 'opportunity for additional research call – Dementia and Neurodegenerative Disease' call from NIHR the TOPSY team developed a feasibility study to explore the possibility of developing an intervention for women who use a pessary for treatment of prolapse and who also have dementia. The acronym for this satellite study is the APPLE study (dementiA and Pessary for ProLapsE).

Full Study title

Building an intervention to improve the quality of life of women who have dementia and who use a pessary for treatment for pelvic organ prolapse (the APPLE study)

Funding: NIHR HTA (16/82/01) (VTC 03)

Chief Investigator: Professor Carol Bugge

APPLE Sub-study summary

Study Title	Building an intervention to improve the quality of life of women who have dementia and who use a pessary for treatment for pelvic organ prolapse (the APPLE study)
Study Design	Mixed methods feasibility study
Study Participants	**In this Annex:** Health and social care professionals who care for women who use a pessary for prolapse and have dementia<br>**Addressed Elsewhere:** Women with dementia and their informal carers will also be part of the study and their inclusion is being considered as part of an application to the Scotland A ethics committee
Planned Size of Sample	Health and Social Care Professionals: 10-15
Planned Study Period	1 September 2022 – 31 August 2023
Research Question/Aim(s)	**In this Annex**<br>• To understand which services currently deliver pessary care for women with dementia and how those services manage/support the women and their unpaid carers.<br>**Addressed Elsewhere**<br>• To understand what women with prolapse and dementia and/or their unpaid carers would like from services to treat the woman's prolapse symptoms.<br>• To investigate the feasibility of using routinely collected data to understand the scale of the problem.

	• To develop a team (grant holders, stakeholders and Patient and Public Involvement and Engagement [PPIE] members), who have the necessary expertise, for a future large-scale, robust grant application that will aim to improve the quality of life for women who have dementia and use a pessary for prolapse treatment.

Glossary of Abbreviations

NICE- The National Institute for Health and Care Excellence

UK- United Kingdom

HCP – healthcare professional

LPA – Lasting Power of Attorney

TOPSY- Treatment Of Prolapse with Self-care pessarY

NHS – National Health Service

PI – Principal Investigator

ENRICH- ENabling Research In Care Homes

HSCP – Health and Social Care Professional

SCP- social care professional

RF- Research Fellow

PPIE - Patient and Public Involvement and Engagement

GCU- Glasgow Caledonian University

REC- Research Ethics Committee

R&D – Research and Development

Flow diagram

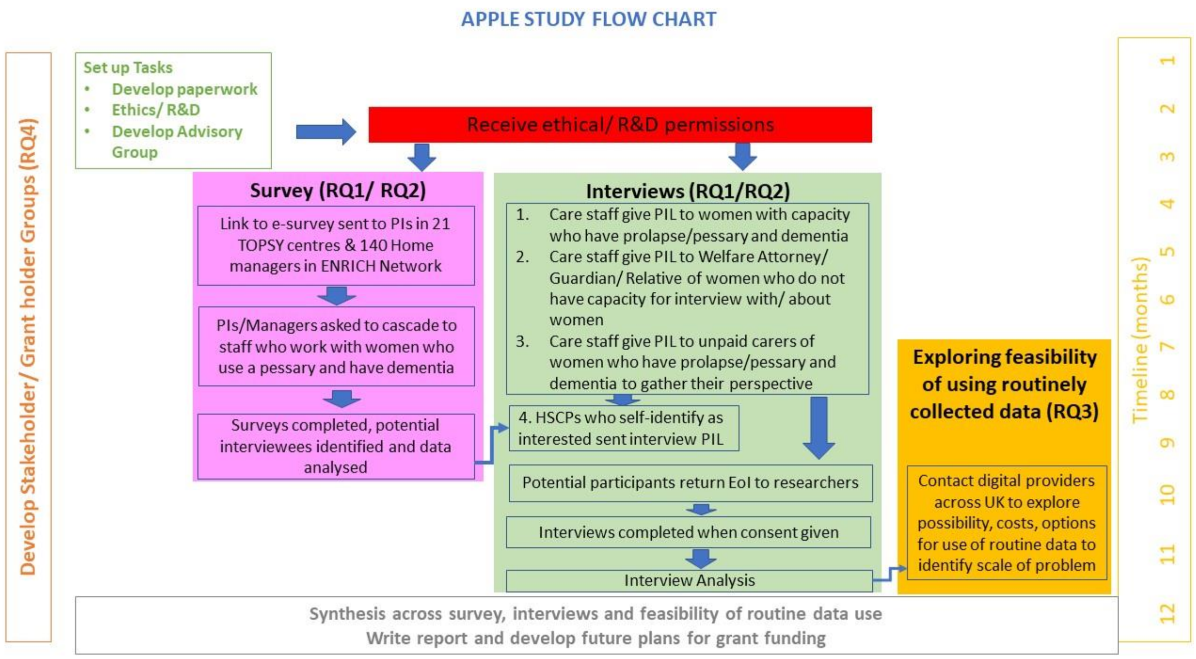

This annex considers the survey (in the pink box) and the interviews where they apply only to health and social care professionals (the green box).

1.0 Background and Rationale

This study aims to investigate a significant problem for practice; specifically, best practice for women with dementia who use a pessary for treatment of pelvic organ prolapse.

What is the problem being addressed?

The NICE guidelines suggest that up to 50% of women show pelvic organ prolapse upon examination (NICE 2019). One small, US based, study identified that 32% of women aged over 55 seeking management of a pelvic floor disorder have been reported to have either early dementia or mild cognitive impairment (Ferreira et al, 2016). The scale of the problem of women who have dementia and a pelvic floor disorder is not known, and no data are available for the UK.

The Dementia Research Roadmap (2018) outlines a key target of improving the lives of people with dementia. No literature has been identified that has investigated the development or evaluation of interventions that aim to improve the quality of life for women with dementia who have pelvic organ prolapse. This proposal aims to address the problem of understanding the scale of the problem and how best to provide health care for women who use a pessary for pelvic organ prolapse and who also have dementia.

Why is the research important in terms of improving the health of people with dementia?

Prolapse is known to cause women to experience bothersome symptoms (Harvey et al, 2021). It is not known the extent to which women with dementia and prolapse get help to alleviate those symptoms nor how services meet their needs. Women who use a vaginal pessary to manage prolapse either self-manage their pessary or attend a clinic for pessary changes. Women with dementia may struggle to access pessary care support and may be more likely to be lost to follow up which can result in complications secondary to pessary neglect including fistula, sepsis and death (Bae et al, 2021; Gauta et al, 2017; Ranka et al, 2008). Healthcare professionals (HCPs) lack information on the needs and wishes of women with dementia for prolapse treatment. This gap in knowledge makes it difficult for HCPs to know how best to care for women with a pessary when they lose capacity. If capacity is impaired it often presents challenges to HCPs to ensuring regular pessary changes and decision making around surgical and/or conservative intervention. As the service needs of the population may be different it is important to explore service needs for this population specifically.

How does the literature support this proposal?

A search of Medline, CINAHL and Embase databases was undertaken using the terms "pessary" AND "dementia" on 20th October 2021. Five relevant publications consisting of four case reports and a conference abstract detailing secondary analysis of a prospective, cross-sectional study of older female patients receiving treatment for pelvic floor disorders were identified. Each of the four case reports detailed complications where women with dementia experienced pessary complications which required surgical correction (Bhat et al, 2021; Bae et al, 2021; Ranka et al, 2008; Gauta, 2017). It has been established that individuals with dementia occasionally or

frequently miss healthcare appointments (Wang et al, 2015). Ranka et al (2008) suggest this means women with dementia using pessary are at increased risk of serious complication, and therefore require follow-up in the community should they fail to attend a pessary hospital appointment. Ferreira et al (2016) found that although treatment decisions about their pelvic floor disorder did not differ between women with dementia, cognitive impairment or normal cognition, women with normal cognition were significantly more likely to use vaginal oestrogen replacement, thus indicating that treatment between women who have and who do not have dementia may be unequal. ‘First Do No Harm’ (IMMDS 2020) recommended that information should be given in a way that is understandable and proportionate to a woman’s circumstances, but HCP are conflicted what this information should be in the circumstance of a woman having dementia. Pessary care requires regular attendance at clinic appointments for a pessary change unless the woman can self-manage. Safe pessary care relies on the woman alerting a HCP if she experiences symptoms of complications such as vaginal bleeding or excessive discharge. Women with dementia are at higher risk of missing hospital appointments, being lost to follow-up and experiencing severe complications due to pessary neglect, including incarcerated pessary requiring removal under anaesthetic, vesicovaginal and rectovaginal fistulae and vaginal cancer, a rare but reported risk of pessary incarceration. Carers may be unaware of the importance of observing for vaginal bleeding or abnormal vaginal discharge. Clinicians involved in pessary management are uncertain how to manage women with dementia who use pessaries. The impact of repeated clinic appointments for vaginal examination if the woman lacks capacity to consent, versus no treatment or surgery needs to be evaluated and there is insufficient information available about what women with dementia want for prolapse management to inform these difficult decisions.

2.0 Objectives to be addressed in this annex

1. To understand which services currently deliver pessary care for women with dementia and how those services manage/support the women and their unpaid carers.

3.0 Study Design

This project will deliver data that supports the development of a study which will in turn aim to a) improve the lives of women with dementia; and b) development of best practice guidance for healthcare professionals that will address an ethical dilemma for practitioners in practice. This impact is important as people with dementia are recognised to be a marginalised group in research and for whom the evidence to support practice is lacking. A multi-methods approach will be used to deliver on the identified objectives. This annex only addresses research question one. The flow chart above outlines the feasibility study as a whole, this annex only addresses the survey to HSCP (pink box) and interviews with HSCP (parts of the green box).

3.1 Research Question 1.

3.1.1 Sample and Recruitment

The sample will be key stakeholders, specifically Health Care Professionals who deliver pessary care for women with dementia and prolapse in hospital and community settings and social care/third sector staff (trained and untrained) who deliver care for women with dementia and prolapse in hospital and community settings.

Eligibility Criteria

Table One below outlines the inclusion and exclusion criteria.

Table 1: Inclusion and Exclusion Criteria

	Inclusion criteria	Exclusion criteria
HSCP	• Works with women who have prolapse<br>• Experience of supporting women who has/had a vaginal pessary for prolapse<br>• Has experience of working with women who have dementia<br>• Works in any care or community (including women's homes) setting<br>• Works within the UK<br>• English speaking	• No experience of working with women who have prolapse or pessary or dementia

3.1.2 Interview Sampling

Size of sample

We will aim to recruit 10-15 participants across the groups, a sample that should be large enough to give information power for the research questions asked.

Sampling technique

Purposive sampling for maximum variation. We are aiming for HSCP variation in: working in a range of care settings; different professional backgrounds; different genders; specialist/non-specialist; geographical variation (across the UK)

3.1.3 Recruitment

Sample identification

Based on the advice of the advisory group we will work with the TOPSY infrastructure and approach staff from the TOPSY sites. We will also approach care home staff and other NHS/social care sites who have experience of working with women with dementia and prolapse.

A link to the survey will be sent to the Principal Investigator (PI) at each of the 21 TOPSY centres. The PI will be asked to complete and cascade the survey link to members of staff at their centre who deliver pessary care and/or are providing care to women with dementia. The survey will also be sent to the home manager in care homes within the ENRICH network who will also be asked to cascade to members of staff.

Staff who self-identify as interested in finding out more about being interviewed will be sent a recruitment email that contains:

- Introductory letter
- Participant information leaflet

If potential participants remain interested they will respond to researchers directly.

Consent Pathway

The pathway below is for Health and Social Care professionals who have professional caring responsibilities for women who have dementia and use a pessary for prolapse.

PATHWAY FOR HSCP

indicates interest in being interviewed in the survey and provides contact email address

↓

Researcher will email PIL and consent form to HSCP who expressed interest in interview and offer to answer any questions

↓

Participant asked to return consent form the qual researcher if willing to take part

↓

Researcher receives consent form (CONSENT form)

Qual researcher will arrange a date and time for a MS Teams/telephone interview

3.1.3 Data Collection

To address objectives one and two we will: undertake a short survey with HSPCs and interview HSCPs.

HSPC survey

We have developed a short survey that will be sent by email, with a link to survey, to the PIs in each of the TOPSY centres. We will also send the survey to care homes within the ENRICH network. The survey explores the scope and scale of the problem from the perspectives of treating sites.

HSPC Interviews

HSCP will be interviewed by MS Teams and the interview will last approximately 30 minutes. Interviews will be semi-structured. They will be asked about their experience in providing pessary care to women with dementia. All interviews will be downloaded onto a secure server as soon as possible after the interview and the recorder wiped. Interviews will be transcribed with all identifying information removed then entered into NVivo for data management. Data will be stored in line with GCUs Information Classification and Handling Policy (https://www.gcu.ac.uk/aboutgcu/supportservices/it-services/itregulationsandpolicies)

3.1.4 Data Analysis

Survey

Descriptive statistics will be used to report on survey findings. The survey will have some open questions where HSCP are encouraged to provide more detailed feedback on their experiences of providing care to women with dementia who use/have used a pessary. Free-text answers provided within the survey will be analysed using template analysis (Brooks and King 2014), a form of thematic analysis that allows the use of an *a priori* grid which will be applied to the data while remaining flexible to be revised and refined (Brooks and King 2014).

Interviews

A reflective thematic analysis framework, drawing on Braun and Clarke's (2006, 2013, 2019) work will be applied to the collected interview data for analysis. Thematic analysis has the benefit of being exceptionally flexible while providing a rich and detailed account. We will follow the six phases outlined in Braun and Clarke's 2013 book:

1. Familiarisation with data: reading and re-reading interview transcripts, noting down initial ideas.
2. Generating initial codes: Coding interesting features of the data in a systematic fashion across the entire data set, collating data relevant to each code.
3. Searching for themes: Collating codes into potential themes, gathering all data relevant to each potential theme.
4. Reviewing themes: Checking if the themes work in relation to the coded extracts (Level 1) and the entire data set (Level 2), generating a thematic 'map' of the analysis.
5. Defining and naming themes: Ongoing analysis to refine the specifics of each theme, generating clear definitions and names for each theme.

6. Producing the report: Selection of compelling data extracts

Reflexivity will be applied throughout each of the six phases outlined above to acknowledge and counteract any underlying assumptions brought to the analysis process by the researchers.

4.0 Ethics and Dissemination

The sub-study is being submitted as an amendment to the TOPSY study ethics approval (17/WS/0267). A concurrent application has been submitted to Scotland A REC for review for permissions to interview women with dementia (who do and do not have capacity to consent) and their informal carers.

Dissemination will follow the TOPSY study publication protocol.

5.0 Timetable

The timetable is presented in the table below.

Table 2: Study timetable

Month	1	2	3	4	5	6	7	8	9	10	11	12
Set up tasks:<br>- Advisory group<br>- Ethics<br>- R&D approvals	■	■	■									
Interview Data Collection		■	■	■	■	■	■	■				
Interview data Analysis			■	■	■	■	■	■	■	■		
Explore feasibility of datasets/ data linkage			■	■	■	■	■	■	■	■		
Prepare report										■	■	■
Advisory Group Meetings	■				■				■			■
Future research team development										■	■	■

6.0 Management and Governance

All GCU governance procedures will be adhered to, including for GDPR.

The study CI (CB) and the study RF (MD) will meet biweekly to monitor study progress. The core team involved will meet monthly and the PMG will meet 3-monthly. The timetable above will be used to monitor progress against targets.

We will invite PPIE participants and other key stakeholders to be part of an advisory group. The advisory group have been asked to comment on recruitment pathways. The advisory group will meet formally 4 times but informal communication between the RF and the advisory group will be maintained throughout the study.

References

ALZHEIMERS UK. 2018. Dementia research roadmap for prevention, diagnosis, intervention and care by 2025 An opportunity to align national dementia strategies and research Alzheimer's Society, January 2018 Dementia research roadmap for prevention, diagnosis, intervention and care by 2025 (alzheimers.org.uk) [Accessed 21.10.21]

BAE HA, CHOI JI, CHUNG SH, SANG JH. 2021. Migration of neglected vaginal pessaries: A case report. *International Journal of Women's Health and Reproduction Sciences,* 9**,** 225-227.

BHAT M, ACHARYA S, AGUR W. 2021. Vesico-vaginal fistula presenting as overactive bladder in a case of Gellhorn pessary for vault prolapse. *BMJ Case Reports,* 14**,** e240331.

BRAUN V, CLARKE V. 2021. *Thematic analysis: a practical guide*. SAGE Publications.

BRAUN V. & CLARKE V. 2006. Using thematic analysis in psychology. *Qualitative Research in Psychology* 77–101. https://doi.org/10.1191/1478088706qp063oa

BROOKS J, KING N. 2014. *Doing template analysis: evaluating an end-of-life care service*. SAGE. Retrieved September 28 2022 from https://doi.org/10.4135/978144627305013512755.

CUMBERLEGE J. 2020. First do no harm: the report of the Independent Medicines and Medical Devices Safety Review. 2020. Available at: https://www.immdsreview.org.uk/Report.html. [Accessed 19 July 2022].

FERREIRA ET, BACHI A, ABERNETHY M, KUNKLE CM, CHEN CG. 2016. The effect of cognitive impairment on pelvic floor treatment decisions. *Journal of the American Geriatrics Society,* 64**,** S259.

GAUTA J. 2017. Pessary migration into the bladder: a rare episode of removal and repair of a vesicovaginal fistula. *International Urogynecology Journal,* 28**,** 965-966.

HARVEY MA, CHIH HJ, GEOFFRION R, AMIR B, BHIDE A, MIOTLA P, ROSIER PFWM, OFFIAH I, PAL M, ALAS AN. 2021 International Urogynecology Consultation Chapter 1 Committee 5: relationship of pelvic organ prolapse to associated pelvic floor dysfunction symptoms: lower urinary tract, bowel, sexual dysfunction and abdominopelvic pain. Int Urogynecol J. Oct;32(10):2575-2594. Epub 2021 Aug 2.

National Institute for Health and Care Excellence (NICE). Urinary incontinence and pelvic organ prolapse in women: Management. 2019. Available at: https://www.nice.org.uk/guidance/ng123. [Accessed 19 July 2022].

RANKA P, TOOP K, POPLI K. 2008. An entrapped shelf pessary. *Scottish Medical Journal,* 53**,** 1-3.

SAMSI K, MANTHORPE J. 2020. Interviewing people with dementia: Methods review. *NIHR School for Social Care Research.*

SCOTTISH DEMENTIA WORKING GROUP RESEARCH SUB-GROUP (2014). 'Core principles for involving people with dementia in research: innovative practice'. *Dementia,* 13(5)**,** pp. 680-685.

WANG X, ROBINSON KM, HARDIN HK. 2015. The Impact of Caregiving on Caregivers' Medication Adherence and Appointment Keeping. *Western Journal of Nursing Research,* 37**,** 1548-1562.
